# Genetics and breeding for resistance against four leaf spot diseases in wheat (*Triticum aestivum* L.)

**DOI:** 10.3389/fpls.2023.1023824

**Published:** 2023-03-29

**Authors:** Pushpendra Kumar Gupta, Neeraj Kumar Vasistha, Sahadev Singh, Arun Kumar Joshi

**Affiliations:** ^1^Department of Genetics and Plant Breeding, Chaudhary Charan Singh University, Meerut, India; ^2^Murdoch’s Centre for Crop and Food Innovation, Murdoch University, Murdoch, WA, Australia; ^3^Borlaug Institute for South Asia (BISA), National Agricultural Science Complex (NASC), Dev Prakash Shastri (DPS) Marg, New Delhi, India; ^4^Department of Genetics-Plant Breeding and Biotechnology, Dr Khem Singh Gill, Akal College of Agriculture, Eternal University, Baru Sahib, Sirmour, India; ^5^The International Maize and Wheat Improvement Center (CIMMYT), National Agricultural Science Complex (NASC), Dev Prakash Shastri (DPS) Marg, New Delhi, India

**Keywords:** wheat, pathogens, sensitivity genes, resistance genes, necrotrophic effectors, PR proteins

## Abstract

In wheat, major yield losses are caused by a variety of diseases including rusts, spike diseases, leaf spot and root diseases. The genetics of resistance against all these diseases have been studied in great detail and utilized for breeding resistant cultivars. The resistance against leaf spot diseases caused by each individual necrotroph/hemi-biotroph involves a complex system involving resistance (R) genes, sensitivity (S) genes, small secreted protein (SSP) genes and quantitative resistance loci (QRLs). This review deals with resistance for the following four-leaf spot diseases: (i) Septoria nodorum blotch (SNB) caused by *Parastagonospora nodorum*; (ii) Tan spot (TS) caused by *Pyrenophora tritici*-*repentis*; (iii) Spot blotch (SB) caused by *Bipolaris sorokiniana* and (iv) Septoria tritici blotch (STB) caused by *Zymoseptoria tritici*.

## Introduction

**Figure 1:**
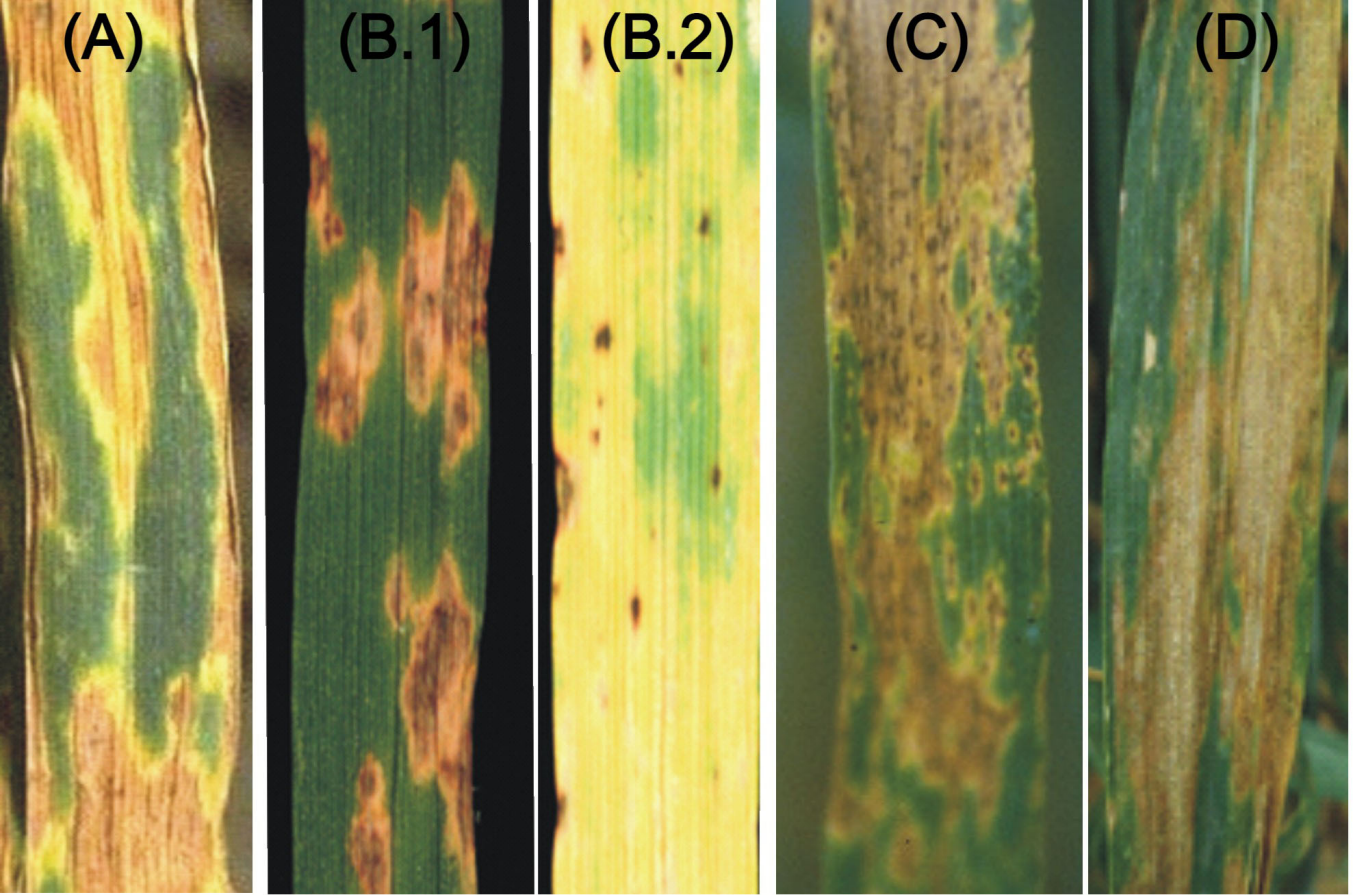
The visual symptoms of four necrotrophic and hemibiotrophic diseases. **(A)** SNB; **(B)** TS (**B.1** and **B.2** showing necrosis and chlorosis); **(C)** SB and **(D)** STB.

Wheat (*Triticum aestivum* L.) is the third most important staple food crop worldwide (the other two being maize and rice). According to FAO, during 2021-22, the total global wheat grain production was 778.6 million tonnes as against ~697 million tonnes in the year 2011-12, and 756.5 million tonnes in 2016-17 giving an annual increase of a mere 1.24% over the last 10 years and 0.83% over the last five years showing a decline in annual growth rate as against the desired rate of ~1.5% - 2% to meet the demand of growing world population. A variety of biotic and abiotic stresses are responsible for this bottleneck. The biotic stresses mainly include pathogens like fungi, viruses, bacteria, and nematodes, which cause a variety of diseases. Among these pathogens, fungal pathogens cause diseases like rusts, mildew, blast, bunts, and blights, which are responsible for 15-20% yield loss (Figueroa et al., 2018). Among major classes of wheat diseases, the following are the four important leaf spot diseases, which are also described as blotch diseases (**Figure 1**): (i) Septoria nodorum blotch (SNB) caused by *Parastagonospora nodorum*, (ii) Tan spot (TS) caused by *Pyrenophora tritici*-*repentis*, (iii) Spot blotch (SB) caused by *Bipolaris sorokiniana* and (iv) Septoria tritici blotch (STB) caused by *Zymoseptoria tritici*. Among these four pathogens, *P. nodorum* and *P. tritici-repentis* are necrotrops, while *B. sorokiniana* and *Z. tritici* are hemi-biotrophs, since these are believed to need living tissue initially and later kill the host tissues and then feed and survive on the dead tissues of the host. The hemi-biotrophic nature of *Z. tritici* has, however, been recently questioned (Sanchez-Vallet et al., 2015; Friesen and Faris, 2021).

**Figure 2:**
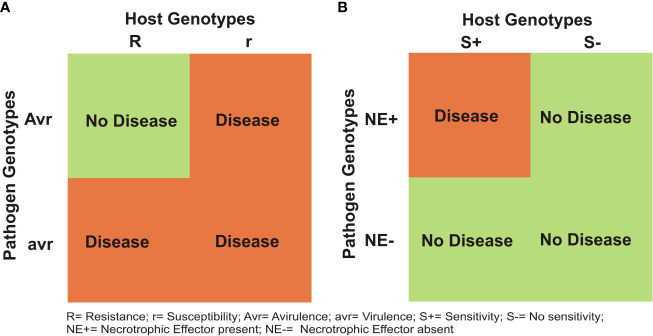
Two different models for host- pathogen interactions in plants: **(A)** gene-for gene (GFG) model, proposed by Flor (1956) and **(B)** an inverse gene-for gene (IGFG) model that was discovered recently in a number of necrotrophic diseases in wheat.

It has been shown that for most diseases in all crops including wheat, a gene-for-gene (GFG) model holds good between individual R genes of the host and the matching Avr genes in the pathogen (Flor, 1942; Flor, 1956). As a result, in the absence of a matching avirulance (Avr) gene in the prevalent race of the pathogen, the R gene can not provide resistance (**Figure 2A**). In contrast, the inverse gene-for-gene (IGFG) model (Fenton et al., 2009) assumes that a compatible interaction requires the presence of a matching susceptibility/S gene in the host and the corresponding necrotrphhic effector (NE) gene in the pathogen, so that in the absence of matching S gene in the host, an infection can not occur (**Figure 2B**; Friesen et al., 2007). Current knowledge suggests that in the same crop, both GFG and IGFG systems may operate synergistically, although this has not been widely discussed. Another class of genes include small secreted protein (SSP) genes, which have recently been shown to provide resistance against *Z. tritici* (Zhou et al., 2020); these SSP genes in wheat for other diseases have yet to be discovered. Quantitative disease resistance (QDR) may also occur together with S genes, R genes and SSP genes, although in some cases, one or more quantitative resistance loci (QRLs) have also been shown to represent S/R genes (for QRLs, see later). This makes the genetic systems for resistance against individual necrotrophs and hemi-biotrophs rather complex, but an interesting subject for research.

A number of studies have also been conducted on QDR against each of the above four diseases. These studies mainly include identification of either the QRLs using linkage-based interval mapping, often involving biparental mapping populations [sometimes also involving multi-parent advanced generation inter-cross (MAGIC) populations] or the marker-trait associations (MTAs) using LD-based genome-wide association studies (GWAS) using association panels. In some cases, a QRL identified through interval mapping may also overlap a resistance R gene, as shown in the case of one or more of the four R genes (*Sb1-Sb4*) for resistance against *B. sorokiniana* (Kumar et al., 2015; Gupta et al., 2018a). The relative roles of QRLs/R genes and the sensitivity S genes have also been assessed, and it was shown that QRLs/R and not the S genes are the major source of resistance, although in certain parts of the world, resistance has also been found to be associated with absence of S genes like *Tsn1* (Cowger et al., 2020).

The occurrence of multiple disease resistance (MDR) involving resistance against more than one disease has also been reported (Zwart et al., 2010; Mago et al., 2011; Singh et al., 2012; Jighly et al., 2016; Pal et al., 2022). As an example, MDR for SNB and TS has been reported in some winter wheat cultivars, suggesting that MDR may be associated with winter habit and that diverse sources of resistance for multiple diseases can be made to hybridize to achieve MDR (Ali et al., 2008; Gurung et al., 2009; Gurung et al., 2014). Association among resistance to two or three diseases, namely SNB, SB, and STB has also been observed (Gurung et al., 2014). Such an association involving MDR could also be the result of unconscious selection for resistance to multiple diseases during wheat breeding programmes. GWAS-based MTAs, each associated with more than one disease, have also been identified, suggesting occurrence of either the pleiotropic genes or closely linked loci providing resistance to more than one disease (Gurung et al., 2014).

Meta-QTLs involving diseases caused by more than one necrotroph have also been recently identified (Saini et al., 2022). However, while the available literature on interactions between S genes and necrotrophic effectors (NEs), and also on cloning and characterization of the sensitivity genes in the host and the NE genes in the pathogen has been the subject of several reviews, the literature on the complex genetic system for disease resistance involving S genes, R genes, SSP genes and QRLs associated with more than one disease caused by necrotrophs has not been adequately reviewed (Cowger et al., 2020). However, it has been shown that durable resistance against the pathogen is generally achieved through a quantitative genetic system and that interactions between the products of the recessive alleles of S genes and the NEs play a minor role in providing resistance (Cowger et al., 2020). The S genes, S QTLs, R genes, and resistance QTLs identified through QTL interval mapping and GWAS are sown in **Supplementary Figures S1**, **S2**.

The present review is intended to provide an overview of the complex genetic system of resistance in wheat against each of the four different pathogens, mentioned above. While doing so, we recognize that the work involving GFG in biotrophs is widely known and regularly reviewed (Van Der Biezen and Jones, 1998; Kaur et al., 2021). Therefore, in this review, emphasis will be on the NEs and S genes involved in IGFG. In each case, the disease is caused by an interaction between NEs encoded by host-specific toxin (HST) genes of the pathogens and the proteins encoded by the corresponding S genes in the host, but resistance is largely quantitative in nature. The recent information on the genomics of all four pathogens will also be included in this review since whole genome sequences are now available for all four pathogens; this became possible only due to the availability of high through-put next-generation sequencing (NGS) technology (Syme et al., 2013; Moolhuijzen et al., 2018; Plissonneau et al., 2018; Richards et al., 2018; Syme et al., 2018; Aggarwal et al., 2019; Aggarwal et al., 2022). The present review on genetics and breeding for resistance against four leaf spot diseases in wheat should prove useful for geneticists, breeders and pathologists for further research targeted towards development of high yielding cultivars, resistant to these four foliar leaf spot diseases.

## Pathosystems and genetics of disease resistance

As mentioned above, among the four pathosystems selected for this review, two pathosystems involve necrotrophs, while the other two involve hemi-biotrophs. The major differences between biotrophs and necrotrophs are listed in **Table 1**. The details of the four important pathogens (necrotrophs/hemi-biotrophs) and the wheat diseases caused by them are listed in **Table 2**. For each of the four diseases, S genes, R genes and QRLs identified either through interval mapping or through GWAS are summarised in **Supplemantery Table S1**, which also includes information on SSP genes for resistance against STB. More details for each of the four pathosystems under review along with genetics of disease susceptibility/resistance as well as interactions between S genes and NE genes are presented in this section.

**Table 1:** A comparison of characteristics of biotrophs and necrotrophs (based on the link http://www.davidmoore.org.uk/21st_Century_Guidebook_to_Fungi_PLATINUM/Ch14_10.htm).

Biotroph pathogens	Necrotroph pathogens
Appressoria or haustoria produced	Appressoria/haustoria normally not produced
Resistance is controlled by SA-dependent host-defense pathways	Resistance controlled by SA, JA and Et-dependent host-defense pathways
Gene-for-gene (GFG) relationship	Inverse gene-for-gene (IGFG) relationship
Difficult to culture	Easy to culture
Entry, direct or through natural openings	Entry via wounds or natural openings
Survive on living tissues or as dormant propagules	Survive on living/dead tissue or as competitive saprotrophs
Host cells not killed rapidly; hypersensitive reaction (HR) in resistant genotype	Host cells killed rapidly; no hypersensitive reaction in resistant genotype
Few lytic enzymes/toxins are produced	Cell-wall-degrading (lytic) enzymes/toxins are produced
Often systemic	Seldom systemic
Attack vigorous plants; any stage	Attack weak, young/damaged plants
Narrow host range	Wide host range
Intercellular/Intracellular growth of pathogen	Intercellular/Intracellular growth of pathogen
Effectors: Avr proteins recognized by matching resistance (R) proteins	Effectors: host-specific or host-selective toxins (HST)
Disease caused by suppressing PTI/ETI	Disease caused either by suppressing PTI/ETI or by activation of sensitivity genes

**Table 2:** Necrotrophs and hemibiotrophs causing diseases in wheat.

Pathogen (teleomorph)	Pathogen (anamorph)	Disease caused	Reference
*Parastagonospora nodorum* (Berk.) Quaedvlieg, Verkley & Crous	*Stagonospora nodorum* [Berk.] Castellani & E.G. Germano)	Septoria nodorum blotch (SNB)	Weber, 1922; Sprague, 1950; King et al., 1983; Scharen et al., 1985
*Pyrenophora tritici-repentis* (Died.) Drechsle	*Drechslera tritici*-*repentis* (Died.) Shoemaker	Tan spot	Drechsler, 1923; Shoemaker, 1959; Shoemaker, 1962
*Cochliobolus sativus* (S. Ito & Kurib.) Drechsler ex Dastur	*Bipolaris sorokiniana (*Sorokin) Shoemaker	Spot Blotch (SB)	Dastur, 1942; Shoemaker, 1959
*Mycosphaerella graminicola* (Fuckel) J. Schröt.	*Zymoseptoria tritici* (Desm.) Quaedvlieg & Crous	Septoria tritici blotch (STB)	Desmazieres, 1842; Schroter, 1894

### *P. nodorum*-wheat pathosystem

The *P. nodorum*-wheat pathosystem involved in the disease SNB is the most extensively studied pathosystem involving necrotrophs. Therefore, it is also used as a model to study host-pathogen interactions involving NEs previously referred to as host selective toxins (HSTs), released by the pathogen and the products of S genes in the host. The disease SNB includes both leaf blotch and glume blotch (**Figure 1A**) that are common in warm and humid areas of the world, causing ~16% yield losses, which sometimes approach 60% under severe infection/epidemic conditions (Bhathal et al., 2003; Ficke et al., 2018; Shankar et al., 2021). The disease is particularly common in Australia, USA, parts of Europe and southern Brazil. The short incubation period enables the pathogen for multiple infection cycles within a season. The fungus can reproduce through asexual conidia as well as through sexual reproduction due to the availability of both mating types (MAT1-1 and MAT1-2).

### NE genes, S genes, and the interactions

*P. nodorum* produces NEs, which contribute to variation in aggressiveness. The infection occurs only when a specific S gene of the host responds to the presence of a NE encoded by a gene in the pathogen. Nine S genes in the host and eight NE genes in the pathogen have been identified, which are involved in the following nine interactions: (i) *Tsn1-*SnToxA; (ii) *Snn1-*SnTox1; (iii) *Snn2-*SnTox2; (iv) *Snn3B1-*SnTox3; (v) *Snn3D1-*SnTox3 (vi) *Snn4-*SnTox4; (vii) *Snn6-*SnTox6; (viii) *Snn7-*SnTox7; (ix) *Tsn1-*SnToxA. A new NE named SnTox267, was later shown to represent three previously characterized NEs, namely SnTox2, SnTox6 and SnTox7, hence the name SnTox267 (Richards et al., 2021). Among the above nine interactions, the following interactions have been subjected to relatively detailed studies because the S genes and the NE genes involved in these interactions have all been cloned and characterized: *Tsn1*-*SnToxA, Snn1*-*SnTox1* and *Snn3-D1*-*SnTox3*.

The distribution of S genes involved in SNB in wheat populations differs in different wheat-growing regions of the world. However, maximum data is available from the USA, Europe (including UK and Norway) and Western Australia. The distribution of three NE genes (*SnToxA*, *SnTox1* and *SnTox3*) in a globally diverse collection of pathogen isolates, was reported by McDonald et al. (2013); the results are summarised in **Table 3**. These results, suggested that the gene *SnTox1* is the most widely distributed gene, occurring in 95.4% isolates from the USA (as above) and in 84% isolates worldwide. Similar frequencies (85%) were reported for the corresponding S gene *Snn1* in wheat germplasm; this was also confirmed in some independent surveys conducted for the distribution of different S genes in wheat cultivars (Tan et al., 2014; Phan et al., 2018; Hafez et al., 2020).

**Table 3:** Distribution (%) of three Tox genes among isolates of *P. nodorum* and sensitivity genes in wheat.

Distribution (%) of three Tox genes in *P. nodorum*				
Region	*SnToxA*	*SnTox1*	*SnTox3*	Reference
Fertile Crescent	95.0	97.0	72.0	Ghaderi et al., 2020
Norwegian	67.9	46.1	47.9	Lin et al., 2020b
Canada	69.2	80.7	76.9	Hafez et al., 2020
Norwegian	69.0	53.0	76.0	Ruud et al., 2018
World-wide collection	18.0	26.0	22.0	McDonald et al., 2013
Europe	12.0	89.0	67.0	McDonald et al., 2013
South-eastern United States	15.0	74.0	39.0	Crook et al., 2012
Distribution (%) of three sensitivity related genes in wheat				
	*Tsn1*	*Snn1*	*Snn3*	
Norwegian	30.0	7.6	15.3	Lin et al., 2020b
Canada	59.0	32.9	56.9	Hafez et al., 2020
Norwegian	45.0	12.0	55.0	Ruud et al., 2018
Russia	29.3	26.8	51.2	Phan et al., 2018
Kazakhstan	27.7	28.6	63.6	Phan et al., 2018
India	66.7	58.3	77.8	Phan et al., 2018
Pakistan	59.4	71.9	68.8	Phan et al., 2018
British French, German and Dutch	9.1	28.0	42.0	Downie et al., 2018
Australia	63.0	71.7	91.3	Tan et al., 2014
USA	32.0	0.0	64.0	Bertucci et al., 2014

### Cloning of S genes and NE genes

Three S genes (*Tsn1*, *Snn1*, and *Snn3-D1*) and five NE genes (*SnToxA*, *SnTox1*, *SnTox3*, *SnTox5*, *SnTox267*) have also been cloned and characterized thus permitting a study of interactions between the products of sensitivity genes and NEs at the molecular level. Efforts are also underway for cloning of *Tsc1* gene. For this purpose, in a recent study, 58 molecular markers were identified delineating a 1.4 cM genetic interval spanning 184kb on chromosome 1AS, carrying *Tsc1* gene (Running et al., 2022). This short region carried only nine candidate genes that were mainly related to NB-ARC, protein kinase, LRR, retinal pigment epithelial membrane protein and pseudo-gliadin proteins. The information with details of the cloned S genes and NE genes is available in a number of individual original papers (Ciuffetti et al., 1997; Liu et al., 2009; Faris et al., 2010; Liu et al., 2012; Liu et al., 2016; Shi et al., 2016a; Shi et al., 2016b; Kariyawasam et al., 2021; Zhang et al., 2021) and summarized in several recent reviews (Wang et al., 2014; McDonald and Solomon, 2018; Faris and Friesen, 2020; Friesen and Faris, 2021; Lin and Lillemo, 2021; Peters Haugrud et al., 2022). The information is also summarized in **Table 4** and **Supplementary Figure S3**.

**Table 4:** A summary of cloned sensitivity and NE genes involved in SNB.

Gene/chromosome	Length (bp)	Protein (aa)	Reference	Figure
Sensitivity genes (wheat, *T. aestivum*)				
*Tsn1* (5BL)	10,581	S/TPK-NBS-LRR	Faris et al., 2010	**Supplementary Figures S3A, B**
*Snn1* (1BS)	13,045	GUB-WAK, EGF_CA, TM, PK	Shi et al., 2016b	**Supplementary Figures S3C–F**
*Snn3−D1* (5DS)	1,977	PKMSP	Zhang et al., 2021	**Supplementary Figure S3G**
NE Genes (*P. nodorum*)				
*SnToxA*	534	13 kD	Ciuffetti et al., 1997	**Supplementary Figure S3H**
*SnTox1*	7,600	10.3 kD	Liu et al., 2012	**Supplementary Figures S3I, J**
*SnTox3*	693	25.8 kD	Liu et al., 2009	**Supplementary Figures S3L, M**
*SnTox5*	654	16.26 kDa	Kariyawasam et al., 2021	**Supplementary Figure S3K**
*Sn267*	2,086	74.5 kDa	Richards et al., 2021	**Supplementary Figure S3N**

### Genetics of resistance

There are at least three genetic systems, which provide resistance against SNB, as also in other pathosystems; these three systems include the following: (i) recessive alleles or loss of S genes, (ii) classical R genes and (iii) QTLs/MTAs identified through interval mapping and GWAS. Some details of these three systems will be described.

#### S genes for resistance.

According to Cowger et al. (2020), some evidence is available, which suggests that during breeding programs, perhaps unconscious selection for resistance has been exercised against S genes (*Snn* genes in the case of *P. nodorum*) due to their role in conferring susceptibility. It is also assumed that some S genes were R-genes, which provided resistance against pathogens, but have been hijacked/corrupted by necrotrophs to provide susceptibility, thus becoming S genes (Nagy and Bennetzen, 2008; Faris et al., 2010; Gilbert and Wolpert, 2013; Shi et al., 2016b). For instance, during the cloning of *Tsn1* and *Snn1* genes, it was concluded that necrotrophs hijacked the R genes involved in resistance to biotrophs and altered them for their own benefit (Faris et al., 2010; Shi et al., 2016b). During interval mapping also, some QTLs were found to be located in the genomic regions occupied by S genes, thus suggesting that QTLs may also sometimes represent R genes hijacked by the pathogens.

#### Possible R genes

Wheat genome sequences were also utilized for the identification of R genes associated with the genomic regions occupied by QTLs that were earlier identified and mapped on 1BS and 5BL. The annotation of intervals in the reference sequence allowed identification and mapping of 13 R genes on 1BS and 12 R-genes on 5BL (Li D. et al., 2021), although no evidence was available showing that these R genes were involved in providing resistance against SNB. The analysis of R genes, however, resolved co-located QTL on 1BS into the following two distinct but linked loci: (i) *NRC1a* and *TFIID* mapped in one QTL on 1BS, and (ii) *RGA* and *Snn1* mapped in the linked locus; all these genes were found to be associated with SNB resistance, but only in one environment. Similarly, *Tsn1* and *WK35* were mapped on the same QTL on 5BL, with NETWORKED 1A and RGA genes mapped in the linked QTL interval.

#### QTLs/MTAs for SNB resistance (leaf blotch, and glume blotch)

As mentioned above, *P. nodorum* is responsible for two SNB diseases in wheat, *i.e.*, leaf blotch and glume blotch (involving flag leaf for leaf blotch and spikes for glume blotch). The inheritance pattern for resistance against the two diseases differs (Wicki et al., 1999; Xu et al., 2004; Shankar et al., 2008; Chu et al., 2010). However, in several genetic studies, no distinction was made between leaf blotch and glume blotch. QTLs for resistance against SNB have also been identified following both linkage-based interval mapping and LD-based GWAS.

Interval mapping involved both bi-parental and multi-parental (MAGIC) mapping populations, and resulted in the identification of ~170 QTLs including ~30 major QTLs, each explaining >20% of the phenotypic variation (**Supplementary Table 2**). These studies included the following: Czembor et al., 2003; Schnurbusch et al., 2003; Arseniuk et al., 2004; Liu et al., 2004a; Aguilar et al., 2005; Reszka et al., 2007; Uphaus et al., 2007; Shankar et al., 2008; Friesen et al., 2009; Gonzalez-Hernandez et al., 2009; Francki et al., 2011; Abeysekara et al., 2012; Shatalina et al., 2014; Ruud et al., 2017; Francki et al., 2018; Czembor et al., 2019; Singh et al., 2019; Lin et al., 2020a; Lin et al., 2021; (for a recent review, also see Downie et al., 2021).

The above QTL studies also included two recent major studies, each involving an independent MAGIC population, one used by Lin et al. (2020a) and the other used by Lin et al. (2021). Using these two MAGIC populations, 17 QTLs on the following chromosomes were identified: 2A, 2D, 5A, 5B, 6A, 7B and 7D. In these two studies, two QTLs, namely *QSnb.niab-2A.3* (UK MAGIC population, Lin et al., 2020a) and *QSnb.nmbu-2A.1* (German MAGIC population, Lin et al., 2021) were found in a short interval on chromosome 2A; these two QTLs could represent a hot spot controlling resistance against SNB. A QTL (*QSnb.niab-5B.2*) overlapping *Tsn1* was also identified on 5BL (Lin et al., 2020a; Lin et al., 2021).

The GWA studies for resistance to SNB were undertaken both at the seedling stage and adult plant stage (flag leaf and spike for glume blotch) and resulted in identification of MTAs on almost all 21 chromosomes. However, more studies were conducted at the seedling stage than at the adult stage (glume blotch). These association studies largely included the following: Adhikari et al., 2011; Korte and Farlow, 2013; Gurung et al., 2014; Gao et al., 2016; Pascual et al., 2016; Downie et al., 2018; Phan et al., 2018; Halder et al., 2019; Ruud et al., 2019; Francki et al., 2020; Phan et al., 2021; Lin et al., 2022). After due validation, the markers associated with QTLs and MTAs can be utilized for marker assisted selection (MAS) for resistance breeding.

High-resolution fine-mapping has also been undertaken for sensitivity genes. For this purpose, a high-density genetic linkage map was developed for a chromosome 2D region, which narrowed down the *Snn2* gene to a 4 cM region, thus facilitating the discovery of closely linked molecular markers for breeding and positional cloning of the *Snn2* gene (Zhang et al., 2009). Phenotypic variation (PV) for the disease was 47% for the interaction *Snn2*-*SnTox2*, 20% for *Tsn1*-*SnToxA*, and 66% for both interactions taken together, suggesting the utility of these interactions for breeding (Friesen et al., 2008).

#### Epistatic interactions among fungal NE genes.

Epistatic interactions involving suppression of *SnTox3* by *SnTox1* in the pathogen were also demonstrated (Phan et al., 2016). In this study, the mapping population consisted of 177 double haploid (DH) lines, and an aggressive isolate (Sn15) of the pathogen with genes for three NEs, namely *SnToxA*, *SnTox1* and *SnTox3* and its two deletions (tox1-6 with a deletion for *SnTox1*, and toxa13 with deletion for all the three NE genes) were used; mutant strain toxa13 retained pathogenicity and necrosis-inducing activities in the culture filtrate (Tan et al., 2015); the virulence of this toxa13 on the mapping population at the seedling stage was comparable with that of Sn15.

The following observations also suggested epistatic suppression of *SnTox3* by *SnTox1* and that of *SnToxA* by *SnTox3* in the pathogen: (i) The mapping population segregated for S genes *Snn1* and *Snn3*, since parents of the mapping population differed for these two genes; (ii) When Sn15 was used for infection, *SnToxA*–*Snn1* interaction was most important for SNB development on both seedlings and adult plants, suggesting that *SnToxA* always functioned; no effect of the *SnTox3*–*Snn3* interaction was observed under Sn15 infection. (iii) When tox1-6 strain was used for inoculation, *SnTox3–Snn3* interaction was observed; (iv) When toxa13 strain was used for infection, it unmasked a significant SNB QTL on 2DS, where *Snn2* is located. This QTL was not observed in Sn15 and tox1-6 infections, thus suggesting that *SnToxA* and/or *SnTox3* were epistatic. Additional QTLs responding to SNB sensitivity were detected on 2AS1 and 3AL.

#### Seedling and adult plant field resistance

In each above case, resistance has generally been examined at the seedling stage under controlled conditions using single isolates of the pathogen, but often also examined and compared with those under field conditions (with a mixture of isolates) at the adult plant stage. However, caution should be exercised during evaluation of resistance at the seedling stage for developing resistance under field conditions. Several studies have shown comparable results at the seedling and adult plant stages, when using the same isolate or mix of isolates. However, since the natural infections in the field involve complex pathogen populations with a mixture of isolates, care must be taken to choose representative isolates (see Ruud and Lillemo, 2018 and Peters Haugrud et al., 2022 for recent discussions on this topic).

### Genetics and genomics of *P. nodorum*

Genetic variation among naturally occurring isolates and population genetics of *P. nodorum* has also been examined both at the national level in Sweden (Blixt et al., 2008), Western Australia (Murphy et al., 2000) and Norway (Lin et al., 2020b), and at the global level (Stukenbrock et al., 2005; Stukenbrock et al., 2006). Using RFLPs and SSRs as molecular markers for this purpose, it was shown that in general, *SnToxA* had a relatively higher frequency among *P. nodorum* isolates sampled in different studies (Lin et al., 2020b).

Whole genome sequencing has also been undertaken for *P. nodorum*. The pathogen is haploid with a genome size ranging from 28 Mb to 37 Mb with 23 chromosomes including an accessory chromosome, AC_23_ that is involved in virulence-related functions other than the functions assigned to NEs (Syme et al., 2013; Richards et al., 2018; Syme et al., 2018). A number of isolates, including the following four major isolates were used for genome sequencing: Sn15, Sn4, Sn2000, Sn79-1087. The number of genes in the genome was shown to range from 13,569 for the Sn15 reference genome to 13,294 in Sn79-1087 genome (Hane et al., 2007; Syme et al., 2013; Syme et al., 2016; Richards et al., 2018). Another study involved 197 isolates collected from durum wheat and spring/winter bread wheat from the USA (Richards et al., 2019). These studies together resolved a wide range of structural variations (SVs). A pangenome was also developed using multiple genome sequences (Syme et al., 2018).

### *P. tritici*-*repentis*-wheat pathosystem

TS caused by *P. tritici-repentis* (Died.) Drechs. has been reported from different parts of the world, including Australia, Canada, the USA, Mexico, South America (Argentina and Brazil), Europe, Africa, and Central Asia (Kazakhstan and Tajikistan) (Singh et al., 2008; Ciuffetti et al., 2010). The epidemics for this disease have been reported to cause yield losses of up to ~50% (Rees et al., 1982). The disease is characterized by two distinct and independent symptoms, namely necrosis and chlorosis (**Figures 1B.1, B.2**).

### NE genes, S genes, and interactions

There are three NE genes (*ToxA*, *ToxB* and *ToxC*) and three sensitivity genes (*Tsn1*, *Tsc2* and *Tsc1*) involved in TS. The sensitivity genes and the NEs encoded by three Tox genes are involved in the following three interactions: (i) *Tsn1-*ToxA interaction (this interaction is also known in two other pthosystems, namely wheat-*P. nodorum* and wheat-*B. sorokiniana* pathosystem). (ii) *Tsc2*-ToxB interaction; (iii) *Tsc1*-ToxC interaction (Ciuffetti et al., 2010).

There is strong evidence that *P. tritici*-*repentis* acquired the gene *ToxA* from *P. nodorum* through horizontal gene transfer (Friesen et al., 2006). Among the three NEs, *ToxA* causes necrosis, while *ToxB* causes chlorosis. However, *ToxC*, which also causes chlorosis, is not a protein but a non-ionic, polar, low molecular mass molecule (Effertz et al., 2002).

In the pathogen populations, eight races (races 1 to 8) have been recognized on the basis of the types of susceptibility lesions using six differential wheat genotypes (chlorosis or necrosis) and HSTs/NEs produced (Lamari et al., 2003; Ciuffetti et al., 2010; **Table 5**). Each race produces one or more NEs in a combination, which differs for different races. For instance, races 1, 6, and 7 produce two NEs each (race 1 with ToxA and ToxC, race 6 with ToxB and ToxC and race 7 with ToxA and ToxB). Races 2, 3, and 5 each produce only one NE (race 2 with ToxA, race 3 with ToxC and race 5 with ToxB); race 8 produces all the three NEs, while race 4 is known to produce none (Faris et al., 2013; Guo et al., 2018). In addition to the above three toxins, as many as 38 novel necrosis inducing toxins called ‘triticones’ have also been identified, although only triticone A and triticone B have been purified from Ptr (Rawlinson et al., 2019).

**Table 5:** Reaction of eight races of *P. tritici-repentis* on four bread and two durum wheat differential lines.

	Race (with Toxin) and reaction of six differential genotypes							
Differential genotypes	1	2	3	4	5	6	7	8
	ToxA, C	ToxA	ToxC	None	ToxB	ToxB, C	ToxA, B	ToxA, B, C
Bread wheats								
Glenlea	S1	S1	R	R	R	R	S1	S1
6B662	R	R	R	R	S2	S2	S2	S2
6B365	S	R	S2	R	R	S2	R	S2
Salamouni	R	R	R	R	R	R	R	R
Durum wheats								
Coulter	S1	S1	S1	R	S1	S1	S1	S1
4B1149	R	R	R	R	R	R	R	R

R indicates resistant, S1 indicates susceptible (necrosis), and S2 indicates susceptible (chlorosis).

### Geographic distribution of Ptr NE genes

The distribution of the genes encoding three different NEs (ToxA, ToxB and ToxC) and the wheat genotypes with corresponding S genes involved in interactions differs widely in different parts of the world. Of these, ToxA is the most widely distributed, present in ~80% of the world’s Ptr isolates (Lamari et al., 1998; Ali and Francl, 2002; Friesen et al., 2006).

### Cloned NE genes for TS

Among NE genes, the gene encoding PtrToxA (a 13.2-kDa protein) has been characterized by several independent research groups, and was the first to be cloned (Ballance et al., 1996; Ciuffetti et al., 1997). The gene encoding PtrToxB (6.61 kDa) occurs as multiple copies, and carries a 261-bp open reading frame (ORF) within its sequence (Martinez et al., 2001). PtrToxC has not been fully characterized and purified; the mode of action of this NE is also not known. However, its partial characterization has been done as low molecular weight, non-ionic polar molecule (Effertz et al., 2002).

### Cloned sensitivity genes for TS

*Tsn1* (common for susceptibility to three necrotrophs) was cloned and characterized rather early (**Supplementary Figures S3A, B**; Faris et al., 2010). The other two S genes, *Tsc1* and *Tsc2*, are yet to be cloned and characterized, but markers have been developed for these two other sensitivity genes also (**Supplementary Table S1**). The variety ‘Maris Dove’ was also identified as the historical source of *Tsc2* alleles in the wheat germplasm (Corsi et al., 2020). A minor S QTL was also identified on chromosome 2A (Corsi et al., 2020) in this line. The marker developed in this study can be used for MAS to select insensitive genotypes exhibiting disease resistance (Corsi et al., 2020).

Attempts are also underway to clone *Tsc1* gene. For this purpose, in a recent study, two biparental populations were used leading to the delineation of *Tsc1* candidate gene region to a 1.4 centiMorgan (cM) interval, which spanned 184 kb region on the short arm of chromosome 1A. Mapping of the chlorotic phenotype, development of genetic markers, both for genetic mapping and MAS, and the identification of *Tsc1* candidate genes in this study provide a foundation for map-based cloning of *Tsc1* (Running et al., 2022)

### Genetics of resistance

#### Assessment of sensitivity

In a recent study, 40 Australian spring wheat varieties were examined for sensitivity to ToxA and disease response to a race 1 specific wild-type Ptr isolate carrying *ToxA* and *ToxC* (See et al., 2018). *ToxA* sensitivity was generally associated with disease susceptibility (compatible interaction) but did not always produce symptoms (See et al., 2018). When wild type and *toxA* mutant isolates were used for infection, most *Tsn1* varieties exhibited low disease scores with *toxA* mutants (as expected). However, several varieties exhibited no distinct differences between wild-type and *toxA* mutant (See et al., 2018). This pattern suggested that ToxA is not the sole major cause of TS disease and that the appearance of the disease partly also depends on the background of the host (See et al., 2018). It is thus apparent that ToxA may need additional factors to cause infection (See et al., 2018).

#### R genes for resistance

Resistance genes (R genes or major QTLs) providing resistance against TS have also been identified. Most studies on the genetics of TS are based on bi-parental mapping populations (Faris et al., 2013). Among R genes, *Tsr7* locus was also identified in tetraploid wheat using a set of Langdon durum-wild emmer (*Triticum turgidum* ssp. *dicoccoides*) disomic chromosome substitution lines (Faris et al., 2020). Four user-friendly SNP-based semi-thermal asymmetric reverse PCR (STARP) markers co-segregated with *Tsr7* and should be helpful for MAS (Faris et al., 2020).

#### QTLs for resistance

Several QTLs have been identified, mainly corresponding to the available S-genes (Liu et al., 2020a). The QTL studies resulted in the identification of as many as >160 QTLs; a number of these QTLs explained >20% phenotypic variation (**Supplementary Table S2**). A meta-QTL analysis was also conducted, leading to the identification of 19 meta-QTLs derived from the results of 104 QTL studies (Liu et al., 2020a; Liu et al., 2020b). Three race nonspecific meta-QTLs were also identified, one each on chromosomes 2A, 3B and 5A. These three meta-QTLs had large phenotypic effects, each responsible for resistance to multiple races infecting bread and durum wheat races, thus suggesting their utility for marker-assisted selection (MAS).

#### GWAS for resistance

A number of GWA studies involving the identification of MTAs for TS resistance are also available (Patel et al., 2013; Kollers et al., 2014; Liu et al., 2015; Juliana et al., 2018; Dinglasan et al., 2019; Galagedara et al., 2020; Kokhmetova et al., 2020; Muqaddasi et al., 2021). These studies led to the identification of >240 MTAs, although many of these could be false positives (**Supplementary Table S3**). Candidate genes were found for 16 out of 19 meta-QTLs; the candidate genes for each meta-QTL ranged from 2 to 85, with most of them being on chromosome 2B. Many of these potential genes encoded NBS- and/or LRR-like proteins and were found near the *Tsc2* S gene (Liu et al., 2020a). However, none of these candidate genes could be actual *Tsc2* gene, because the genomic sequence used to identify candidate genes belonged to Chinese spring (CS) wheat, insensitive to Ptr ToxB. (Liu et al., 2020a).

#### Genetic studies at seedling and adult plant stage under field conditions

The genetics of resistance against TS in wheat has been examined both at the seeding and adult plant stages. For instance, in a study of ~300 accessions from Vavilov collection, seedling but not adult plant disease response corresponded with ToxA sensitivity; ToxA-sensitive accessions that were susceptible at the seedling stage, carried adult-plant resistance (APR) (Dinglasan et al., 2017). In a follow-up GWAS, they identified 11 QTL, of which were associated as follows: 5 with seedling resistance, 3 with all-stage resistance, and 3 with APR. Interestingly, the novel APR QTL was effective even in the presence of host sensitivity gene *Tsn1* (Dinglasan et al., 2019).

### Genetics and genomics of *P. tritici*-*repentis*

The genetic studies on isolates of *P. tritici-repentis* from different parts of the world have also been conducted using molecular markers. It was shown in several studies including one from Oklahoma in USA that race 1 was the predominant race in most regions (Ali and Francl, 2003; Friesen et al., 2005; Kader et al., 2022).

The Ptr genome is 40.9 Mb in size and has already been fully sequenced (Moolhuijzen et al., 2018). As much as 98% of the genome has been mapped on 10 or more chromosomes, carrying 13,797 annotated genes (Moolhuijzen et al., 2018). The *Ptr ToxA* is a single copy gene (Ballance et al., 1989; Tomas et al., 1990; Tuori et al., 1995; Ballance et al., 1996; Zhang et al., 1997; Ballance et al., 1998), producing a 19.7 kD protein precursor (Ballance et al., 1996; Ciuffetti et al., 1997). *Ptr ToxB*, on the other hand, is a multi-copy gene (1-3 Kb in length; Martinez et al., 2004) that encodes a 6.6 kDa host selective toxin (Strelkov et al., 1999; Martinez et al., 2004). When the genome of a race 5 Tox-B isolate was sequenced, ten identical *ToxB* gene copies were identified (Moolhuijzen et al., 2020). Multiple *ToxB* gene loci on chromosome 10 were separated by 31-66 kb long segments and exhibited an alternating pattern involving forward and reverse DNA strands. Also, the gene is flanked by transposable elements (Moolhuijzen et al., 2020; **Supplementary Figure S4**).

### *B. sorokiniana*-wheat pathosystem

*B. sorokiniana* is a hemi-biotroph, which causes several important wheat diseases, namely SB (**Figure 1C**), common root rot (CRR), black point and crown rot; these diseases are responsible for significant yield losses in several parts of the world (Gupta et al., 2018a; Gupta et al., 2018b). The correlations between these diseases in wheat seem to be poor and mechanisms for resistance against these diseases seem to differ (Conner, 1990; Al-Sadi, 2021). However, we will restrict our discussion to only spot blotch.

The gene *ToxA* initially reported in *P. nodorum* and *P. tritici-repentis* (Tuori et al., 1995; Friesen et al., 2006), has also been identified in some *B. sorokiniana* isolates from USA (South Central Texas), Australia, India, and Mexico. It was also shown that a *B. sorokiniana* isolate harbouring *ToxA* (dominant alleles of S gene) is more virulent on wheat lines carrying the S gene *Tsn1* (McDonald et al., 2018; Navathe et al., 2020).

### NE gene, S gene, and the interaction

A solitary sensitivity gene (*Tsn1*) in the host (wheat) and the corresponding NE gene (*ToxA*) in the pathogen are known for wheat-SB pathosystem. The presence of the *Tsn1* gene is generally but not always associated with susceptibility to the pathogen carrying the *ToxA* gene, as shown in the material surveyed in Australia and India (McDonald et al., 2018; Navathe et al., 2020). It was reported that sometimes an isolate lacking *ToxA* is still highly virulent on cultivars from Australia and India lacking *Tsn1*. In contrast, a cultivar containing *Tsn1* can still be resistant to isolates carrying the *ToxA* gene. These results suggest that there are additional factors in the wheat genome, which control resistance; these factors may include R genes and QTLs controlling resistance to SB (Navathe et al., 2020).

### *BsToxA* differs from *SnToxA* and *PtrToxA*

The gene *BsToxA* is embedded in the 12-kb AT-rich region of the pathogen genome (McDonald et al., 2013; McDonald et al., 2018). Decay near the gene edges has been reported and is attributed to repeat-induced polymorphism (RIP) (**Supplementary Figure S5**; McDonald et al., 2018). Small indels have also been reported in the gene’s promoter region; the size of indel in *BsToxA* (148 bp) differs from that, in *SnToxA* (43 bp) and *PtrToxA* (238 bp) (McDonald et al., 2012; McDonald et al., 2018; also see **Supplementary Figure S5**). The haplotype organization of *ToxA* genes in three pathogens (*BsToxA*, *PtrToxA* and *SnToxA*) also differed (McDonald et al., 2018). The frequencies of pathogen isolates carrying *BsToxA* and the wheat genotypes carrying *Tsn1* also differ in different parts of the world (Friesen et al., 2018; McDonald et al., 2018; Navathe et al., 2020; Wu et al., 2021).

Sensitivity-related QTLs against *B. sorokiniana* have also been identified in barley, where recessive alleles of *Rcs5* and *Rcs6/Scs2* provided resistance to SB (Gupta et al., 2018a; Leng et al., 2018). Two additional QTLs (*QSbs-1H-P1* and *QSbs-7H-P1h*) for susceptibility to SB were also identified in barley; of these two QTLs, *QSbs-7H-P1* mapped to the same region as the *Rcs5* gene, but *QSbs-1H-P1* was a novel QTL later reported by Leng et al. (2020).

### Genetics of resistance

#### R genes

Resistance to SB is mainly associated with one or more of the four major R genes (*Sb1-Sb4*) that were identified using classical methods of genetics for Mendelian traits. The genetics of resistance to spot blotch has also been studied, taking the disease as a quantitative trait (reviewed by Gupta et al., 2018a).

#### QTLs/MTAs

Using interval mapping, ~70 QTLs were identified, which included 14 major QTLs with PVE >20% (**Supplementary Table S2**). Some of the QTLs were inherited in a Mendelian manner and overlapped the known major Sb genes (Lillemo et al., 2013; Kumar et al., 2015; Lu et al., 2016; Zhang et al., 2020). Such QTLs were later designated as *Sb1* (*QSb.bhu-7DS*) and *Sb2* (*QSb.bhu-5BL*) in two independent studies (Lillemo et al., 2013; Kumar et al., 2015). *Sb1* gene is also associated with *Lr34*, an important gene for leaf rust resistance in wheat (Lillemo et al., 2013). Using GWAS also, ~80 MTAs were identified (Gupta et al., 2018a; Chand et al., 2021; **Supplementary Table S3**). These interval mapping studies and GWAS were generally conducted only at the adult plant stages.

#### Resistance at seedling and adult stages

Resistance against SB in wheat has generally been examined only at the adult plant stage. Only in a recent study, resistance at seedling and adult plants stages and its association with biochemical profiling was examined (Mahapatra et al., 2021).

### Phylogeography and genomics of *B. sorokiniana*

The phylogeographic pattern of *B. sorokiniana* isolates was examined in a recent study involving 254 isolates from different parts of the world with the goal to elucidate the demographic history. In this study, 162 ITS, 18 GAPDH and 74 TEF-1a gene sequences from *B. sorokiniana* obtained from GenBank were utilized and 40 haplotypes were identified (Sharma et al., 2022). It was inferred that human-mediated dispersal perhaps played a major role in shaping the distribution of *B. sorokiniana*.

Genomic studies of *B. sorokiniana* include generation of a draft genome sequence followed by a refined genome sequence of an Indian isolate, namely *B. sorokiniana* strain BS_112. These genome sequemces were reported in two independent publications by Aggarwal et al. (2019), Aggarwal et al. (2022) from Indian Council of Agriculture Research-Indian Agriculture Research Institute (ICAR-IARI) in India. The genome size was estimated to be 35.64 Mb, with an average G/C content of 50.20%. A total of 10,460 genes were predicted with an average gene density of 250 to 300 genes/Mb, which covers around 98% of predicted genes. The lengths of genes ranged from 50 bp to 8,506 bp with an average length of 435 to 545 bp per gene.

### *Z. tritici-*wheat pathosystem

*Z. tritici* (syn. *Septoria tritici*, *Mycosphaerella graminicola*) is an important apoplastic fungal pathogen causing STB, which is responsible for major yield losses, sometimes approaching 50% under severe epidemic conditions (Eyal, 1973; Eyal et al., 1987). The fungus has been shown to be a hemibiotroph (Fones and Gurr, 2015), with the following two main phases: (i) the initial symptomless biotrophic latent phase (typically lasting for about 10 to 12 days), during which the hyphae enter the leaves through stomata and colonize the leaf tissues (Kema et al., 1996), and (ii) the later necrotrophic phase (Hehir et al., 2018), when the host tissue begins to die, and the fungus feeds on dead tissue (Keon et al., 2007).

In wheat-*Z. tritici* pathosystem, the host secretes β-1,3-glucanase into the apoplast, which cleaves β-1,3-glucan in the pathogen’s cell wall and prevents colonization of the pathogen (Shetty et al., 2009). The major R genes and QTLs for the STB disease have been listed by Brown et al. (2015) and are also available on the Komugi database (https://shigen.nig.ac.jp/wheat/komugi/).

### NEs, ZtNIP1/2, MgNLP and ZtSSPs

**Figure 3:**
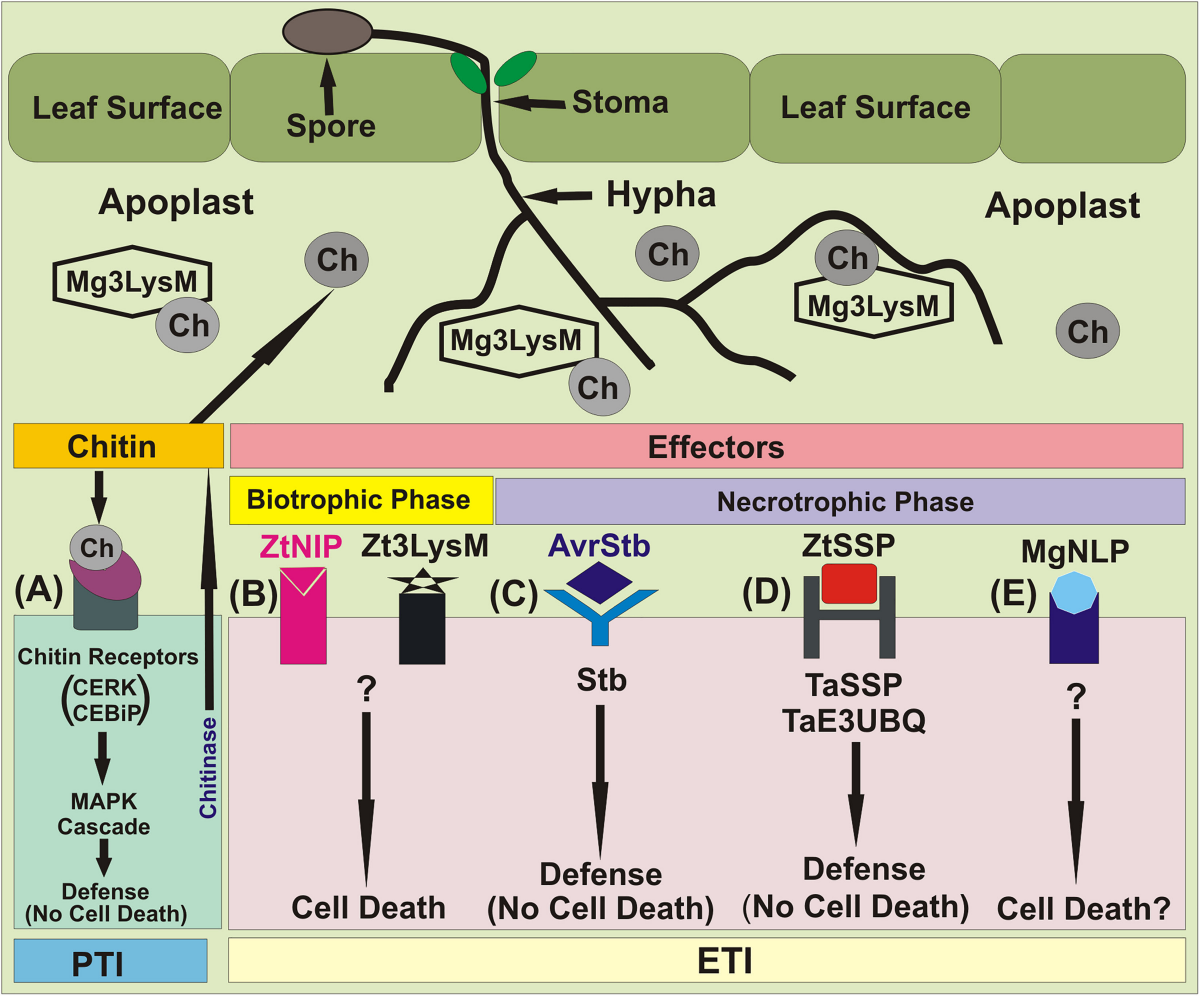
Major molecular events during *Z. tritici*–wheat interactions. **(A)** Fungal PAMP chitin is recognized by the host receptors Chitin Elicitor Binding Protein (CEBiP) and Chitin Elicitor Receptor Kinase 1 (CERK1), triggering MAP kinase cascades and immune activation. Multi-functional LysM-domain containing effector Mg3LysM scavenges chitin to suppress immunity and protects fungal hyphae from wheat chitinases. **(B)** ’Necrotrophic’ effectors (NEs), Necrosis-Inducing Protein 1/2 (ZtNIP1/2) and and LysM effector (Zt3LysM) induce host cell death. **(C)** *Stb* gene-specified resistance, presumably triggered following recognition of cognate fungal effectors (AvrStb) secreted into the apoplast. This results in arrest of pathogen growth *via* an unknown mechanism that does not involve HR. **(D)** The NEP1-like effector protein MgNLP (unknown *Z. triticini* effector, predicted by bioinformatics analysis) has an unknown function(s) during wheat infection, but triggers cell death in dicots. **(E)** Small secreted proteins (ZtSSPs) of *Z. tritici*, which act as an effector. The TaE3UBQ synthesize in the wheat and interacts with the ZtSSPs resulting inhibition of the growth of *Z. tritici* pathogen.

Hundreds of *Z. tritici* candidate effector genes have been identified through comparative genomics and transcriptomics (Gohari, 2015; Rudd et al., 2015; Kettles et al., 2017; Palma-Guerrero et al., 2017; Plissonneau et al., 2018). Three well characterized LysM effector genes (NE genes), namely *Mg3LysM*, *Mg1LysM* and *Mgx1LysM* have also been identified (Marshall et al., 2011; Tian et al., 2021). Initially, only two LysM effectors, namely Mg1LysM and Mg3LysM, were known. Among these two effectors, Mg3LysM, but not Mg1LysM, was shown to suppress the response of the host immune system at the level of pattern triggered immunity (PTI) (**Figure 3**).

The host’s immune system involves synthesis of chitinases, which destroy fungal cell wall chitin that causes virulence. A third LysM gene, which was initially believed to be a pseudogene, was later shown to encode a LysM effector, named Mgx1LysM, also named Zt3LysM (Zhang, 2022). Later Zt3LysM effector was also shown to contribute to *Z. tritici* virulence, and to protect fungal hyphae against hydrolysis by chitinases of the host. All three LysM effectors display partial functional redundancy (Tian et al., 2021).

In addition to three LysMs as above*, Z. tritici* also secretes many rapidly evolving, small secreted proteins (ZtSSPs), which function as effectors and help the pathogen to colonize plant tissue. In a recent study, while working with the pathogen’s SSPs, ZtSSP2 was found to express throughout *Z. tritici* infection phase in wheat, with the highest levels observed early during infection. A study of the interaction between ZtSSP2 and wheat E3 ubiquitin ligase (TaE3UBQ) further confirmed that down-regulation of the gene encoding TaE3UBQ using virus-induced gene silencing increased the susceptibility of wheat to STB, suggesting that ZtSSPs also function as effectors. These results also suggested that the wheat TaE3UBQ plays a role in plant immunity and helps the host to achieve defense against *Z. tritici*.

### No S gene for STB

Despite major search and extensive studies in wheat–*Z. tritici* pathosystem, no S gene for compatible interaction between wheat and *Z. tritici* has been discovered so far. Earlier, till a few years ago, the same was true for *B. sorokiniana*, till McDonald et al. (2018) discovered the occurrence of *Tsn1* gene interacting with ToxA of the pathogen *B. sorokiniana*. It is, therefore, possible that a S gene for STB may also be discovered in the future, although it seems unlikely in view of the relatively extensive studies already conducted on wheat–*Z. tritici* pathosystem.

### Genetics of resistance

#### TaSSPs and TaE3UBQ genes for resistance

As in the case of many other pathogens, in addition to LysM effectors, *Z. tritici* also secretes many SSPs, which can block plant defense and permit pathogens to colonize plant tissue (Zhou et al., 2020). It has also been shown that there are also independent TaSSP loci in the host associated with resistance to STB at seedling as well as at the adult plant stages.

In a recent study involving wheat genomics, an SSP-discovery pipeline was developed and 6,998 TaSSPs (each with <250 AA) were identified, which included 141 *Z. tritici* – responsive TaSSPs. A subset of these TaSSPs also had a functional signal peptide, which could interact with *Z. tritici* SSPs. It was also shown that the synthesis of TaSSPs was induced during pathogen attack. In a wheat cultivar named Stigg, two of these TaSSPs, namely *TaSSP6* and *TaSSP7*, when silenced using virus induced gene silencing (VIGS) led to susceptibility, thus confirming the role of TaSSPs in defense against *Z. tritici* (Zhou et al., 2020). ZtSSP2 was also shown to interact with wheat E3 ubiquitin ligase (TaE3UBQ) thus confirming that down-regulation of this wheat E3 ligase using VIGS increased the susceptibility of wheat to STB. These results suggested that perhaps TaE3UBQ gene also plays a role in providing resistance against *Z. tritici* (Karki et al., 2021).

#### R (Stb) genes for resistance

In the germplasm of wheat, which included landraces, wild wheat species, and synthetic hexaploid wheat, 22 major R genes (named Stb genes) have been identified and characterised (Saintenac et al., 2021; **Supplementary Table S1**). Most of these Stb genes are genotype-specific, each providing short-term resistance against only a few *Z. tritici* isolates. Among these 22 Stb genes, *Stb6* and *Stb16q* are the two major genes, which exhibit GFG relationship, each providing broad spectrum resistance against a majority of *Z. tritici* isolates; both these genes have been cloned and characterized. *Stb6* gene was shown to code for a wall-associated kinase (WAK), which represents a subfamily of receptor-like kinases (RLKs), which are involved in GFG of resistance against isolates carrying the matching *AvrStb6* gene (Saintenac et al., 2018). Similarly, the gene *Stb16q* encodes a plasma membrane cysteine-rich receptor like kinase (CRK, also a member of the RLK family of kinases). There is evidence that this gene (*Stb16q)* is derived from *Ae. tauschii via* synthetic wheat and has now become a part of wheat genome. This origin of *Stb16q*, also suggested the importance of wild relatives of wheat in the improvement of disease resistance in wheat cultivars (Saintenac et al., 2021).

#### AvrStb6–Stb6 interaction provides early defense

An avirulence locus called *AvrStb6* was also identified using diverse *Z. tritici* populations. The corresponding wheat locus *TaStb6* was also shown to be associated with qualitative resistance on multiple wheat cultivars (Kema et al., 2000; Brading et al., 2002; Chartrain et al., 2005c). The *AvrStb6* gene confers a GFG interaction with the *TaStb6* gene of wheat (Zhong et al., 2017; Kema et al., 2018). No direct interaction between the *AvrStb6* and *Stb6* proteins has been reported, and no typical hypersensitive resistance (HR) was noticed during the resistance response, indicating that programmed cell death (PCD) was not the mode of resistance in this case (Friesen and Faris, 2021).

*Stb7* is another important R gene, which is recognised by the *Avr3D1* gene of the pathogen, triggering a strong defence response, but without preventing pathogen infection. In an important study, *Avr3D1* gene was found to be present in all 132 strains of *Z. tritici* that were used in the study and provided a strong fitness advantage (Meile et al., 2018). Allelic differences at the locus of *Avr3D1* are responsible for maintaining the gene but still evading recognition by the host harbouring *Stb7* (Friesen and Faris, 2021). The *Avr3D1* gene is upregulated during biotrophic phase but downregulated in the necrotrophic phase, thus permitting early colonisation (Meile et al., 2018).

#### QTLs/GWAS for resistance

Quantitative resistance against STB is controlled by QTLs, each with a small to moderate effect, thus providing relatively durable resistance. Relative to Stb genes, these QTLs have weak specificity. According to a review by Brown et al. (2015), till 2015, 89 genomic regions carrying quantitative trait loci (QTLs) or meta-QTLs were known. Some of these QTLs have also been mapped at or near Stb genes like *Stb6* and *Stm16q*, which are each present in many genotypes.

Many interval mapping studies involving identification of QTLs have already been conducted (Adhikari et al., 2015; Stadlmeier et al., 2019; Tamburic-Ilincic and Rosa, 2019; Riaz et al., 2020). These studies suggested that host-pathogen interaction is complex and can-not be explained by simple R–Avr interactions. Additive epistatic interactions were also reported for more minor and significant qualitative effects that govern virulence for *Z. tritici* (Jones and Dangl, 2006; Meile et al., 2018; Stewart et al., 2018). The role of necrosis inducing protein 1 (ZtNIP1) of *Z. tritici* has been shown to trigger PCD. This protein is also expressed during 8 and 12 dpi and correlates with necrotic phase symptoms (Ben M’Barek et al., 2015). The ‘Necrosis and Ethylene-Inducing Peptide 1’ (NEP1) is also involved in causing necrosis (Kettles and Kanyuka, 2016).

A number of GWA studies for identification of MTAs (QTLs) for resistance against STB have also been conducted. The results of four such studies are summarized in **Supplementary Table S3**.

#### Seedling vs adult plant resistance

Majority of the 22 Stb genes contribute to STB resistance independently of the plant growth stage, although resistance can also be effective only in seedlings or only in adult plants. In a recent QTL mapping study on seedling and adult plant resistance, Piaskowska et al. (2021) reviewed the literature on this subject and reported identification of a new QTL (*QStb.ihar-2B.4*) for resistance at the seedling stage (PV upto 70.0%), thus proving its utility in breeding programs. In another recent study, Yang et al. (2022) reported identification of new QTLs for seedling resistance and APR, also described as multi-stage resistance (MSR) QTLs. Two of these new QTLs included *QStb.wai.6A.2* for APR and *QStb.wai.7A.2* for MSR.

### Genetics and genomics of *Z. tritici*

Population genetics of *Z. tritici* from northern France, Iran, UK, Canada, and Ethiopia was also examined using molecular markers (generally SSR markers). Significant genetic diversity was reported in all these studies; the latest of these studies by Mekonnen et al. (2020) described an average of 2.5 alleles per SSR locus, although in some earlier reports a higher level of diversity was also reported.

The genome of *Z. tritici* carries 21 chromosomes, which include 13 core chromosomes and 8 dispensable/accessory chromosomes (Croll and McDonald, 2012). The genome size varies from 32 to 40 Mb, and the pangenome carries a core set of 9,149 genes (McDonald and Martinez, 1991; Mehrabi et al., 2007; Romdhane, 2011). Genome sequences of *Z. tritici* indicated the following important features (Stukenbrock et al., 2010); (i) The essential and dispensable chromosomes evolved differently and independently, the former being syntenic, while the latter carrying many structural rearrangements. (ii) The average synonymous substitution rate in dispensable chromosomes is considerably lower than in essential chromosomes, whereas the average non-synonymous substitution rate is three times higher. (iii) As many as 43 candidate genes showed evidence of positive selection, one of these genes encoding a potential pathogen effector protein.

## Similarities and differences among four pathosystems

The four pathosystems involved in four leaf spot diseases of wheat discussed above have several similarities and differences. Among similarities, the pathogens involved in these four pathosystems are all fungal pathogens belonging to the phylum Ascomycota, and all are either necrotrophs or hemibiotrophs, there being a thin cryptic line of distinction between necrotrophs and hemibiotrophs (Rajarammohan, 2021). The hemibiotrophic nature of *Z. tritici* has also been questioned (Sanchez-Vallet et al., 2015). Following are some other similarities: (i) occurrence of both GFG relationship involving R genes of the host and Avr genes of the pathogen and IGFG relationship, involving sensitivity (Tsn/Snn) genes of the host and NE genes of the pathogen. In this respect, the pathosystem involved in STB is the only exception. The pathogens also exhibit similar modes of reproduction involving asexual reproduction through conidia and sexual reproduction involving a mating system and producing ascospores. In this respect, *B. sorokiniana* is an exception being an anamoporph (its sexual form being teleomorph, described as *Cochliobolus sativus*). Another major difference includes homothallic nature of sexual forms: *P. nodorum* and *P. tritici-repentis* are homothallic, as against heterothallic nature of *B. sorokiniana* and *Z. tritici*. The availability of sexual reproduction also has a bearing on the diversity of the pathogen, the frequent sexual reproduction leading to higher level of diversity. Other differences include the number of known sensitivity (S) genes, R genes and QTL/QRL in the host and NE/Avr genes in the pathogen, there being nine S genes in the host and eight NE genes in the pathogen for SNB involved in nine interactions, three S genes and three NE genes for TS, only one S gene (*Tsn1*) and one NE gene (*ToxA*) for SB, and there being no known S gene for STB. The pathosystem involving STB also differs for the occurrence of a relatively large number of R genes (22 Stb genes) in the host and ZtSSP genes in the pathogen for virulence and the TaSSP genes for resistance/defence in the host.

Based on the occurrence of R/SSP genes and QTLs/QRLs for resistance and S genes for susceptibility in the wheat genome and the corresponding Avr/NE/SSP genes in the pathogen, one can perhaps try to study the interactions among these genes and plan strategies for developing resistance involving each of the four pathosystems. One such project for SB has already been planned by the authors of this review.

## Breeding strategies

### S genes, R genes and QTLs for resistance

The sensitivity (S) genes in bread wheat as the host are the most important source of susceptibility involving compatible interaction between the host and the pathogen, so that apparently the loss of these genes or use of their recessive alleles or mutant alleles should be the major sources of resistance. The interactions between S genes of the host and the NE genes of the pathogens for four diseases are summarised earlier in this review. All these cases represent examples of IGFG, where the pathogen can-not cause the disease, unless the host carries the corresponding S gene, which is recognized by the pathogen-derived effector.

There are examples, where a loss-of function mutations in S genes may either occur naturally, or else may be induced through mutagenesis. The most important example of such a loss of function mutations is the loss-of function of the *Mlo* gene in barley and many important cereals (including wheat and rice), vegetables (tomato, pepper, cucumber, and melon), legumes (peas and lentils), fruit trees and shrubs (apples, grapevines, peaches, and strawberries), and flowers (petunia and roses) providing resistance to powdery mildew disease. It is still unknown whether all these Mlo homologs can act as susceptibility genes in their respective hosts (for details of References, see Phd Thesis of Pavan, S. 2011). However, the susceptibility gene *Mlo* differs from the S genes like *Tsn1* involved in diseases like SNB and TS and SB in wheat, although this difference is not apparent. Examples of actual use of recessive alleles or mutants of S genes for breeding wheat cultivars with resistance against a necrotroph are limited. In a recent review involving evaluation of the role of NE-S genes in development of resistant cultivars, it was shown that most of the wheat cultivars in Eastern USA, carried durable quantitative SNB resistance and that *Snn–*NE interactions had very little role in providing resistance (Cowger et al., 2020).

If S genes did not play any major role in resistance breeding, it is apparent that either classical R genes or QTLs must be the source of resistance as shown in several studies cited earlier in this review. These resistant cultivars apparently resulted by an unconscious selection of specific R genes or QTLs. Since R genes and QTLs are now known for each of the four necrotrophs under review, one may plan a strategy, where specific R genes or QTLs may be used for developing resistant cultivars. This should be possible because molecular markers associated with these R genes and QTLs are now available.

QTLs using interval mapping and MTAs associated with QTLs using association mapping have also been discovered for almost all nerotrophs and hemi-biotrophs. A list of known S genes, R genes and QTLs for the four diseases under review are listed in **Supplementary Tables S1–S3** suggesting that all the three systems operate in necrotrophs as well as hemibiotrophs and can be exploited for imparting resistance against the corresponding diseases.

**Figure 4:**
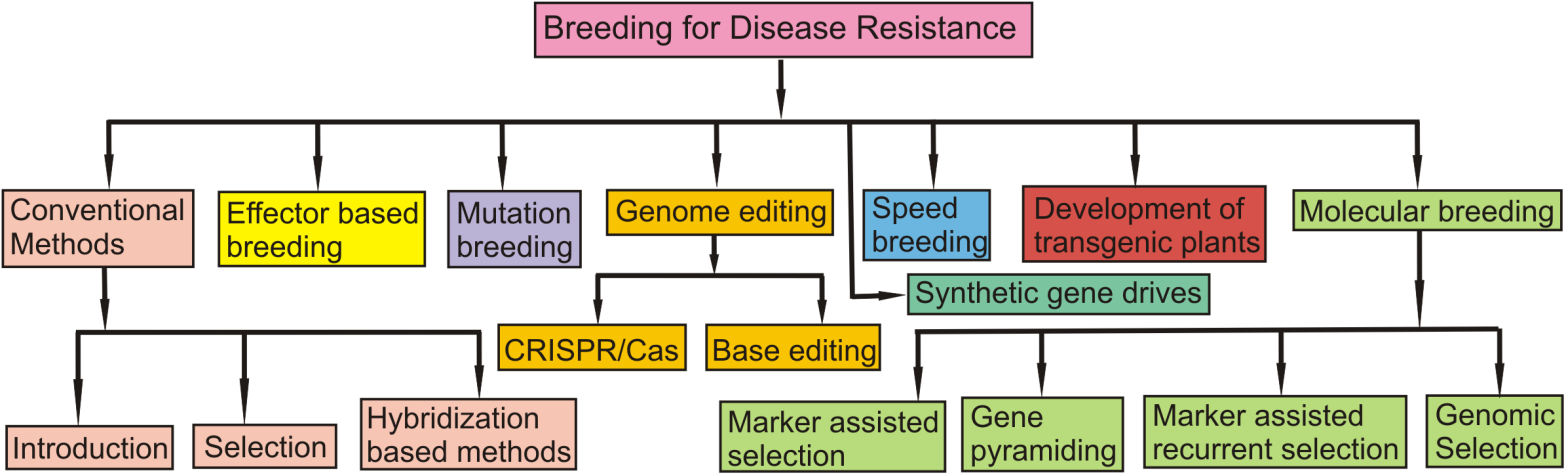
A schematic figure which shows a holistic view of the management of four foliar diseases through genetic tools.

The distribution of different S genes in wheat cultivars and that of NE genes in the isolates of the pathogen *P. nodorum* causing SNB has also been examined in multiple locations in different parts of the world. The relative frequencies of S genes in wheat cultivars and those of races with three different Tox genes in the pathogen are summarized in **Table 3**. In some cases, races could be classified based on NE constitution. For instance, eight races for the TS pathogen have been characterized based on their NE constitution (**Table 5**). A holistic view of the management of four foliar diseases through genetic tools is also given in **Figure 4**.

### Identification of effectors and effector-assisted breeding

It is widely known now that effectors that are produced by a variety of pathogens, targeing host cells causing diseases. Development of tools for identification and utilization of these effectors has consequently been recognized as a new resistance breeding strategy. During the last two decades, hundreds of these effectors have already been identified and genome-wide catalogues of effectors have become available, such that effectoromics has emerged as a new area for research. Effector-assisted breeding has also been shown to be successful for some crops (for a review, see Vleeshouwers and Oliver, 2014). The question therefore is, whether or not the disease resistance of new cultivars can be accurately predicted from the response to effectors of the input germplasm. Genetic analysis of the response to purified effectors allowed the identification of several wheat genetic loci that correspond to regions conferring susceptibility to the disease.

Methods are also available for the identification of effectors in the secretome using conserved domains, which are common among effector families. One such example is the presence of RXLR (Arg-X-Leu-Arg) motif. Other effector motifs, located in the C-terminal and N-terminal regions, include CRN, LysM, RGD, DELD, EAR, RYWT, Y/F/WXC or CFEM. Rapid identification of effectors using RXLR motif allowed development of a catalogue of effectors for *Phytophthora infestans*, which later enabled identification of R genes in potato, Arabidopsis, and lettuce. More recently, WAxR motif has been found in different effectors in races of *Puccinia striiformis* (yellow rust) and other rust races through screening of secretomes. The tool EffectorP 3.0 has also been utilized for the identification of effectors (Sperschneider and Dodds, 2022).

A high-throughput screening procedure for evaluating wheat genotypes through the infiltration of effectors like ToxA into wheat leaves has also been developed and used in Australia (Tan et al., 2014; Vleeshouwers and Oliver, 2014). This method helped in the quick elimination of *Tsn1* from commercial cultivars. As a result, the area sown with ToxA sensitive cultivars was reduced from 30.4% to 16.9% during the three-year period following the use of this screening system (Vleeshouwers and Oliver, 2014; Cockram et al., 2015). However, the application of this method to other pathogens could be more complicated because all three homoeologues of the susceptibility/sensitivity gene must be eliminated to achieve resistance. In addition, undiscovered effectors that can differ between different regional populations may occur in the pathogen. Notwithstanding this, effector-assisted selection can be an effective way for determining weak and environment-dependent QTL (Vleeshouwers and Oliver, 2014; Downie et al., 2018). Moreover, this method enables to dissect components of quantitative resistance, develop diagnostic markers and fine-map susceptibility genes. Such markers can be converted into Kompetitive Allele-Specific PCR (KASP) markers for the rapid selection of desirable alleles. Thus, using effector-assisted selection for developing diagnostic markers can increase the pace of resistance breeding in wheat against necrotrophs (Cockram et al., 2015; Downie et al., 2018).

Necrotrophic effectors from *P. nodorum* (Pn) and toxic proteins from *Z. tritici* have also been utilized to detect R genes/QTLs in wheat (Lebrun et al., 2016). These effector/toxin proteins were first produced in yeast and purified proteins were obtained. These proteins were then delivered to wheat leaves through syringe infiltration. Disease symptoms were then scored after a few days. Screening of 220 elite French wheat cultivars with Pn ToxA 1 and Pn ToxaA3 allowed the identification of cultivars that were insensitive to the three necrotrophic effectors, and only a few were sensitive, suggesting that breeding for field resistance against Pn during 1960-1980 led to the accumulation of insensitive alleles (recessive alleles). The insensitive genotypes can be tested against Pn isolates producing Tox1 and Tox3 effectors, and insensitivity loci can be mapped using GWAS and associated markers can be identified. This type of work will facilitate resistance breeding through MAS (For a review, see Li Q. et al., 2021).

In case of TS in Western Australia also, the elimination of a single effector, PtrToxA and the corresponding S gene *Tsn1*, has a dominating impact in breeding for disease resistance. The availability of ToxA to breeders has had a major impact on cultivar choice and breeding strategies. For *P. nodorum*, three effectors (SnToxA, SnTox1, and SnTox3) have been well characterized. Unlike TS, no one effector has a dominating role. Genetic analysis of various mapping populations and pathogen isolates has shown that different effectors have varying impact, and that epistatic interactions also occur. As a result of these factors, the deployment of these effectors for SNB resistance breeding is complex.

Tan et al. (2015) deleted genes encoding the three effectors in a strain of *P. nodorum* and measured effector activity and disease potential of a triple knockout mutant. The culture filtrate caused necrosis in several cultivars and the strain caused disease, albeit the overall levels are less than in the wild type. Modeling of the field disease resistance scores of cultivars from their reactions to the microbially expressed effectors SnToxA, SnTox1, and SnTox3 is significantly improved by including the response to the triple knockout mutant culture filtrate. This indicated that an additional one or more effectors are secreted into the culture filtrate. It was concluded that the *in vitro*-secreted necrotrophic effectors explain a very large part of the disease response of wheat germplasm and that this method of resistance breeding promises to reduce further the impact of these globally significant diseases. Thus, elimination of genotypes carrying *Tsn1* gene, inducing knockout mutants and introgression of QRLs are the three approaches that can be used for resistance breeding.

### QTLs for resistance breeding

For diseases like SNB and TS, generally R genes are not known for breeding. In these cases, resistance is mainly quantitative in nature, governed by QTLs. Most QTLs had a limited effect that was hard to measure precisely and varied significantly from site to site and season to season.

In the late 1920s, A. E. Watkins collected ~7000 landrace cultivars (LCs) of bread wheat (*Triticum aestivum* L.) from 32 different countries around the world. Among these LCs, 826 LCs were viable and could be a valuable source of superior/favorable alleles to enhance disease resistance in wheat. Halder et al. (2019) used a core set of 121 LCs carrying the entire genetic diversity of Watkins collection, and evaluated them for identification of novel sources of resistance against SNB, TS and Fusarium Head Blight (FHB). Response for the three diseases, however, differed in 121 LCs, most of them being either moderately susceptible or susceptible to TS Ptr race 1 (84%) and FHB (96%), whereas a large number of LCs were either resistant or moderately resistant against TS Ptr race 5 (95%) and SNB (54%). Thirteen LCs were identified, which could be a valuable source for multiple resistance to TS Ptr races 1 and 5, and SNB, and another five LCs could be a potential source for FHB resistance.

GWA studies using 118 LCs were also carried out using disease phenotyping score and genotyping data using 8,807 SNPs data leading to identification of 30 significant MTAs (Halder et al., 2019). Ten, five, and five genomic regions were also found to be associated with resistance to TS Ptr race 1, race 5, and SNB, respectively in this study. In addition to *Tsn1*, several novel genomic regions were also identified, which included the following: (i) *Q.Ts1.sdsu-4BS* and *Q.Ts1.sdsu5BS* (TS Ptr race 1) and (ii) *Q.Ts5.sdsu-1BL*, *Q.Ts5.sdsu-2DL*, *Q.Ts5.sdsu-3AL*, and *Q.Ts5.sdsu-6BL* (TS Ptr race 5). These results indicated that these putative genomic regions contain several genes that play an important role in plant defence mechanisms. It was concluded that SNP markers linked to QTLs for SNB and TS resistance along with LCs harboring multiple disease resistance could be useful for future wheat breeding.

### From QTLs to genes (R-Genes on 1BS and 5BL)

QTLs controlling response to SNB were initially identified on chromosomes 1BS and 5BL (although QTLs on other chromosomes are also known now). Li D. et al. (2021) conducted a study involving alignment of the genetic map with QTLs on 1BS and 5BS with the reference sequence of wheat. This allowed the identification of R-genes associated with SNB response, although correspondence of R genes with QTL was not shown. Alignment of QTL intervals allowed identification of significant genome rearrangements on 1BS between parents of the DH population (EGA Blanco, Millewa) and the reference sequence of Chinese Spring with subtle rearrangements on 5BL. Nevertheless, annotation of genomic intervals in the reference sequence allowed identification and mapping of 13 R-genes on 1BS and 12 R-genes on 5BL. R-genes discriminated co-located QTL on 1BS into following two distinct but linked loci, both associated with SNB resistance but in one environment only: (i) NRC1a and TFIID mapped in one QTL on 1BS, whereas (ii) RGA and *Snn1* mapped to QTL on 1BS. Similarly, *Tsn1* and *WK35* were mapped in one QTL on 5BL, with NETWORKED 1A and resistance gene analogs (RGA) genes mapped to the linked QTL interval. This study provided new insights on possible biochemical, cellular, and molecular mechanisms responding to SNB infection in different environments and also addressed limitations of using the reference sequence to identify the full complement of functional R-genes in modern varieties.

### Multiple disease resistance

Since genotypes with multiple resistance and association among more than one disease have now been reported, it is also possible to plan a breeding programme for transfer of resistance for more than one disease using a single donor, as recommended by Gurung et al. (2014).

### Possible molecular breeding approaches

As shown above, resistance against leaf spot diseases caused by necrotrophs in wheat is controlled by three different systems including S genes, R genes and QTLs. The genes belonging to all these three categories are now known for at least three of the four diseases (except for STB, for which S genes are not known; instead SSP genes are known). Markers associated with all these three systems are also known now. The number of markers for desirable genes will certainly be large (at least >20), thus making simple backcross or forward breeding approaches not suitable. Therefore, we recommend the use of either marker assisted recurrent selection (MARS) or QTL-based genomic selection (GS). Other possible molecular approaches include the use of genome editing, base/prime editing, and gene drive. The utility of these molecular approaches has already been demonstrated; these strategies are briefly described in this section.

### Marker-assisted recurrent selection

MARS involving more than 20 markers and two or more than two cycles of recombination may be used as a suitable breeding strategy. Such an approach has already been successfully utilized by Rahman et al. (2020) for disease resistance involving crown rot disease in wheat. In this study, 22 markers could be recombined using two recombination cycles.

### GS using QTLs and MTAs

In two recent studies, one each in maize and wheat, it has been shown that the prediction accuracy of genomic selection can be improved by using only those markers, which are known to be associated with QTLs or MTAs (Liu et al., 2019; Zaim et al., 2020). This strategy may be used for improvement of resistance against the four pathogens under review. Since we already have a large number of disease-associated markers, a selected set of polymorphic markers may be used in training population for estimation of breeding values, which may then be used for selecting desirable plants in the segregating breeding population.

### Genome/base editing and synthetic gene drives

Genome editing, base editing and gene drives are three new approaches, which can also be used for resistance breeding. Several examples are available, where susceptibility genes in the host have been modified using CRISPR/Cas technology (for a review, see Borrelli et al., 2018; Tyagi et al., 2020; Paul et al., 2021; Negi et al., 2022). In wheat also, resistance against powdery mildew has been successfully achieved using this technology (Wang et al., 2014; Zhang et al., 2017). Base editing and prime editing are two other more efficient recent approaches, which have already been used for crop improvement (for a review, see Azameti and Dauda, 2021) and will certainly be used in future for disease resistance in wheat.

More recently, synthetic gene drives are being tried for creating bias in the inheritance of a particular DNA sequence, such that it can be used for increasing the frequency of genes/alleles that may spread and reduce the pathogen populations with virulence genes causing the diseases. The introduced gene/allele puts the pathogen at a disadvantage, and can be made to spread the altered desired trait throughout the population. Many such systems occur naturally, and will facilitate the development of new gene drives using synthetic biology techniques. For resistance breeding, synthetic gene drives may be used to modify either the susceptibility gene of the host or the virulence gene of the pathogen, so that either the host will lose susceptibility or the pathogen will lose virulence. If synthetically modified populations of the pathogen are released in wheat fields, this will soon spread in the pathogen population, and render the wheat cultivar resistant.

### Disease management

In integrated management, resistant cultivars may be used along with cultural practices and fungicide application. Since infected seed and straw serve as the primary source of inoculum, seed treatment, crop rotation, and residue management may also prove useful in avoiding an epidemic in disease-prone areas. Also, since SNB infection causes the greatest yield losses at the adult plant stage, resistance screening may also be useful (Francki, 2013).

## Conclusions and future perspectives

Disease resistance in plants, including wheat, can be race-specific or race-nonspecific, the latter sometimes also described as adult plant resistance. Both these types of disease resistance are generally controlled by R genes, which have been the subjects of detailed studies. The plant immunity involving these R genes has also been subjected to detailed studies at the molecular level, developing a zig-zag model involving PTI, effector triggered susceptibility (ETS) and effector triggered immunity (ETI) (Jones and Dangl, 2006). During the last >25 years, >300 R genes and several Avr genes have been cloned, thus providing an opportunity to study the interaction between the products of R genes of the host and the corresponding Avr genes in the pathogen at the molecular level (for a review, see Kourelis and van der Hoorn, 2018). However, studies on Avr genes have yet to be undertaken on a war scale to become comparable to those on R genes. In most examples of R genes, the GFG relationship proposed by Flor (1942), Flor (1956) holds good, although gene-for-gene models involving multiple genes have also been suggested (Sasaki, 2000; Fenton et al., 2009). However, disease resistance controlled by the absence (or presence of recessive alleles) of susceptibility/sensitivity genes like SWEET genes for bacterial blight (BB) in rice (Gupta, 2020) and S genes like *Tsn1* in wheat follow an inverse IGFG relationship with corresponding NE genes in the pathogen (Navathe et al., 2020). This is an area of research on disease resistance, which has witnessed immense activity in recent years. As a result, several S genes in wheat for three of the four important diseases covered in this review, namely SNB, TS and SB and the corresponding NE genes in the form of NE producing genes or *Tox* genes) in the pathogens have been identified. Some of these genes (both S genes in the host and NE genes in the pathogen) have also been cloned and characterized, generating information about the molecular mechanism involved in plant immunity involving these pathosystems. In summary, perhaps only about a dozen NE genes and an equal number of corresponding S genes are now known. In future, more S genes in wheat and other crops and the corresponding NE Tox genes in the pathogens exhibiting IGFG may be discovered; it will be interesting to find out if S genes occur for STB also, although there is little chance, because despite detailed studies already undertaken, no S genes for STB have been discovered so far. It will, therefore, be interesting to find out the reasons for the absence of S gene and the implications of the presence of as many as 22 R (Stb) genes and many SSP genes for STB, both in the host and the pathogen.

We also believe and hope that the subject dealing with S genes following the IGFG model will receive more attention in future. For instance, although much is known about the pathosystems dealing with SNB and TS, the information about IGFG dealing with SB has just started being generated and hardly any work is available on pathosystems involving NEs causing the following diseases: (i) FHB caused by *F. graminearum*; (ii) eyespot caused by *Tapesia yallundae* (syn *Pseudocercosporella herpotrichoides*, W-type anamorph); (iii) STB caused by *Z. tritici* and their corresponding S related gene in the host. We also believe that for diseases like SB caused by hemibiotrophs, GFG and IGFG may operate in parallel. Further studies involving the scoring of allelic states of genes involved in GFG and IGFG models need to be undertaken. In a recent study on spot blotch involving analysis of *Tsn1*-*ToxA* system following IGFG, we discovered that the wheat genotypes carrying recessive allele of S gene (*tsn1*) could also be susceptible and vice versa. Variation in the SB caused by *ToxA* positive isolates was also noticed (Navathe et al., 2020). This suggests that the relationship between a S gene in the host and the corresponding NE genes in the pathogen is not so simple, offering scope for further detailed investigations.

Another interesting area of future research is to examine interactions and cooperation between dominant R genes, recessive alleles of S genes and QTLs for providing disease resistance. One such study for SB has been planned by the authors of the present review, with the hope that useful information will be generated through such a study, which should prove useful in planning future strategies for breeding cultivars that would be resistant against the four-leaf spot diseases covered in the present review. Use of gene editing and base editing involving CRISPR/Cas for disease resistance will also certainly receive more attention in future.

## Author contributions

The subject of this review was conceived by PKG; All authors PKG, NKV, SS and AKJ contributed equally to this review.

## References

[B1] Abeysekara N. S. Faris J. D. Chao S. McClean P. E. Friesen T. L. (2012). Whole-genome QTL analysis of stagonospora nodorum blotch resistance and validation of the SnTox4*-Snn4* interaction in hexaploid wheat. Phytopathology 102, 94–104. doi: 10.1094/PHYTO-02-11-0040 21864084

[B2] Adhikari T. B. Jackson E. W. Gurung S. Hansen J. M. Bonman J. M. (2011). Association mapping of quantitative resistance to *Phaeosphaeria nodorum* in spring wheat landraces from the USDA national small grains collection. Phytopathology 101, 1301–1310. doi: 10.1094/PHYTO-03-11-0076 21692647

[B3] Adhikari T. B. Mamidi S. Gurung S. Bonman J. M. (2015). Mapping of new quantitative trait loci (QTL) for resistance to septoria tritici blotch in spring wheat (*Triticum aestivum* l.). Euphytica 205, 699–706. doi: 10.1007/s10681-015-1393-4

[B4] Aggarwal R. Agarwal S. Sharma S. Gurjar M. S. Bashyal B. M. Rao A. R.. (2022). Whole-genome sequence analysis of *Bipolaris sorokiniana* infecting wheat in India and characterization of *ToxA* gene in different isolates as pathogenicity determinants. 3 Biotech. 12, 151. doi: 10.1007/s13205-022-03213-3 PMC9209604 35747503

[B5] Aggarwal R. Sharma S. Singh K. Gurjar M. S. Saharan M. S. Gupta S.. (2019). First draft genome sequence of wheat spot blotch pathogen *Bipolaris sorokiniana* BS_112 from India, obtained using hybrid assembly. Microbiol. Res. Announc. 8, e00308–e00319. doi: 10.1128/MRA.00308-19 PMC6753261 31537657

[B6] Aguilar V. Stamp P. Winzeler M. Winzeler H. Schachermayr G. Keller B.. (2005). Inheritance of field resistance to stagonospora nodorum leaf and glume blotch and correlations with other morphological traits in hexaploid wheat (*Triticum aestivum* l.). Theor. Appl. Genet. 111, 325–336. doi: 10.1007/s00122-005-2025-5 15895203

[B7] Ali S. Francl L. J. (2002). Race structure of *Pyrenophora tritici*-repentis isolates obtained from wheat in south America. Plant Prot. Sci. 38, 302–304. doi: 10.17221/10473-PPS

[B8] Ali S. Francl L. J. (2003). Population race structure of *Pyrenophora tritici-repentis* prevalent on wheat and noncereal grasses in the great plains. Plant Dis. 87, 418–422. doi: 10.1094/PDIS.2003.87.4.418 30831839

[B9] Ali S. Singh P. K. McMullen M. P. Mergoum M. Adhikari T. B. (2008). Resistance to multiple leaf spot diseases in wheat. Euphytica 159, 167–179. doi: 10.1007/s10681-007-9469-4

[B10] Al-Sadi A. M. (2021). *Bipolaris sorokiniana*-induced black point, common root rot, and spot blotch diseases of wheat: a review. Front. Cell Infect. Microbiol. 11. doi: 10.3389/fcimb.2021.584899 PMC7991903 33777829

[B11] Arseniuk E. Czembor P. C. Czaplicki A. Song Q. Cregan P. B. Hoffman D. L.. (2004). QTL controlling partial resistance to stagonospora nodorum leaf blotch in winter wheat cultivar alba. Euphytica 137, 225–231. doi: 10.1023/B:EUPH.0000041589.47544.de

[B12] Azameti M. K. Dauda W. P. (2021). Base editing in plants: Applications, challenges, and future prospects. Front. Plant Sci. 12. doi: 10.3389/fpls.2021.664997 PMC8353127 34386023

[B13] Ballance G. M. Lamari L. Bernier C. C. (1989). Purification and characterization of a host-selective necrosis toxin from *Pyrenophora tritici*-*repentis* . Physiol. Mol. Plant Pathol. 35, 203–213. doi: 10.1016/0885-5765(89)90051-9

[B14] Ballance G. M. Lamari L. Kowatsch R. Bernier C. C. (1996). Cloning, expression, and occurrence of the gene encoding the ptr necrosis toxin from *Pyrenophora tritici*-*repentis* . Mol. Plant Pathol. doi: 10.1007/978-94-011-5218-1_21

[B15] Ballance G. M. Lamari L. Kowatsch R. Bernier C. C. (1998). “The ptr necrosis toxin and necrosis toxin gene from pyrenophora tritici-repentis,” in Molecular genetics of host-specific toxins in plant disease, vol. 13. Eds. Kohmoto K. Yoder. O. C. (Netherland: Springer), 177–185.

[B16] Ben M’Barek S. Cordewener J. H. Tabib Ghaffary S. M. van der Lee T. A. Liu Z. Gohari A. M.. (2015). FPLC and liquid-chromatography mass spectrometry identify candidate necrosis-inducing proteins from culture filtrates of the fungal wheat pathogen *Zymoseptoria tritici* . Fungal Genet. Biol. 79, 54–62. doi: 10.1016/j.fgb.2015.03.015 26092790

[B17] Bertucci M. Brown-Guedira G. Murphy J. P. Cowger C. (2014). Genes conferring sensitivity to *Stagonospora nodorum* necrotrophic effectors in stagonospora nodorum blotch-susceptible U.S. wheat cultivars. Plant Dis. 98, 746–753. doi: 10.1094/PDIS-08-13-0820-RE 30708627

[B18] Bhathal J. S. Loughman R. Speijers J. (2003). Yield reduction in wheat in relation to leaf disease from yellow (tan) spot and septoria nodorum blotch. Eur. J. Plant Pathol. 109, 435–443. doi: 10.1023/A:1024277420773

[B19] Blixt E. Olson Å. Högberg N. Djurle A. Yuen J. (2008). Mating type distribution and genetic structure are consistent with sexual recombination in the Swedish population of *Phaeosphaeria nodorum* . Plant Pathol. 57, 634–641. doi: 10.1111/j.1365-3059.2008.01826.x

[B20] Borrelli V. M. G. Brambilla V. Rogowsky P. Marocco A. Lanubile A. (2018). The enhancement of plant disease resistance using CRISPR/Cas9 technology. Front. Plant Sci. 9. doi: 10.3389/fpls.2018.01245 PMC6117396 30197654

[B21] Brading P. A. Verstappen E. C. P. Kema G. H. J. Brown J. K. M. (2002). A gene-for-gene relationship between wheat and *Mycosphaerella graminicola*, the *Septoria tritici blotch pathogen* . Phytopathology 92, 439–445. doi: 10.1094/PHYTO.2002.92.4.439 18942957

[B22] Brown J. K. Chartrain L. Lasserre-Zuber P. Saintenac C. (2015). Genetics of resistance to *Zymoseptoria tritici* and applications to wheat breeding. Fungal Genet. Biol. 79, 33–41. doi: 10.1016/j.fgb.2015.04.017 26092788 PMC4510316

[B23] Chand R. Navathe S. Sharma S. (2021). “Advances in breeding techniques for durable resistance to spot blotch in cereals,” in Achieving durable disease resistance in cereals (Burleigh Dodds Series in Agricultural Science (London: Burleigh Dodds Science Publishing), 435–474. doi: 10.19103/AS.2021.0092.18

[B24] Chartrain L. Brading P. A. Brown J. K. M. (2005c). Presence of the Stb6 gene for resistance to septoria tritici blotch (*Mycosphaerella graminicola*) in cultivars used in wheat-breeding programmes worldwide. Plant Pathol. 54, 134–143. doi: 10.1111/j.1365-3059.2005.01164.x

[B25] Chu C.-G. Chao S. Friesen T. L. Faris J. D. Zhong S. Xu S. S. (2010). Identification of novel tan spot resistance QTLs using an SSR-based linkage map of tetraploid wheat. Mol. Breed. 25, 327–338. doi: 10.1007/s11032-009-9335-2

[B26] Ciuffetti L. M. Manning V. A. Pandelova I. Betts M. F. Martinez J. P. (2010). Host-selective toxins. ptr ToxA and ptr ToxB, as necrotrophic effectors in the *Pyrenophora tritici*-*repentis*-wheat interaction. New Phytol. 187, 911–919. doi: 10.1111/j.1469-8137.2010.03362.x 20646221

[B27] Ciuffetti L. M. Tuori R. P. Gaventa J. M. (1997). A single gene encodes a selective toxin causal to the development of tan spot of wheat. Plant Cell 9, 135–144. doi: 10.1105/tpc.9.2.135 9061946 PMC156906

[B28] Cockram J. Scuderi A. Barber T. Furuki E. Gardner K. A. Gosman N.. (2015). Fine-mapping the wheat *Snn1* locus conferring sensitivity to the *Parastagonospora nodorum* necrotrophic effector SnTox1 using an eight founder multiparent advanced generation inter-cross population. G3 5, 2257–2266. doi: 10.1534/g3.115.021584 26416667 PMC4632045

[B29] Conner R. L. (1990). Interrelationship of cultivar reactions to common root rot, black point, and spot blotch in spring wheat. Plant Dis. 74, 224–227. doi: 10.1094/PD-74-0224

[B30] Corsi B. Percival-Alwyn L. Downie R. C. Venturini L. Iagallo E. M. Campos Mantello C. C.. (2020). Genetic analysis of wheat sensitivity to the ToxB fungal effector from *Pyrenophora tritici*-*repentis*, the causal agent of tan spot. Theor. Appl. Genet. 133, 935–950. doi: 10.1007/s00122-019-03517-8 31915874 PMC7021774

[B31] Rivera-Burgos L. A. Brown-Guedira G. Johnson J. Mergoum M. Cowger C. (2022). Accounting for heading date gene effects allows detection of small-effect QTL associated with resistance to septoria nodorum blotch in wheat. PloS One 17, e0268546. doi: 10.1371/journal.pone.0268546 35588401 PMC9119491

[B32] Cowger C. Ward B. Brown-Guedira G. Brown J. K. M. (2020). Role of effector-sensitivity gene interactions and durability of quantitative resistance to septoria nodorum blotch in eastern U.S. wheat. Front. Plant Sci. 11. doi: 10.3389/fpls.2020.00155 PMC7067980 32210986

[B33] Croll D. McDonald B. A. (2012). The accessory genome as a cradle for adaptive evolution in pathogens. PloS Pathog. 8, e1002608. doi: 10.1371/journal.ppat.1002608 22570606 PMC3343108

[B34] Crook A. D. Friesen T. L. Liu Z. H. Ojiambo P. S. Cowger C. (2012). Novel necrotrophic effectors from *Stagonospora nodorum* and corresponding host sensitivities in winter wheat germplasm in the southeastern united states. Phytopathology 102, 498–505. doi: 10.1094/PHYTO-08-11-0238 22494247

[B35] Czembor P. C. Arseniuk E. Czaplicki A. Song Q. J. Cregan P. B. Ueng P. P. (2003). QTL mapping of partial resistance in winter wheat to stagonospora nodorum blotch. Genome 46, 546–554. doi: 10.1139/g03-036 12897862

[B36] Czembor P. C. Arseniuk E. Radecka-Janusik M. Piechota U. Słowacki P. (2019). Quantitative trait loci analysis of adult plant resistance to parastagonospora nodorum blotch in winter wheat cv. liwilla (*Triticum aestivum* l.). Eur. J. Plant Pathol. 155, 1001–1016. doi: 10.1007/s10658-019-01829-5

[B37] Dastur J. F. (1942). Notes on some fungi isolated from “black point” affected kernels in the central provinces. Ind. J. Agric. Sci. 12, 731–742.

[B38] Desmazieres J. B. H. J. (1842). Neuvieme notice sur quelques plantes cryptogames, la plupart inedites, recemment decouvertes en France, et que vont paraitre en nature dans la collection publiee par l’auteur. Ann. Des. Sci. Nat. Bot. Ser. 2, 91–107.

[B39] Dinglasan E. G. Godwin I. D. Phan H. T. T. Tan K.-C. Platz G. J. Hickey L. T. (2017). Vavilov wheat accessions provide useful sources of resistance to tan spot (syn. yellow spot) of wheat. Plant Pathol. 67, 1076–1087. doi: 10.1111/ppa.12822

[B40] Dinglasan E. G. Singh D. Shankar M. Afanasenko O. Platz G. Godwin I. D.. (2019). Discovering new alleles for yellow spot resistance in the vavilov wheat collection. Theor. Appl. Genet. 132, 149–162. doi: 10.1007/s00122-018-3204-5 30327845

[B41] Downie R. C. Bouvet L. Furuki E. Gosman N. Gardner K. A. Mackay I. J.. (2018). Assessing European wheat sensitivities to *Parastagonospora nodorum* necrotrophic effectors and fine-mapping the *Snn3-B1* locus conferring sensitivity to the effector SnTox3. Front. Plant Sci. 9. doi: 10.3389/fpls.2018.00881 PMC6039772 30022985

[B42] Downie R. C. Lin M. Corsi B. Ficke A. Lillemo M. Oliver R. P.. (2021). Septoria nodorum blotch of wheat: disease management and resistance breeding in the face of shifting disease dynamics and a changing environment. Phytopathology 11, 906–920. doi: 10.1094/PHYTO-07-20-0280-RVW 33245254

[B43] Drechsler C. (1923). Some graminicolons species of *Helminthosporium* . Int. J. Agric. Res. 24, 614–670.

[B44] Effertz R. J. Meinhardt S. W. Anderson J. A. Jordahl J. G. Francl L. J. (2002). Identification of a chlorosis-inducing toxin from *Pyrenophora tritici-*repentis and the chromosomal location of an insensitivity locus in wheat. Phytopathology 92, 527–533. doi: 10.1094/PHYTO.2002.92.5.527 18943027

[B45] Eyal Z. (1973). Physiologic specialization of *Septoria tritici* . Phytopathology 63, 1087–1091. doi: 10.1094/Phyto-63-1087

[B46] Eyal Z. Scharen A. L. Prescott J. M. van Ginkel M. (1987). The septoria diseases of wheat: Concepts and methods of disease management (Mexico: CIMMYT).

[B47] Faris J. D. Friesen T. L. (2020). Plant genes hijacked by necrotrophic fungal pathogens. Curr. Opin. Plant Biol. 56, 74–80. doi: 10.1016/j.pbi.2020.04.003 32492572

[B48] Faris J. D. Liu Z. Xu S. S. (2013). Genetics of tan spot resistance in wheat. Theor. Appl. Genet. 126, 2197–2217. doi: 10.1007/s00122-013-2157-y 23884599

[B49] Faris J. D. Overlander M. E. Kariyawasam G. K. Carter A. Xu S. S. Liu Z. (2020). Identification of a major dominant gene for race-nonspecific tan spot resistance in wild emmer wheat. Theor. Appl. Genet. 133, 829–841. doi: 10.1007/s00122-019-03509-8 31863156

[B50] Faris J. D. Zhang Z. Lu H. J. Lu S. W. Reddy L. Cloutier S.. (2010). A unique wheat disease resistance-like gene governs effector-triggered susceptibility to necrotrophic pathogens. Proc. Nat’l. Acad. Sci. U.S.A. 107, 13544–13549. doi: 10.1073/pnas.1004090107 20624958 PMC2922177

[B51] Fenton A. Antonovics J. Brockhurst M. A. (2009). Inverse-gene-for-gene infection genetics and coevolutionary dynamics. Am. Nat. 174, E230–E242. doi: 10.1086/645087 19852618

[B52] Ficke A. Cowger C. Bergstrom G. Brodal G. (2018). Understanding yield loss and pathogen biology to improve disease management: Septoria nodorum blotch -a case study in wheat. Plant Dis. 102, 696–707. doi: 10.1094/PDIS-09-17-1375-FE 30673402

[B53] Figueroa M. Hammond-Kosack K. E. Solomon P. S. (2018). A review of wheat diseases: a field perspective. Mol. Plant Pathol. 19, 1523–1536. doi: 10.1111/mpp.12618 29045052 PMC6638159

[B54] Flor H. H. (1942). Inheritance of pathogenicity in melampsora lini. Phytopathology 32, 653–669. 10.1094/Phyto-32-653 40934406

[B55] Flor H. H. (1956). The complementary genic systems in flax and flax rust. Adv. Genet. 8, 29–54. doi: 10.1016/S0065-2660(08)60498-8

[B56] Fones H. Gurr S. (2015). The impact of septoria tritici blotch disease on wheat: an EU perspective. Fungal Genet. Biol. 79, 3–7. doi: 10.1016/j.fgb.2015.04.004 26092782 PMC4502551

[B57] Francki M. G. (2013). Improving *Stagonospora nodorum* resistance in wheat: a review. Crop Sci. 53, 355–365. doi: 10.2135/cropsci2012.06.0347

[B58] Francki M. G. Shankar M. Walker E. Loughman R. Golzar H. Ohm H. (2011). New quantitative trait loci in wheat for flag leaf resistance to stagonospora nodorum blotch. Phytopathology 101, 1278–1284. doi: 10.1094/PHYTO-02-11-0054 21770777

[B59] Francki M. G. Walker E. Li D. A. Forrest K. (2018). High-density SNP mapping reveals closely linked QTL for resistance to stagonospora nodorum blotch (SNB) in flag leaf and glume of hexaploid wheat. Genome 61, 145–149. doi: 10.1139/gen-2017-0203 29237140

[B60] Francki M. G. Walker E. McMullan C. J. Morris W. G. (2020). Multi-location evaluation of global wheat lines reveal multiple QTL for adult plant resistance to septoria nodorum blotch (SNB) detected in specific environments and in response to different isolates. Front. Plant Sci. 11. doi: 10.3389/fpls.2020.00771 PMC7325896 32655592

[B61] Friesen T. L. Ali S. Klein K. K. Rasmussen J. (2005). Population genetic analysis of a global collection of *Pyrenophora tritici*-*repentis*, causal agent of tan spot of wheat. Phytopathology 95, 1144–1150. doi: 10.1094/PHYTO-95-1144 18943466

[B62] Friesen T. L. Chu C. G. Liu Z. H. Xu S. S. Halley S. Faris J. D. (2009). Host-selective toxins produced by *Stagonospora nodorum* confer disease susceptibility in adult wheat plants under field conditions. Theor. Appl. Genet. 118, 1489–1497. doi: 10.1007/s00122-009-0997-2 19266177

[B63] Friesen T. L. Faris J. D. (2021). Characterization of effector-target interactions in necrotrophic pathosystems reveals trends and variation in host manipulation. Ann. Rev. Phytopathol. 59, 77–98. doi: 10.1146/annurev-phyto-120320-012807 33909478

[B64] Friesen T. L. Holmes D. J. Bowden R. L. Faris J. D. (2018). *ToxA* is present in the U.S. *Bipolaris sorokiniana* population and is a significant virulence factor on wheat harboring *Tsn1* . Plant Dis. 102, 2446–2452. doi: 10.1094/PDIS-03-18-0521-RE 30252627

[B65] Friesen T. L. Meinhardt S. W. Faris J. D. (2007). The *Stagonospora nodorum*-wheat pathosystem involves multiple proteinaceous host-selective toxins and corresponding host sensitivity genes that interact in an inverse gene-for-gene manner. Plant J. 51, 681–692. doi: 10.1111/j.1365-313X.2007.03166.x 17573802

[B66] Friesen T. L. Stukenbrock E. H. Liu Z. Meinhardt S. Ling H. Faris J. D.. (2006). Emergence of a new disease as a result of interspecific virulence gene transfer. Nat. Genet. 38, 953–956. doi: 10.1038/ng1839 16832356

[B67] Friesen T. L. Zhang Z. Solomon P. S. Oliver R. P. Faris J. D. (2008). Characterization of the interaction of a novel *Stagonospora nodorum* host-selective toxin with a wheat susceptibility gene. Plant Physiol. 146, 682–693. doi: 10.1104/pp.107.108761 18065563 PMC2245837

[B68] Galagedara N. Liu Y. Fiedler J. Shi G. Chiao S. Xu S. S.. (2020). Genome-wide association mapping of tan spot resistance in a worldwide collection of durum wheat. Theor. Appl. Genet. 133, 2227–2237. doi: 10.1007/s00122-020-03593-1 32300825

[B69] Gao Y. Liu Z. Faris J. D. Richards J. Brueggeman R. S. Li X.. (2016). Validation of genome-wide association studies as a tool to identify virulence factors in *Parastagonospora nodorum* . Phytopathology 106, 1177–1185. doi: 10.1094/PHYTO-02-16-0113-FI 27442533

[B70] Ghaderi F. Sharifnabi B. Javan-Nikkhah M. Brunner P. C. McDonald B. A. (2020). SnToxA, SnTox1, and SnTox3 originated in *Parastagonospora nodorum* in the fertile crescent. Plant Pathol. 69, 1482–1491. doi: 10.1111/ppa.13233

[B71] Gilbert B. M. Wolpert T. J. (2013). Characterization of the LOV1-mediated, victorin-induced, cell-death response with virus-induced gene silencing. Mol. Plant Microbe Interact. 26, 903–917. doi: 10.1094/MPMI-01-13-0014-R 23634836

[B72] Gohari A. M. (2015). Identification and functional characterization of putative virulence factors in the fungal wheat pathogen zymoseptoria tritici. [PhD thesis] (Wageningen: Wageningen University).

[B73] Gonzalez-Hernandez J. L. Singh P. K. Mergoum M. Adhikari T. B. Kianian S. F. Simsek S.. (2009). A quantitative trait locus on chromosome 5B controls resistance of *Triticum turgidum* (L.) var. diccocoides to stagonospora nodorum blotch. Euphytica 166, 199. doi: 10.1007/s10681-008-9825-z

[B74] Guo J. Shi G. Liu Z. (2018). Characterizing virulence of the *Pyrenophora tritici*-*repentis* isolates lacking both *ToxA* and *ToxB* genes. Pathogens 7, 74. doi: 10.3390/pathogens7030074 30213041 PMC6161158

[B75] Gupta P. K. (2020). *SWEET* genes for disease resistance in plants. Trends Genet. 36, 901–904. doi: 10.1016/j.tig.2020.08.007 32896434

[B76] Gupta P. K. Chand R. Vasistha N. K. Pandey S. P. Kumar U. Mishra V. K.. (2018a). Spot blotch disease of wheat: the current status of research on genetics and breeding. Plant Pathol. 67, 508–531. doi: 10.1111/ppa.12781

[B77] Gupta P. K. Vasistha N. K. Aggarwal R. Joshi A. K. (2018b). Biology of *B. sorokiniana* (syn. *Cochliobolus sativus*) in genomics era. J. Plant Biochem. Biotechnol. 27, 123–138. doi: 10.1007/s13562-017-0426-6

[B78] Gurung S. Bonman J. M. Ali S. Patel J. Myrfield M. Mergoum M.. (2009). New and diverse sources of multiple disease resistance in wheat. Crop Sci. 49, 1655–1666. doi: 10.2135/cropsci2008.10.0633

[B79] Gurung S. Mamidi S. Bonman J. M. Xiong M. Brown-Guedira G. Adhikari T. B. (2014). Genome-wide association study reveals novel quantitative trait loci associated with resistance to multiple leaf spot diseases of spring wheat. PloS One 9, e108179. doi: 10.1371/journal.pone.0108179 25268502 PMC4182470

[B80] Hafez M. Gourlie R. Despins T. Turkington T. K. Friesen T. L. Aboukhaddour R. (2020). *Parastagonospora nodorum* and related species in Western Canada: genetic variability and effector genes. Phytopathology 110, 1946–1958. doi: 10.1094/PHYTO-05-20-0207-R 32689900

[B81] Halder J. Zhang J. Ali S. Sidhu J. S. Gill H. S. Talukder S. K.. (2019). Mining and genomic characterization of resistance to tan spot, stagonospora nodorum blotch (SNB), and fusarium head blight in Watkins core collection of wheat landraces. BMC Plant Biol. 19, 480. doi: 10.1186/s12870-019-2093-3 31703626 PMC6839225

[B82] Hane J. K. Lowe R. G. Solomon P. S. Tan K. C. Schoch C. L. Spatafora J. W.. (2007). *Dothideomycete* plant interactions illuminated by genome sequencing and EST analysis of the wheat pathogen *Stagonospora nodorum* . Plant Cell 19, 3347–3368. doi: 10.1105/tpc.107.052829 18024570 PMC2174895

[B83] Hehir J. G. Connolly C. O’Driscoll A. Lynch J. P. Spink J. Brown J. K. M.. (2018). Temporal and spatial field evaluations highlight the importance of the presymptomatic phase in supporting strong partial resistance in *Triticum aestivum* against *Zymoseptoria tritici* . Plant Pathol. 67, 573–583. doi: 10.1111/ppa.12780

[B84] Jighly A. Alagu M. Makdis F. Singh M. Singh S. Emebiri L. C.. (2016). Genomic regions conferring resistance to multiple fungal pathogens in synthetic hexaploid wheat. Mol. Breed. 36, 127. doi: 10.1007/s11032-016-0541-4

[B85] Jones J. D. Dangl J. L. (2006). The plant immune system. Nature 444, 323–329. doi: 10.1038/nature05286 17108957

[B86] Juliana P. Singh R. P. Singh P. K. Poland J. A. Bergstrom G. C. Huerta-Espino J.. (2018). Genome-wide association mapping for resistance to leaf rust, stripe rust and tan spot in wheat reveals potential candidate genes. Theor. Appl. Genet. 131, 1405–1422. doi: 10.1007/s00122-018-3086-6 29589041 PMC6004277

[B87] Kader K. A. Hunger R. M. Sreedharan A. Marek S. M. (2022). Races, disease symptoms and genetic variability in *Pyrenophora tritici-repentis* isolates from Oklahoma that cause tan spot of winter wheat. Cereal Res. Commun. 50, 273–280. doi: 10.1007/s42976-021-00175-9

[B88] Kariyawasam G. K. Richards J. K. Wyatt N. A. Running K. Xu S. S. Liu Z.. (2021). The *Parastagonospora nodorum* necrotrophic effector SnTox5 targets the wheat gene *Snn5* and facilitates entry into the leaf mesophyll. New Phytol. 233, 409–426. doi: 10.1111/nph.17602 34231227 PMC9291777

[B89] Karki S. J. Reilly A. Zhou B. Mascarello M. Burke J. Doohan F.. (2021). A small secreted protein from *Zymoseptoria tritici* interacts with a wheat E3 ubiquitin ligase to promote disease. J. Exp. Bot. 72, 733–746. doi: 10.1093/jxb/eraa489 33095257 PMC7853600

[B90] Kaur B. Bhatia D. Mavi G. S. (2021). Eighty years of gene-for-gene relationship and its applications in identification and utilization of r genes. J. Genet. 100, 1–17. doi: 10.1007/s12041-021-01300-7 34282731

[B91] Kema G. H. J. Gohari A. M. Aouini L. Gibriel H. A. Y. Ware S. B. van den Bosch F.. (2018). Stress and sexual reproduction affect the dynamics of the wheat pathogen effector *AvrStb6* and *strobilurin* resistance. Nat. Genet. 50, 375–380. doi: 10.1038/s41588-018-0052-9 29434356

[B92] Kema G. H. J. Verstappen E. C. P. Waalwijk C. (2000). Avirulence in the wheat septoria tritici leaf blotch fungus *Mycosphaerella graminicola* is controlled by a single locus. Mol. Plant Microbe Interact. 13, 1375–1379. doi: 10.1094/MPMI.2000.13.12 11106030

[B93] Kema G. H. J. Yu D. Rijkenberg F. H. J. Shaw M. W. Baayen R. P. (1996). Histology of the pathogenesis of *Mycosphaerella graminicola* in wheat. Phytopathology 86, 777–786. doi: 10.1094/Phyto-86-777

[B94] Keon J. Antoniw J. Carzaniga R. Deller S. Ward J. L. Baker J. M.. (2007). Transcriptional adaptation of *Mycosphaerella graminicola* to programmed cell death (PCD) of its susceptible wheat host. Mol. Plant Microbe Interact. 20, 178–193. doi: 10.1094/MPMI-20-2-0178 17313169

[B95] Kettles G. J. Bayon C. Canning G. Rudd J. J. Kanyuka K. (2017). Apoplastic recognition of multiple candidate effectors from the wheat pathogen *Zymoseptoria tritici* in the nonhost plant *Nicotiana benthamiana* . New Phytol. 213, 338–350. doi: 10.1111/nph.14215 27696417 PMC5132004

[B96] Kettles G. J. Kanyuka K. (2016). Dissecting the molecular interactions between wheat and the fungal pathogen *Zymoseptoria tritici* . Front. Plant Sci. 7. doi: 10.3389/fpls.2016.00508 PMC4832604 27148331

[B97] King J. E. Cook R. J. Melville S. C. (1983). A review of septoria diseases of wheat and barley. Ann. Appl. Biol. 103, 345–373. doi: 10.1111/j.1744-7348.1983.tb02773.x

[B98] Kokhmetova A. Sehgal D. Ali S. Atishova M. Kumarbayeva M. Leonova I.. (2020). Genome-wide association study of tan spot resistance in a hexaploid wheat collection from Kazakhstan. Front. Genet. 11. doi: 10.3389/fgene.2020.581214 PMC7831376 33505423

[B99] Kollers S. Rodemann B. Ling J. Korzun V. Ebmeyer E. Argillier O.. (2014). Genome-wide association mapping of tan spot resistance (*Pyrenophora tritici*-*repentis*) in European winter wheat. Mol. Breed. 34, 363–371. doi: 10.1007/s11032-014-0039-x

[B100] Korte A. Farlow A. (2013). The advantages and limitations of trait analysis with GWAS: a review. Plant Methods 9, 29. doi: 10.1186/1746-4811-9-29 23876160 PMC3750305

[B101] Kourelis J. van der Hoorn R. A. L. (2018). Defended to the nines: 25 years of resistance gene cloning identifies nine mechanisms for r protein function. Plant Cell 30, 285–299. doi: 10.1105/tpc.17.00579 29382771 PMC5868693

[B102] Kumar S. Roder M. S. Tripathi S. B. Kumar S. Chand R. Joshi A. K.. (2015). Mendelization and fine mapping of a bread wheat spot blotch disease resistance QTL. Mol. Breed. 35, 218. doi: 10.1007/s11032-015-0411-5

[B103] Lamari L. Gilbert J. Tekauz A. (1998). Race differentiation in *Pyrenophora tritici*-*repentis* and survey of physiologic variation in western Canada. Can. J. Plant Pathol. 20, 396–400. doi: 10.1080/07060669809500410

[B104] Lamari L. Strelkov S. E. Yahyaoui A. Orabi J. Smith R. B. (2003). The identification of two new races of *Pyrenophora tritici*-*repentis* from the host center of diversity confirms a one-to-one relationship in tan spot of wheat. Phytopathology 93, 391–396. doi: 10.1094/PHYTO.2003.93.4.391 18944352

[B105] Lebrun M. H. Langin T. Kroj T. Cockram J. Oliver R. Kema G. (2016). “Wheat effector assisted breeding for resistance to fungal pathogens (WEAB),” in (JJC)-11emes rencontres de phytopathologie-mycologie, societe française de phytopathologie (SFP), vol. 49Ed. Chevaugeon J.J.

[B106] Leng Y. Zhao M. Fiedler J. Dreiseitl A. Chao S. Li X.. (2020). Molecular mapping of loci conferring susceptibility to spot blotch and resistance to powdery mildew in barley using the sequencing-based genotyping approach. Phytopathology 110, 440–446. doi: 10.1094/PHYTO-08-19-0292-R 31609681

[B107] Leng Y. Zhao M. Wang R. Steffenson B. J. Brueggeman R. S. Zhong S. (2018). The gene conferring susceptibility to spot blotch caused by *Cochliobolus sativus* is located at the mla locus in barley cultivar bowman. Theor. Appl. Genet. 131, 1531–1539. doi: 10.1007/s00122-018-3095-5 29663053

[B108] Li D. Walker E. Francki M. (2021). Genes associated with foliar resistance to septoria nodorum blotch of hexaploid wheat (*Triticum aestivum l*.). In. J. Mol. Sci. 22, 5580. doi: 10.3390/ijms22115580 PMC8197541 34070394

[B109] Li Q. Wang B. Yu J. Dou D. (2021). Pathogen-informed breeding for crop disease resistance. J. Integr. Plant Biol. 63, 305–311. doi: 10.1111/jipb.13029 33095498

[B110] Lillemo M. Joshi A. K. Prasad R. Chand R. Singh R. P. (2013). QTL for spot blotch resistance in bread wheat line Saar co-locate to the biotrophic disease resistance loci *Lr34* and *Lr46* . Theor. Appl. Genet. 126, 711–719. doi: 10.1007/s00122-012-2012-6 23139144

[B111] Lin M. Corsi B. Ficke A. Tan K. C. Cockram J. Lillemo M. (2020a). Genetic mapping using a wheat multi-founder population reveals a locus on chromosome 2A controlling resistance to both leaf and glume blotch caused by the necrotrophic fungal pathogen *Parastagonospora nodorum* . Theor. Appl. Genet. 133, 785–808. doi: 10.1007/s00122-019-03507-w 31996971 PMC7021668

[B112] Lin M. Ficke A. Cockram J. Lillemo M. (2020b). Genetic structure of the Norwegian *Parastagonospora nodorum* population. Front. Microbiol. 11. doi: 10.3389/fmicb.2020.01280 PMC7309014 32612592

[B113] Lin M. Ficke A. Dieseth J. A. Lillemo M. (2022). Genome-wide association mapping of septoria nodorum blotch resistance in Nordic winter and spring wheat collections. Theor. Appl. Genet. 135, 4169–4182. doi: 10.1007/s00122-022-04210-z 36151405 PMC9734210

[B114] Lin M. Lillemo M. (2021). “Advances in genetic mapping of septoria nodorum blotch resistance in wheat and applications in resistance breeding” Achieving durable disease resistance in cereals”, in Burleigh Dodds Series in Agricultural Science, (Cambridge: Burleigh Dodds Science Publishing)

[B115] Lin M. Stadlmeier M. Mohler V. Tan K. C. Ficke A. Cockram J.. (2021). Identification and cross-validation of genetic loci conferring resistance to septoria nodorum blotch using a German multi-founder winter wheat population. Theor. Appl. Genet. 134, 125–142. doi: 10.1007/s00122-020-03686-x 33047219 PMC7813717

[B116] Liu Z. H. El-Basyoni I. Kariyawasam G. Zhang G. Fritz A. Hansen J.. (2015). Evaluation and association mapping of resistance to tan spot and stagonospora nodorum blotch in adapted winter wheat germplasm. Plant Dis. 99, 1333–1341. doi: 10.1094/PDIS-11-14-1131-RE 30690997

[B117] Liu Z. H. Faris J. D. Oliver R. P. Tan K. C. Solomon P. S. McDonald M. C.. (2009). SnTox3 acts in effector triggered susceptibility to induce disease on wheat carrying the *Snn3* gene. PloS Pathog. 5, e1000581. doi: 10.1371/journal.ppat.1000581 19806176 PMC2736379

[B118] Liu Z. H. Friesen T. L. Rasmussen J. B. Ali S. Meinhardt S. W. Faris J. D. (2004a). Quantitative trait loci analysis and mapping of seedling resistance to stagonospora nodorum leaf blotch in wheat. Phytopathology 94, 1061–1067. doi: 10.1094/PHYTO.2004.94.10.1061 18943794

[B119] Liu Z. H. Gao Y. Kim Y. M. Faris J. D. Shelver W. L. de Wit P.J.G.M. (2016). SnTox1, a *Parastagonospora nodorum* necrotrophic effector, is a dual-function protein that facilitates infection while protecting from wheat-produced chitinases. New Phytol. 211, 1052–1064. doi: 10.1111/nph.13959 27041151

[B120] Liu Y. Salsman E. Wang R. Galagedara N. Zhang Q. Fiedler J. D.. (2020b). Meta-QTL analysis of tan spot resistance in wheat. Theor. Appl. Genet. 133, 2363–2375. doi: 10.1007/s00122-020-03604-1 32436020

[B121] Liu X. Wang H. Hu X. Li K. Liu Z. Wu Y.. (2019). Improving genomic selection with quantitative trait loci and nonadditive effects revealed by empirical evidence in maize. Front. Plant Sci. 10. doi: 10.3389/fpls.2019.01129 PMC6759780 31620155

[B122] Liu Z. H. Zhang Z. Faris J. D. Oliver R. P. Syme R. McDonald M. C.. (2012). The cysteine rich necrotrophic effector SnTox1 produced by *Stagonospora nodorum* triggers susceptibility of wheat lines harboring *Snn1* . PloS Pathog. 8, e1002467. doi: 10.1371/journal.ppat.1002467 22241993 PMC3252377

[B123] Liu Y. Zhang Q. Salsman E. Fiedler J. D. Hegstad J. B. Liu Z.. (2020a). QTL mapping of resistance to tan spot induced by race 2 of *Pyrenophora tritici*-*repentis* in tetraploid wheat. Theor. Appl. Genet. 133, 433–442. doi: 10.1007/s00122-019-03474-2 31720702

[B124] Lu P. Liang Y. Li D. Wang Z. Li W. Wang G.. (2016). Fine genetic mapping of spot blotch resistance gene *Sb3* in wheat (*Triticum aestivum*). Theor. Appl. Genet. 129, 577–589. doi: 10.1007/s00122-015-2649-z 26747045

[B125] Mago R. Tabe L. McIntosh R. A. Pretorius Z. Kota R. Paux E.. (2011). A multiple resistance locus on chromosome arm 3BS in wheat confers resistance to stem rust (*Sr2*), leaf rust (*Lr27*) and powdery mildew. Theor. Appl. Genet. 123, 615–623. doi: 10.1007/s00122-011-1611-y 21573954

[B126] Mahapatra S. S. Navathe S. Mishra V. K. Chand R. (2021). Biochemical profiling of seedling and adult plant and its association with spot blotch resistance in bread wheat. Russ. J. Plant Physiol. 68, 1265–1275. doi: 10.1134/S1021443721060133

[B127] Marshall R. Kombrink A. Motteram J. Loza-Reyes E. Lucas J. Hammond-Kosack K. E.. (2011). Analysis of two in planta expressed LysM effector homologs from the fungus *Mycosphaerella graminicola* reveals novel functional properties and varying contributions to virulence on wheat. Plant Physiol. 156, 756–769. doi: 10.1104/pp.111.176347 21467214 PMC3177273

[B128] Martinez J. P. Oesch N. W. Ciuffetti L. M. (2004). Characterization of the multiple-copy host-selective toxin gene, *ToxB*, in pathogenic and nonpathogenic isolates of *Pyrenophora tritici*-*repentis. Mol* . Plant Microbe Interact. 17, 467–474. doi: 10.1094/MPMI.2004.17.5.467 15141950

[B129] Martinez J. P. Ottum S. A. Ali S. Franci L. J. Ciuffetti L.M (2001)Characterization of the *ToxB* gene from *Pyrenophora tritici*-*repentis* . Mol. Plant Microbe Interact. 14, 675–677. doi: 10.1094/MPMI.2001.14.5.675 11332732

[B130] McDonald M. C. Ahren D. Simpfendorfer S. Milgate A. Solomon P. S. (2018). The discovery of the virulence gene *ToxA* in the wheat and barley pathogen *Bipolaris sorokiniana* . Mol. Plant Pathol. 19, 432–439. doi: 10.1111/mpp.12535 28093843 PMC6638140

[B131] McDonald B. A. Martinez J. P. (1991). Chromosome length polymorphisms in a *Septoria tritici* population. Curr. Genet. 19, 265–271. doi: 10.1007/BF00355053

[B132] McDonald M. C. Oliver R. P. Friesen T. L. Brunner P. C. McDonald B. A. (2013). Global diversity and distribution of three necrotrophic effectors in *Phaeosphaeria nodorum* and related species. New Phytol. 199, 241–251. doi: 10.1111/nph.12257 23550706

[B133] McDonald M. C. Razavi M. Friesen T. L. Brunner P. C. McDonald B. A. (2012). Phylogenetic and population genetic analyses of *Phaeosphaeria nodorum* and its close relatives indicate cryptic species and an origin in the fertile crescent. Fungal Genet. Biol. 49, 882–895. doi: 10.1016/j.fgb.2012.08.001 22922546

[B134] McDonald M. C. Solomon P. S. (2018). Just the surface: advances in the discovery and characterization of necrotrophic wheat effectors. Curr. Opin. Microbiol. 46, 14–18. doi: 10.1016/j.mib.2018.01.019 29452845

[B135] McIntosh R. A. Yamazaki Y. Devos K. M. Dubcovsky J. Rogers J. Appels R. (2007) Catalogue of gene symbols for wheat: 2007 supplement. KOMUGI integrated wheat science database. Available at: https://shigen.nig.ac.jp/wheat/komugi/genes/macgene/supplement2007.pdf.

[B136] McIntosh R. A. Yamazaki Y. Devos K. M. Dubcovsky J. Rogers J. Appels R. (2008) Catalogue of gene symbols for wheat:2008 supplement. KOMUGI integrated wheat science database. Available at: https://shigen.nig.ac.jp/wheat/komugi/genes/macgene/supplement2008.pdf.

[B137] Mehrabi R. Taga M. Kema G. H. J. (2007). Electrophoretic and cytological karyotyping of the foliar wheat pathogen *Mycosphaerella graminicola* reveals many chromosomes with a large size range. Mycologia 99, 804–812. doi: 10.1080/15572536.2007.11832518 18333510

[B138] Meile L. Croll D. Brunner P. C. Plissonneau C. Hartmann F. E. McDonald B. A.. (2018). A fungal avirulence factor encoded in a highly plastic genomic region triggers partial resistance to septoria tritici blotch. New Phytol. 219, 1048–1061. doi: 10.1111/nph.15180 29693722 PMC6055703

[B139] Mekonnen T. Haileselassie T. Goodwin S. B. Tesfayea K. (2020). Genetic diversity and population structure of *Zymoseptoria tritici* in Ethiopia as revealed by microsatellite markers. Fungal Genet. Biol. 141, 103413. doi: 10.1016/j.fgb.2020.103413 32442667

[B140] Moolhuijzen P. See P. T. Hane J. K. Shi G. Liu Z. Oliver R. P.. (2018). Comparative genomics of the wheat fungal pathogen *Pyrenophora tritici*-*repentis* reveals chromosomal variations and genome plasticity. BMC Genomics 19, 279. doi: 10.1186/s12864-018-4680-3 29685100 PMC5913888

[B141] Moolhuijzen P. See P. T. Moffat C. S. (2020). PacBio genome sequencing reveals new insights into the genomic organisation of the multi-copy *ToxB* gene of the wheat fungal pathogen *Pyrenophora tritici*-*repentis* . BMC Genomics 21, 645. doi: 10.1186/s12864-020-07029-4 32957933 PMC7507622

[B142] Muqaddasi Q. H. Kamal R. Mirdita V. Rodemann B. Ganal M. W. Reif J. C.. (2021). Genome-wide association studies and prediction of tan spot (*Pyrenophora tritici*-*repentis*) infection in European winter wheat *via* different marker platforms. Genes 12, 490. doi: 10.3390/genes12040490 33801723 PMC8103242

[B143] Murphy N. E. Loughman R. Appels R. Lagudah E. S. Jones M. G. K. (2000). Genetic variability in a collection of *Stagonospora nodorum isolates* from Western Australia. Aus. J. Agric. Res. 51, 679–684. doi: 10.1071/AR99107

[B144] Nagy E. D. Bennetzen J. L. (2008). Pathogen corruption and site-directed recombination at a plant disease resistance gene cluster. Genome Res. 18, 1918–1923. doi: 10.1101/gr.078766.108 18719093 PMC2593579

[B145] Navathe S. Yadav P. S. Chand R. Mishra V. K. Vasistha N. K. Meher P. K.. (2020). ToxA*–Tsn1* interaction for spot blotch susceptibility in Indian wheat: an example of inverse gene-for-gene relationship. Plant Dis. 104, 71–81. doi: 10.1094/PDIS-05-19-1066-RE 31697221

[B146] Negi C. Vasistha N. K. Singh D. Vyas P. Dhaliwal H. S. (2022). Application of CRISPR-mediated gene editing for crop improvement. Mol. Biotechnol. 64 (11), 1198–1217. doi: 10.1007/s12033-022-00507-y 35672603

[B147] Pal N. Jan I. Saini D. K. Kumar K. Kumar A. Sharma P. K.. (2022). Meta-QTLs for multiple disease resistance involving three rusts in common wheat (*Triticum aestivum* l.). Theor. Appl. Genet. 135, 2385–2405. doi: 10.1007/s00122-022-04119-7 35699741

[B148] Palma-Guerrero J. Ma X. Torriani S. F. F. Zala M. Francisco C. S. Hartmann F. E.. (2017). Comparative transcriptome analyses in *Zymoseptoria tritici* reveal significant differences in gene expression among strains during plant infection. Mol. Plant Microbe Interact. 30, 231–244. doi: 10.1094/MPMI-07-16-0146-R 28121239

[B149] Pascual L. Albert E. Sauvage C. Duangjit J. Bouchet J. P. Bitton F.. (2016). Dissecting quantitative trait variation in the resequencing era: complementarity of bi-parental, multi-parental, and association panels. Plant Sci. 242, 120–130. doi: 10.1016/j.plantsci.2015.06.017 26566830

[B150] Patel J. S. Mamidi S. Bonman J. M. Adhikari T. B. (2013). Identification of QTL in spring wheat associated with resistance to a novel isolate of *Pyrenophora tritici*-*repentis* . Crop Sci. 53, 842–852. doi: 10.2135/cropsci2012.01.0036

[B151] Paul N. C. Park S. W. Liu H. Choi S. Ma J. MacCready J. S.. (2021). Plant and fungal genome editing to enhance plant disease resistance using the CRISPR/Cas9 system. Front. Plant Sci. 12. doi: 10.3389/fpls.2021.700925 PMC8382960 34447401

[B152] Peters Haugrud A. R. Zhang Z. Friesen T. L. Faris J. D. (2022). Genetics of resistance to septoria nodorum blotch in wheat. Theor. Appl. Genet. 135, 3685–3707. doi: 10.1007/s00122-022-04036-9 35050394

[B153] Phan H. T. T. Furuki E. Hunziker L. Rybak K. Tan K. C. (2021). GWAS analysis reveals distinct pathogenicity profiles of Australian *Parastagonospora nodorum* isolates and identification of marker-trait-associations to septoria nodorum blotch. Sci. Rep. 11, 10085. doi: 10.1038/s41598-021-87829-0 33980869 PMC8115087

[B154] Phan H. T. T. Rybak K. Bertazzoni S. Furuki E. Dinglasan E. Hickey L. T.. (2018). Novel sources of resistance to septoria nodorum blotch in the vavilov wheat collection identified by genome-wide association studies. Theor. Appl. Genet. 131, 1223–1238. doi: 10.1007/s00122-018-3073-y 29470621 PMC5945755

[B155] Phan H. T. T. Rybak K. Furuki E. Breen S. Solomon P. S. Oliver R. P.. (2016). Differential effector gene expression underpins epistasis in a plant fungal disease. Plant J. 87, 343–354. doi: 10.1111/tpj.13203 27133896 PMC5053286

[B156] Piaskowska D. Piechota U. Radecka-Janusik M. Czembor P. (2021). QTL mapping of seedling and adult plant resistance to septoria tritici blotch in winter wheat cv. mandub (*Triticum aestivum* l.). Agronomy 11, 1108. doi: 10.3390/agronomy11061108

[B157] Plissonneau C. Hartmann F. E. Croll D. (2018). Pangenome analyses of the wheat pathogen *Zymoseptoria tritici* reveal the structural basis of a highly plastic eukaryotic genome. BMC Biol. 16, 5. doi: 10.1186/s12915-017-0457-4 29325559 PMC5765654

[B158] Rahman M. Davies P. Bansal U. Pasam R. Hayden M. Trethowan R. (2020). Marker-assisted recurrent selection improves the crown rot resistance of bread wheat. Mol. Breed. 40, 28. doi: 10.1007/s11032-020-1105-1

[B159] Rajarammohan S. (2021). Redefining plant-necrotroph interactions: the thin line between hemibiotrophs and necrotrophs. Front. Microbiol. 12. doi: 10.3389/fmicb.2021.673518 PMC8113614 33995337

[B160] Rawlinson C. See P. T. Moolhuijzen P. Li H. Moffat C. S. Chooi Y. H.. (2019). The identification and deletion of the polyketide synthase-nonribosomal peptide synthase gene responsible for the production of the phytotoxic triticone A/B in the wheat fungal pathogen *Pyrenophora tritici*-*repentis* . Environ. Microbiol. 21, 4875–4886. doi: 10.1111/1462-2920.14854 31698543 PMC6915911

[B161] Reddy L. Friesen T. L. Meinhardt S. W. Chao S. Faris J. D. (2008). Genomic analysis of the Snn1 locus on wheat chromosome arm 1BS and the identification of candidate genes. Plant Genome 1, 55–66. doi: 10.3835/plantgenome2008.03.0181

[B162] Rees R. G. Platz G. J. Mayer R. J. (1982). Yield losses in wheat from yellow spot: comparison of estimates derived from single tillers and plots. Aus. J. Agric. Res. 33, 899–908. doi: 10.1071/AR9820899

[B163] Reszka E. Song Q. Arseniuk E. Cregan P. B. Ueng P. P. (2007). The QTL controlling partial resistance to stagonospora nodorum blotch disease in winter triticale bogo. Plant Pathol. Bull. 16, 161–167.

[B164] Riaz A. KockAppelgren P. Hehir J. G. Kang J. Meade F. Cockram J.. (2020). Genetic analysis using a multi-parent wheat population identifies novel sources of septoria tritici blotch resistance. Genes 11, 887. doi: 10.3390/genes11080887 32759792 PMC7465482

[B165] Richards J. K. Kariyawasam G. K. Seneviratne S. Wyatt N. A. Xu S. S. Liu Z.. (2021). A triple threat: the *Parastagonospora nodorum* SnTox267 effector exploits three distinct host genetic factors to cause disease in wheat. New Phytol. 233, 427–442. doi: 10.1111/nph.17601 34227112 PMC9292537

[B166] Richards J. K. Stukenbrock E. H. Carpenter J. Liu Z. Cowger C. Faris J. D.. (2019). Local adaptation drives the diversification of effectors in the fungal wheat pathogen *Parastagonospora nodorum* in the united states. PloS Genet. 15, e1008223. doi: 10.1371/journal.pgen.1008223 31626626 PMC6821140

[B167] Richards J. K. Wyatt N. A. Liu Z. Faris J. D. Friesen T. L. (2018). Reference quality genome assemblies of three *Parastagonospora nodorum* isolates differing in virulence on wheat. G3 8, 393–399. doi: 10.1534/g3.117.300462 29233913 PMC5919747

[B168] Romdhane S. B. M. B. (2011). Genome structure and pathogenicity of the fungal wheat pathogen mycosphaerella graminicola. [PhD thesis] (Wageningen: Wageningen University).

[B169] Rudd J. J. Kanyuka K. Hassani-Pak K. Derbyshire M. Andongabo A. Devonshire J.. (2015). Transcriptome and metabolite profiling of the infection cycle of *Zymoseptoria tritici* on wheat reveals a biphasic interaction with plant immunity involving differential pathogen chromosomal contributions and a variation on the hemibiotrophic lifestyle definition. Plant Physiol. 167, 1158–1185. doi: 10.1104/pp.114.255927 25596183 PMC4348787

[B170] Ruud A. K. Dieseth J. A. Ficke A. Furuki E. Phan H. T. T. Oliver R. P.. (2019). Genome-wide association mapping of resistance to septoria nodorum leaf blotch in a Nordic spring wheat collection. Plant Genome 12, 1–15. doi: 10.3835/plantgenome2018.12.0105 PMC12810026 33016591

[B171] Ruud A. K. Dieseth J. A. Lillemo M. (2018). Effects of three *Parastagonospora nodorum* necrotrophic effectors on spring wheat under Norwegian field conditions. Crop Sci. 58, 159–168. doi: 10.2135/cropsci2017.05.0281

[B172] Ruud A. K. Lillemo M. (2018). “Diseases affecting wheat: septoria nodorum blotch,” in Burleigh dodds series in agricultural science (Cambridge, UK: Burleigh Dodds Science Publishing Limited), 109–144. doi: 10.19103/AS.2018.0039.06

[B173] Ruud A. K. Windju S. Belova T. Friesen T. L. Lillemo M. (2017). Mapping of *SnTox3-Snn3* as a major determinant of field susceptibility to septoria nodorum leaf blotch in the SHA3/CBRD × naxos population. Theor. Appl. Genet. 130, 1361–1374. doi: 10.1007/s00122-017-2893-5 28365817

[B174] Saintenac C. Cambon F. Aouini L. Verstappen E. Ghaffary S. M. T. Poucet T.. (2021). A wheat cysteine-rich receptor-like kinase confers broad-spectrum resistance against septoria tritici blotch. Nat. Commun. 12, 433. doi: 10.1038/s41467-020-20685-0 33469010 PMC7815785

[B175] Saintenac C. Lee W. S. Cambon F. Rudd J. J. King R. C. Marande W.. (2018). Wheat receptor-kinase-like protein *STB6* controls gene-for-gene resistance to fungal pathogen *Zymoseptoria tritici* . Nat. Genet. 50, 368–374. doi: 10.1038/s41588-018-0051-x 29434355

[B176] Sanchez-Vallet A. McDonald M. C. Solomon P. S. McDonald B. A. (2015). Is *Zymoseptoria tritici* a hemibiotroph? Fungal Genet. Biol. 79, 29–32. doi: 10.1016/j.fgb.2015.04.001 26092787

[B177] Sasaki A. (2000). Host–parasite coevolution in a multilocus gene-for-gene system. Proc. R. Soc Lond. B. 267, 2183–2188. doi: 10.1098/rspb.2000.1267 PMC1690804 11413631

[B178] Scharen A. L. Eyal Z. Huffman M. D. Prescott J. M. (1985). The distribution and frequency of virulence genes in geographically separated populations of *Leptosphaeria nodorum* . Phytopathology 75, 1463–1468. doi: 10.1094/Phyto-75-1463

[B179] Schnurbusch T. Paillard S. Fossati D. Messmer M. Schachermayr G. Winzeler M.. (2003). Detection of QTLs for stagonospora glume blotch resistance in Swiss winter wheat. Theor. Appl. Genet. 107, 1226–1234. doi: 10.1007/s00122-003-1372-3 12928778

[B180] Schroter J. (1894). Kryptogamen-flora von schlesien; im namen der schlesischen gesellschaft fur vaterlandische cultur Vol. 3–2. Ed. Cohn K.-F. (Polland: J.U. Kern's verlag), 340.

[B181] See P. T. Marathamuthu K. A. Iagallo E. M. Oliver R. P. Moffat C. S. (2018). Evaluating the importance of the tan spot ToxA*-Tsn1* interaction in Australian wheat varieties. Plant Pathol. 67, 1066–1075. doi: 10.1111/ppa.12835

[B182] Shankar M. Reeves K. Bradley J. Varischetti R. Loughman R. (2021). Effect of varietal resistance on the yield loss function of wheat to nodorum blotch. Plant Pathol. 70, 745–759. doi: 10.1111/ppa.13317

[B183] Shankar M. Walker E. Golzar H. Loughman R. Wilson R. E. Francki M. G. (2008). Quantitative trait loci for seedling and adult plant resistance to stagonospora nodorum in wheat. Phytopathology 98, 886–893. doi: 10.1094/PHYTO-98-8-0886 18943206

[B184] Shatalina M. Messmer M. Feuillet C. Mascher F. Paux E. Choulet F.. (2014). High-resolution analysis of a QTL for resistance to stagonospora nodorum glume blotch in wheat reveals presence of two distinct resistance loci in the target interval. Theor. Appl. Genet. 127, 573–586. doi: 10.1007/s00122-013-2240-4 24306318

[B185] Shetty N. P. Jensen J. D. Knudsen A. Finnie C. Geshi N. Blennow A.. (2009). Effects of β-1,3-glucan from *Septoria tritici* on structural defence responses in wheat. J. Exp. Bot. 60, 4287–4300. doi: 10.1093/jxb/erp269 19880540

[B186] Shi G. Zhang Z. Friesen T. L. Bansal U. Cloutier S. Wicker T.. (2016a). Marker development, saturation mapping, and high-resolution mapping of the septoria nodorum blotch susceptibility gene *Snn3-B1* in wheat. Mol. Genet. Genomics 291, 107–119. doi: 10.1007/s00438-015-1091-x 26187026

[B187] Shi G. Zhang Z. Friesen T. L. Raats D. Fahima T. Brueggeman R. S.. (2016b). The hijacking of a receptor kinase-driven pathway by a wheat fungal pathogen leads to disease. Sci. Adv. 2, e1600822. doi: 10.1126/sciadv.1600822 27819043 PMC5091353

[B188] Shoemaker R. A. (1959). Nomenclature of *Drechslera* and *Bipolaris*, grass parasites segregated from “*Helminthosporium*”. Can. J. Bot. 37, 879–887. doi: 10.1139/b59-073

[B189] Shoemaker R. A. (1962). *Drechslera* Ito. Can. J. Bot. 40, 809–836. doi: 10.1139/b62-075

[B190] Singh S. Bockus W. W. Sharma I. Bowden R. L. (2008). A novel source of resistance in wheat to *Pyrenophora tritici*-*repentis* race 1. Plant Dis. 92, 91–95. doi: 10.1094/PDIS-92-1-0091 30786378

[B191] Singh S. Hernandez M. V. Crossa J. Singh P. K. Bains N. S. Singh K.. (2012). Multi-trait and multi-environment QTL analyses for resistance to wheat diseases. PloS One 7, e38008. doi: 10.1371/journal.pone.0038008 22679489 PMC3367963

[B192] Singh P. K. Singh S. Deng Z. He X. Kehel Z. Singh R. P. (2019). Characterization of QTLs for seedling resistance to tan spot and septoria nodorum blotch in the PBW343/Kenya nyangumi wheat recombinant inbred lines population. Int. J. Mol. Sci. 20, 5432. doi: 10.3390/ijms20215432 31683619 PMC6862150

[B193] Sperschneider J. Dodds P. N. (2022). EffectorP 3.0: prediction of apoplastic and cytoplasmic effectors in fungi and oomycetes. Mol. Plant-Microbe Interact. 35 (2), 146–156. doi: 10.1094/MPMI-08-21-0201-R 34698534

[B194] Sprague R. (1950). Diseases of cereals and grasses in north America (New York: The Ronald Press).

[B195] Stadlmeier M. Jørgensen L. N. Corsi B. Cockram J. Hartl L. Mohler V. (2019). Genetic dissection of resistance to the three fungal plant pathogens *Blumeria graminis*, *Zymoseptoria tritici*, and *Pyrenophora tritici*-*repentis* using a multiparental winter wheat population. G3 9, 1745–1757. doi: 10.1534/g3.119.400068 30902891 PMC6505172

[B196] Stewart E. L. Croll D. Lendenmann M. H. Sanchez-Vallet A. Hartmann F. E., Palma-Guerrero. (2018). Quantitative trait locus mapping reveals complex genetic architecture of quantitative virulence in the wheat pathogen *Zymoseptoria tritici* . Mol. Plant Pathol. 19, 201–216. doi: 10.1111/mpp.12515 27868326 PMC6638037

[B197] Strelkov S. E. Lamari L. Ballance G. M. (1999). Characterization of a host-specific protein toxin (Ptr ToxB) from *Pyrenophora tritici*-*repentis* . Mol. Plant Microbe Interact. 12, 728–732. doi: 10.1094/MPMI.1999.12.8.728

[B198] Stukenbrock E. H. Banke S. McDonald B. A. (2006). Global migration patterns in the fungal wheat pathogen *Phaeosphaeria nodorum* . Mol. Ecol. 15, 2895–2904. doi: 10.1111/j.1365-294X.2006.02986.x 16911209

[B199] Stukenbrock E. H. Banke S. Zala M. Mcdonald B. A. Oliver R. P. (2005). Isolation and characterization of EST-derived microsatellite loci from the fungal wheat pathogen *Phaeosphaeria nodorum* . Mol. Ecol. Notes 5, 931–933. doi: 10.1111/j.1471-8286.2005.01120.x

[B200] Stukenbrock E. H. Jorgensen F. G. Zala M. Hansen T. T. McDonald B. A. Schierup M. H. (2010). Whole-genome and chromosome evolution associated with host adaptation and speciation of the wheat pathogen *Mycosphaerella graminicola* . PloS Genet. 6, e1001189. doi: 10.1371/journal.pgen.1001189 21203495 PMC3009667

[B201] Syme R. A. Hane J. K. Friesen T. L. Oliver R. P. (2013). Resequencing and comparative genomics of *Stagonospora nodorum*: sectional gene absence and effector discovery. G3 3, 959–969. doi: 10.1534/g3.112.004994 23589517 PMC3689807

[B202] Syme R. A. Tan K. C. Hane J. K. Dodhia K. Stoll T. Hastie M.. (2016). Comprehensive annotation of the *Parastagonospora nodorum* reference genome using next-generation genomics, transcriptomics and proteogenomics. PloS One 11, e0147221. doi: 10.1371/journal.pone.0147221 26840125 PMC4739733

[B203] Syme R. A. Tan K. C. Rybak K. Friesen T. L. McDonald B. A. Oliver R. P.. (2018). Pan-*Parastagonospora* comparative genome analysis-effector prediction and genome evolution. Genome Biol. Evol. 10, 2443–2457. doi: 10.1093/gbe/evy192 30184068 PMC6152946

[B204] Tamburic-Ilincic L. Rosa S. B. (2019). QTL mapping of fusarium head blight and septoria tritici blotch in an elite hard red winter wheat population. Mol. Breed. 39, 1–15. doi: 10.1007/s11032-019-0999-y

[B205] Tan K. C. Phan H. T. Rybak K. John E. Chooi Y. H. Solomon P. S.. (2015). Functional redundancy of necrotrophic effectors-consequences for exploitation for breeding. Front. Plant Sci. 6. doi: 10.3389/fpls.2015.00501 PMC4495316 26217355

[B206] Tan K. C. Waters O. D. C. Rybak K. Antoni E. Furuki E. Oliver R. P. (2014). Sensitivity to three *Parastagonospora nodorum* necrotrophic effectors in current Australian wheat cultivars and the presence of further fungal effectors. Crop Pasture Sci. 65, 150–158. doi: 10.1071/CP13443

[B207] Tian H. MacKenzie C. I. Rodriguez-Moreno L. van den Berg G. C. M. Chen H. Rudd J. J.. (2021). Three LysM effectors of *Zymoseptoria tritici* collectively disarm chitin-triggered plant immunity. Mol. Plant Pathol. 22, 683–693. doi: 10.1111/mpp.13055 33797163 PMC8126183

[B208] Tomas A. Feng G. H. Reeck G. R. Bockus W. W. Leach J. E. (1990). Purification of a cultivar-specific toxin from *Pyrenophora tritici*-*repentis*, causal agent of tan spot of wheat. Mol. Plant Microbe Interact. 3, 221–224. doi: 10.1094/MPMI-3-221

[B209] Tuori R. P. Wolpert T. J. Ciuffetti L. M. (1995). Purification and immunological characterization of toxic components from cultures of *Pyrenophora tritici*-*repentis* . Mol. Plant Microbe Interact. 8, 41–48. doi: 10.1094/MPMI-8-0041 7772802

[B210] Tyagi S. Kumar R. Das A. Won S. Y. Shukla P. (2020). CRISPR-Cas9 system: a genome-editing tool with endless possibilities. J. Biotechnol. 319, 36–53. doi: 10.1016/j.jbiotec.2020.05.008 32446977

[B211] Uphaus J. Walker E. Shankar M. Golzar H. Loughman R. Francki M.. (2007). Quantitative trait loci identified for resistance to stagonospora glume blotch in wheat in the USA and Australia. Crop Sci. 47, 1813–1822. doi: 10.2135/cropsci2006.11.0732

[B212] Van Der Biezen E. A. Jones J. D. (1998). Plant disease-resistance proteins and the gene-for-gene concept. Trends Biochem. Sci. 23, 454–456. doi: 10.1016/S0968-0004(98)01311-5 9868361

[B213] Vleeshouwers V. G. Oliver R. P. (2014). Effectors as tools in disease resistance breeding against biotrophic, hemibiotrophic, and necrotrophic plant pathogens. Mol. Plant Microbe Interact. 27, 196–206. doi: 10.1094/MPMI-10-13-0313-IA 24405032

[B214] Wang Y. Cheng X. Shan Q. Zhang Y. Liu J. Gao C.. (2014). Simultaneous editing of three homoeoalleles in hexaploid bread wheat confers heritable resistance to powdery mildew. Nat. Biotechnol. 32, 947–951. doi: 10.1038/nbt.2969 25038773

[B215] Weber G. F. (1922). Septoria diseases of cereals. II. septoria diseases of wheat. Phytopathology 12, 558–585.

[B216] Wicki W. Winzeler M. Schmid J. E. Stamp P. Messmer M. (1999). Inheritance of resistance to leaf and glume blotch caused by *Septoria nodorum* berk. in winter wheat. Theor. Appl. Genet. 99, 1265–1272. doi: 10.1007/s001220051332

[B217] Wu L. He X. Lozano N. Zhang X. Singh P. K. (2021). *ToxA*, a significant virulence factor involved in wheat spot blotch disease, exists in the Mexican population of *Bipolaris sorokiniana* . Trop. Plant Pathol. 46, 201–206. doi: 10.1007/s40858-020-00391-4

[B218] Xu S. S. Friesen T. L. Mujeeb-Kazi A. (2004). Seedling resistance to tan spot and stagonospora nodorum blotch in synthetic hexaploid wheats. Crop Sci. 44, 2238–2245. doi: 10.2135/cropsci2004.2238

[B219] Zaim M. Kabbaj H. Kehel Z. Gorjanc G. Filali-Maltouf A. Belkadi B.. (2020). Combining QTL analysis and genomic predictions for four durum wheat populations under drought conditions. Front. Genet. 11. doi: 10.3389/fgene.2020.00316 PMC7218065 32435259

[B220] Zhang F. (2022). Zt3LysM: a key effector protein in the fungal plant pathogen *Zymoseptoria tritici* . Imp. Biol. Sci. Rev.

[B221] Zhang Y. Bai Y. Wu G. Zou S. Chen Y. Gao C.. (2017). Simultaneous modification of three homoeologs of TaEDR1 by genome editing enhances powdery mildew resistance in wheat. Plant J. 91, 714–724. doi: 10.1111/tpj.13599 28502081

[B222] Zhang H. F. Francl L. J. Jordahl J. G. Meinhardt S. W. (1997). Structural and physical properties of a necrosis-inducing toxin from *Pyrenophora tritici*-*repentis* . Phytopathology 87, 154–160. doi: 10.1094/PHYTO.1997.87.2.154 18945135

[B223] Zhang Z. Friesen T. L. Simons K. J. Xu S. S. Faris J. D. (2009). Development, identification, and validation of markers for marker-assisted selection against the *Stagonospora nodorum* toxin sensitivity genes *Tsn1* and *Snn2* in wheat. Mol. Breed. 23, 35–49. doi: 10.1007/s11032-008-9211-5

[B224] Zhang P. Guo G. Wu Q. Chen Y. Xie J. Lu P.. (2020). Identification and fine mapping of spot blotch (*Bipolaris sorokiniana*) resistance gene *Sb4* in wheat. Theor. Appl. Genet. 133, 2451–2459. doi: 10.1007/s00122-020-03610-3 32451599

[B225] Zhang Z. Running K. L. D. Seneviratne S. Peters Haugrud A. R. Szabo-Hever A. Shi G.. (2021). A protein kinase-major sperm protein gene hijacked by a necrotrophic fungal pathogen triggers disease susceptibility in wheat. Plant J. 106, 720–732. doi: 10.1111/tpj.15194 33576059

[B226] Zhong Z. Marcel T. C. Hartmann F. E. Ma X. Plissonneau C. Zala M.. (2017). A small secreted protein in *Zymoseptoria tritici* is responsible for avirulence on wheat cultivars carrying the *Stb6* resistance gene. New Phytol. 214, 619–631. doi: 10.1111/nph.14434 28164301

[B227] Zhou B. Benbow H. R. Brennan C. J. Arunachalam C. Karki S. J. Mullins E.. (2020). Wheat encodes small, secreted proteins that contribute to resistance to septoria tritici blotch. Front. Genet. 11. doi: 10.3389/fgene.2020.00469 PMC7235427 32477410

[B228] Zwart R. S. Thompson J. P. Milgate A. W. Bansal U. K. Williamson P. M. Raman H.. (2010). QTL mapping of multiple foliar disease and root-lesion nematode resistances in wheat. Mol. Breed. 26, 107–124. doi: 10.1007/s11032-009-9381-9

