# Supplementary material for: Genetics and breeding for resistance against four leaf spot diseases in wheat (Triticum aestivum L.)
Source: Front Plant Sci. 2023 Mar 29;14:1023824. doi: 10.3389/fpls.2023.1023824 (PMC10096043; doi:10.3389/fpls.2023.1023824)
Supplement: Supplementary file 1 [file DataSheet_1.docx]

| **Supplementary Table S1\|** Sensitivity genes, and R genes for four wheat leaf spot diseases; |
| --- |
| I. **Septoria nodorum blotch (SNB):** |
| **Genes/QTLs with Culture filtrate and Purified NEs** |
| **Sensitivity genes (10):** |
| *Tsn1*, *Snn1-Snn3, Snn3-B1, Snn3-D1, Snn4-7;* (Sn15, BBCSn5 and Sn2000, Sn6, Sn1501, Sn4,); (SnToxA, SnTox1, SnTox2, SnTox) |
| **References:** Abeysekara et al., 2012; Friesen et al., 2006, 2007, 2008, 2009, 2012; Gao et al. 2015; Liu et al., 2004a, 2004b, 2006, 2009; Shi et al., 2015; Zhang et al., 2009, 2011; Faris and Friesen 2009; Faris et al., 2010; Reddy et al., 2008; Cockram et al., 2015 |
| **R genes (3)** |
| *SnbTM; SnbAes1; Snb1, Snb2* and *Snb3* |
| **References:** *Feng et al., 2004; McIntosh et al., 2007, 2008* |
| **II. Tan spot (TS)** |
| **Sensitivity genes (3):** |
| *Tsn1, Tsc1, Tsc2;* (78-62, 86-124 and ASC1, Ptr race 5 and Shuffle strain); (Ptr ToxA, Ptr ToxB, Ptr ToxC) |
| **References:** Abeysekara et al., 2010; Corsi et al., 2020; Effertz et al., 2002; Faris et al., 1996, 1997, 1999; Friesen and Faris, 2004; Kariyawasam, 2018; Lamari et al., 1991; Orolaza et al., 1995; Stock et al., 1996 |
| **R genes (10):** |
| *Tsr1 to Tsr7, TsrHar, TsrAri, TsrAes1* |
| **References:** Faris et al., 1996, 2020; Friesen and Faris, 2004; Singh et al., 2006, 2008b Tadesse et al., 2006a, 2006b, 2007, 2010, Zhang et al., 2019 |
| **III. Spot Blotch (SB)** |
| **Sensitivity gene (1):** |
| *Tsn1;* (BRIP10943 and mixture of 15 isolates); (Bs ToxA) |
| **References:** Friesen et al., 2018; McDonald et al., 2018 |
| **R genes (4):** |
| *Sb1 to Sb4* |
| **References:** Kumar et al., 2015; Lillemo et al., 2013; Lu et al., 2016; Zhang et al., 2020 |
| **IV. Septoria tritici blotch (STB)** |
| **Sensitivity gene: (None)** |
| **R genes (22):** |
| *Stb1 to Stb19, StbSm3, StbWW, TmStb* |
| **References:** Adhikari et al., 2003, 2004a, 2004b; Arraiano et al., 2001, 2007; Branding et al., 2002; Chartrain et al., 2005a, 2005b, 2009; Cuthbert, 2011; Cowling, 2006; Goodwin and Thompson, 2011; Jing et al., 2008; Liu et al., 2013; McCartney et al., 2003; Raman et al., 2009; Tabib Ghaffary et al., 2011, 2012; Yang et al., 2018 |

| **Supplementary Table S2\|** A summary of QTLs detected using interval mapping for four wheat leaf spot diseases |
| --- |
| **I. Septoria nodorum blotch (SNB):** |
| *QInf.nmbu5A.1, QInf.nmbu5B.1, QInf. nmbu.6A.1, QTox3. nmbu.6A. 1, QInf.nmbu.7B.1, QSnb.niab-5B.1 (Snn3-B1), QSnb.niab-5B.2 (Tsn1), QTox3.nmbu5B.1,* Anonymous QTL on *1AS, QSnn.niab-5A.1,* Anonymous QTLs on *1B and 5B, QTox3.niab-6A.1, QTox3.niab-7B.1, QTox3.niab-2A.1, QTox3.niab-2B.1,* Anonymous QTLs on *2DS, 4BL, 5BL, 5BS; QSnb.fcu-2DS, QSnb.fcu-5BL;* Anonymous QTL on *6AL, QSnb.niab-5B.2, QTox3.nmbu-5B.1, QInf.nmbu-5B.1, QInf.nmbu-6A.1, QTox3.nmbu-6A.1, QInf.nmbu-7B.1,* Anonymous QTLs on *1B, 5B, Q.snb.fcu-1BS, Qsnb.cur-2AS2, Qsnb.cur-2AS1, Qsnb.cur-2BS, Qsnb.cur-3AL, Qsnb.fcu-4BL, Qsnb.fcu-5BS,* Anonymous QTLs on *1B, 2D,* Anonymous QTL on *5B, QSnb.fcu-1A, Qsnb.fcu-5BL.1, Qsnb.fcu-1BS*, *Qsnb.fcu-5BS*, *Qsnb.cur-4BL*, *Qsnb.cur-2AS1;* Anonymous QTL on *1B, QSnl.ihar-6A, QSnl.eth-2D, QSng.pur-2DL.1,*  Anonymous QTL on *4B, QSnr.daw-5A,* Anonymous QTL on *5B, QSnb.fcu-2DS* and *QSnb.fcu-5BL.1 (tsn1), QSnl.daw-2A, QSnb.fcu-1AS, QSng.sfr-3BS,* Anonymous QTL on *5BS, QSnl07.daw-1B, QSnl.ihar-7D, QSnb.cim-5B.2, QSnb.niab-2A.3, QSnb.nmbu-2A.1, QSnb.nmbu-5A.1, QSnb.nmbu-2A.1*, *QSnb.nmbu-2A.1*, *QSnb.nmbu-2B.1*, *QSnb.nmbu-2D.1*, *QInf.nmbu-5A.1*, *QSnb.nmbu-5A.1*, *QSnb.nmbu-5A.1*, *QTox3.nmbu-5B.1*, *QInf.nmbu-5B.1*, *QTox3.nmbu.6A.1*, *QInf.nmbu.7B.1* |
| **References:** Abeysekara et al., 2009, 2012; Agulilar et al., 2005; Arseniuk et al., 2004; Cockram et al., 2015; Czembor et al., 2019; Downie et al., 2018; Francki et al., 2011, 2018; Friesen et al., 2009; Gao et al., 2015; Gonzalez-Hernandez et al., 2009; John et al., 2022; Lin et al. 2020a, 2020b, 2020c, 2021, 2022; Liu et al., 2004a, 2004b, 2015; Phan et al., 2016, 2018; Reszka et al., 2007, Shankar et al., 2008; Shatalina et al., 2014; Singh et al., 2019; Rudd et al., 2017; Uphaus et al., 2007 |
| **II. Tan spot (TS)** |
| *QTsc.ndsu-1AS, QTs.zhl-5B, 2A (Tsc2), 1A (Tsc1); QTsc.ndsu-1AS,* Anonymous QTLs on *2BS, 4AL, QTs.fcu-3BL, QTs.fcu-5A.1, QTs.cim-5BL, QTs.ksu-3AS*, *QTs.ksu-5BL, QTs.fcu-5BL.1, 3BL (tsn2*, *tsn5), QTs.ksu-2BS*, *QTs.ksu-1AS, QTs.zhl-1A, QTs.ksu-1A, QTs.zhl-3B*, *QTs.ksu-6A*, *QTs.zhl-5B; QTs.ksu-3AS,* Anonymous QTLs on *2BS,* Anonymous QTLs on *5B*, Qts.313-1A, Qts.313-2B, Qts.313-5B, Qts.313-5D, Qts.313-6A |
| **References:** Chu et al., 2008, 2010; Corsi et al., 2020; Dinglasan et al., 2021; Effertz et al., 2002; Faris et al., 1997; Friesen and Faris, 2004, 2005; Hu et al., 2019; Kalia et al., 2017; Kariyawasam, 2016, 2018; Li et al., 2011; Liu et al., 2017; Running et al., 2022; Singh S. et al., 2008; Singh et al., 2006, 2008a, 2008b, 2012; Sun et al., 2010 |
| **III. Spot Blotch (SB)** |
| *QSb.bhu-2B, QSb.bhu-5B*, *QSb.bhu-6D, QSb.bhu-2A*, *QSb.bhu-7D, QSb.cim-3B,* Anonymous QTL on *5A, QSb.pau-2B*, *QSb.pau-3B; QSb.bhu-2A, 7DS (Sb1), 7DS, 5BL (Sb2), 3BS (Sb3), 4BL* (*Sb4*)*,* Anonymous QTL on *5A, Qsb_rpcau_5B* |
| **References:** He et al., 2020; Kaur J. et al., 2021; Kumar et al., 2009, 2010, 2015; Lillemo et al., 2013; Lu et al., 2016; Pankaj et al., 2022; Singh et al., 2018, Zhang et al., 2020, Zhu et al., 2014 |
| **IV. Septoria tritici blotch (STB)** |
| *QStb.teagasc-1A.1, QStb.teagasc-1B.2, QStb.teagasc-1D.1, QStb.teagasc-2B.1, QStb.teagasc-2D.1, QStb.teagasc-3A.1, QStb.teagasc-4A.1, QStb.teagasc-4B.1 QStb.teagasc-4D.1, QStb.teagasc-5A.1, QStb.teagasc-5B.1, QStb.teagasc-6A.1, QStb.teagasc-6A.2, QStb.teagasc-6D.1*, *QStb.teagasc-7B.1,*  Anonymous QTLs on *2DS, 3AS, 6DS, 7DS,* Anonymous QTL on *3DL, QStb.ihar-3A.2, QStb.ihar-3A.2, QStb.ihar-3A.2, QStb.ihar-3A.2, QStb.ihar-1B, QStb.ihar-1B, QStb.ihar-5D, QStb.ihar-7A.2, QStb.ihar-7A.2, QStb.ihar-5D, QStb.ihar-7A.2*, *QStb.ihar-3A.2, QStb.wai-3D,*  Anonymous QTL on *6AS, QStb.jic2A;* QStb.ihar-2B.4, QStb.ihar-1B.2,  *Qstb2B_1, Qstb2B_2, Qstb.renan-5D, Qstb.renan-7B* |
| **References:** Arraiano et al., 2007; Cuthbert et al., 2011; Ferjaoui et al., 2022; He et al., 2021; Langlands-Perry et al., 2021; Piaskowska et al., 2021; Radecka-Janusik and Czembor, 2014; Riaz et al., 2020; Tabib Ghaffary et al., 2011; Zwart et al., 2010; |

| **Supplementary Table S3\|** A summary of MTAs detected using GWAS for the leaf spot diseases in wheat |
| --- |
| **I. Septoria nodorum blotch (SNB):** |
| *QSnb.sdsu-1B* and *QSnb.sdsu-2A, QSnb.sdsu-2D* and *QSnb.sdsu-4A, QSnb.sdsu-5B* and *QSnb.sdsu-6B, QSnb.sdsu-7A*, *SnToxA, SnTox3*  *BS00070979_51, Excalibur_c20478_641, BS00064483_51, Kukri_c1526_666,*  *BS00015136_51, GENE-3277_145, BobWhite_c13839_135, IACX7801, BS00022127_51, Kukri_c67849_109, Excalibur_c20478_641, Tdurum_contig56321_232,*  *wsnp_Ex_c11246_18191331, wsnp_Ex_c22401_31592784, BS00025784_51, BS00065732_51, BS00070979_51, Excalibur_c20478_641, wsnp_Ex_c11246_18191331,*  *wsnp_Ex_c22401_31592784, BS00025784_51, BS00065732_51* and *BobWhite_c13839_135, IACX7801*  *wsnp_Ex_c23239_32477458, wsnp_Ku_c9269_15583444, wsnp_BE426620D_Ta_2_2, wsnp_CAP11_c318_261649, wsnp_Ex_c5047_8963671, wsnp_Ku_c40334_48581010, wsnp_Ku_c2185_4218722, wsnp_CAP12_c2547_1227972, wsnp_Ex_c12220_19528388, wsnp_BF473744B_Ta_2_2, wsnp_Ex_rep_c67561_66189356, wsnp_Ex_c19772_28771627, wsnp_Ex_rep_c67159_65649966, wsnp_Ex_c9971_16412345, wsnp_Ex_c9971_16412270, wsnp_Ex_c9971_16412758, wsnp_Ku_c26118_36079171, wsnp_Ex_c2887_5330426, wsnp_Ex_c12354_19711297, wsnp_Ra_c44141_50623811, wsnp_Ex_c2920_5385184, wsnp_Ex_rep_c67468_66068960* and *wsnp_Ex_c17575_26301455*  *Q.Snb.sdsu-2BS, Q.Snb.sdsu-5AL, Q.Snb.sdsu-5BL, Q.Snb.sdsu-5BL* and *Q.Snb.sdsu-7AS*  *AX.94610974, AX.94457982, AX.95126753, AX.94978939, AX.94774467, AX.95165387, AX.94753171, AX.94828722, AX.94610974, AX.95126753, AX.95019005, AX.94681771, AX.94429024, AX.94531841, AX.94610974, AX.94457982, AX.95126753, AX.94407285, AX.94950274, AX.95253275, AX.95171319, AX.94610974, AX.95010548, AX.94457982, AX.95126753, AX.94459459, AX.94407285, AX.94535772, AX.94860625, AX.94935613, AX.95255981, AX.94526408, AX.94948233, AX.94428968, AX.94694976, AX.95632742, AX.94935613, AX.95133018, AX.94792356, AX.94610974, AX.95629214, AX.94436382, AX.94427355, AX.95007069, AX.94699167, AX.94575839, AX.94723571, AX.94954416, AX.94804165, AX.95097189, AX.94935613 AX.94772629, AX.95086097*  and *AX.94750259*  *Tdurum_contig12066_126, Tdurum_contig12066_247, BobWhite_c48435_165, tplb0027f13_1346, IACX9261, Tdurum_contig25513_123, Tdurum_contig25513_195, tplb0027f13_1493, wsnp_Ku_c40334_48581010, BS00010590_51, fcp620, fcp394, fcp394, fcp620, fcp1, fcp1, gwm234, cfd20, cfd20, Excalibur_c47452_183* and *BS00091519_51*  *QSnl.ihar-5B* and *QSnl.ihar-5D, QSng.sfr-3B*  *wPt_731148, wPt_1554, wPt_730744, wPt_671778, wPt_667406, wPt_665317, wPt_666332, wPt_9510, wPt_6047, wPt_1625, wPt_5175, wPt_1149, wPt_0963, wPt_666819, wPt_7330, wPt_729877, wPt_4835, wPt_4515* and *wPt_3992* |
| **References:** AlTameemi et al., 2021; Downie et al., 2018; Gurung et al., 2014; Halder et al., 2019; Phan et al., 2021; Ruud et al., 2019; Czembor et al., 2003; Schnurbusch et al., 2003; Adhikari et al., 2011 |
| **II. Tan spot (TS)** |
| *987556, 6045377, 1089962, 1070935, 4993454, 1200982, 4393896, 100034112, 1862737, 100027398, 5331622, 1254459, 4993056, 1019955, 991140* and *993425*  *QTs.ipk-7A.1, QTs.ipk-7A.1, QTs.ipk-7A.2, QTs.ipk-7A.2, QTs.ipk-7A.2, QTs.ipk-7A.3, QTs.ipk-7A.3, QTs.ipk-7A.3, QTs.ipk-7A.4, QTs.ipk-7A.4, QTs.ipk-7A.4, QTs.ipk-7A.4, QTs.ipk-7A.4, QTs.ipk-7A.4, QTs.ipk-7A.4, QTs.ipk-7B* and *QTs.ipk-7A*  *S2B_24100467, S2B_24099403, S2B_24099412, S2B_24099418, S5B_546810215, S2B_24356781, S2B_24210901, S5B_546780974, S5B_546781047, S2B_23971365, S5B_545943215, S2B_24354200, S5B_545940211, S5B_545943463* and *S5B_545812379*  *S1_3589926, S7_182028651, S4_239686345, S16_4196814, S8_7801088, S7_4804454, S16_191519837, S1_2331617, S7_4563676, S5_281016023* and *S1_2584791*  *wPt6427, wPt5065, wPt8986, wPt4434, wPt1328, wPt2614, wPt1781, wPt5363, wPt8616* and *wPt2019*  *WMC0522_200, WMC0522_200, WMC0522_200, BARC0204b_500, BARC0204b_500, GWM1276_205, GWM1276_205, GWM1391_160* and *GWM1391_160*  *BS00064197_51, Kukri_c23985_229, BobWhite_c4646_119, Excalibur_c47654_70, Excalibur_c77035_156, IACX5982, Kukri_c23985_166, Kukri_rep_c104386_273, RAC875_c5544_4156, Excalibur_c37642_1416, Ex_c13277_2025, Kukri_c17396_2448, Kukri_c54078_114, Kukri_c90424_72,Kukri_rep_c113115_261, Kukri_rep_c113115_424, Ra_c38583_333, wsnp_Ex_c13277_20936069, wsnp_Ku_c3102_5810751, wsnp_Ku_c3102_5811860, BS00010590_51, BobWhite_c48435_165, IACX9261,*  *Tdurum_contig25513_123, tplb0027f13_1493, tplb0027f13_452,*  *wsnp_Ku_c17396_26488733, wsnp_Ku_c40334_48581010, Excalibur_c23598_1632, Kukri_rep_c103261_918, Kukri_rep_c69177_180, RAC875_c2698_132, RAC875_c62831_255,* *Excalibur_c14396_1629, Kukri_c148_1484, wsnp_Ku_c9883_16462146, Kukri_c148_1346, Kukri_c148_1512, BS00010318_51,BS00070050_51, BS00070051_51, BS00072619_51, BS00072620_51, BS00075303_51, RAC875_c38003_164, wsnp_Ra_c4321_7860456, Ku_c63748_1264, RAC875_c19575_84, .wsnp_Ex_rep_c66551_64836327, Kukri_c45103_371, Excalibur_c1986_439, Excalibur_rep_c112367_293, GENE-1343_556, Kukri_c3067_398, BS00011149_51, IAAV5802,* SNP_66420*, *wsnp_Ex_c19371_28311667, wsnp_Ex_rep_c105401_89840110, IACX8278, BS00064274_51, BobWhite_c14360_420, Excalibur_c23661_1712, Excalibur_c32979_1152, GENE-2855_121, IAAV5779, IACX11346, Kukri_c11272_486, wsnp_Ex_c23661_32900048, wsnp_RFL_Contig3139_3096141, wsnp_RFL_Contig3238_3265410, BS00097105_51, Kukri_rep_c112425_506, Kukri_rep_c112425_98, RAC875_c6890_150,*  *Tdurum_contig8171_1548, Tdurum_contig8171_1712* and *Tdurum_contig8171_1772*  *Q.Ts1.sdsu-1AL, Q.Ts1.sdsu-1AL, Q.Ts1.sdsu-2BS, Q.Ts1.sdsu-3AS, Q.Ts1.sdsu-3BL, Q.Ts1.sdsu-4AL, Q.Ts1.sdsu-4BS, Q.Ts1.sdsu-5AL, Q.Ts1.sdsu-5BS, Q.Ts1.sdsu-5BL, Q.Ts5.sdsu-1BL, Q.Ts5.sdsu-2DL, Q.Ts5.sdsu-3AL, Q.Ts5.sdsu-5BL* and *Q.Ts5.sdsu-6BL* |
| **References:** Lozano-Ramirez et al., 2022a; Muqaddasi et al., 2021; Galagedara et al., 2020; Juliana et al., 2018; Singh et al., 2016; Kollers et al., 2013; Perez-Lara et al., 2017; Halder et al., 2019 |
| **III. Spot Blotch (SB)** |
| *4909825, 1085203, 1128070, 1220348, 7354241, 991620, 100177527, 5411867* and *998276*  *Q.Sb.bisa-1D, Q.Sb.bisa-2A, Q.Sb.bisa-2B, Q.Sb.bisa-4A, Q.Sb.bisa-5B* and *Q.Sb.bisa-6D*  *wsnp_Ex_c24700_33953160, wsnp_JD_c8926_9848514, wsnp_Ex_c15342_23592740, wsnp_Ku_c17951_27138894, wsnp_Ex_rep_c70120_69069789, wsnp_Ku_c50354_55979952, wsnp_Ku_c20701_30355248, wsnp_Ex_c15785_24157360* and *wsnp_Ex_c52527_56097039* |
| **References:** Bainsla et al., 2020; Tomar et al., 2020; Gurung et al., 2014 |
| **IV. Septoria tritici blotch (STB)** |
| RFL_Contig2834_890, wsnp_Ex_c790_1554988, Kukri_c6266_260,  BobWhite_c15773_166, wsnp_Ex_c14340_22315611, Excalibur_c15048_488,  RAC875_c8145_1201, wsnp_Ex_c12618_20079758, RAC875_c1357_860, RAC875_c1742_2710 and Excalibur_c72359_56  SNB_Tox1B, WB_BR43_2A, STB_Nec3A, LR_Mock2B and LR_Mock6A  Excalibur_c34649556  QTL3, wmc764 and wPt-4453  GWM1391_158, GWM1391_158, WMC0522_200, WMC0522_200, WMC0522_200, BARC0204b_500, BARC0204b_500, GWM1276_205, GWM1276_205, GWM1391_160 and GWM1391_160  wsnp_RFL_Contig4792_5787180, wsnp_CAP11_c59_99317, wsnp_CAP11_c59_99769, wsnp_Ex_c5744_10087758, wsnp_Ex_rep_c106072_90285324 and wsnp_JD_c646_966400 |
| **References:** Alemu et al., 2021; Balilini et al., 2020; Dutta et al., 2021; Goudemand et al., 2013; Koller et al., 2013; Gurung et al., 2014 |
