# Supplementary material for: Genetics and breeding for resistance against four leaf spot diseases in wheat (Triticum aestivum L.)
Source: Front Plant Sci. 2023 Mar 29;14:1023824. doi: 10.3389/fpls.2023.1023824 (PMC10096043; doi:10.3389/fpls.2023.1023824)
Supplement: Supplementary file 2 [file DataSheet_2.doc]

| 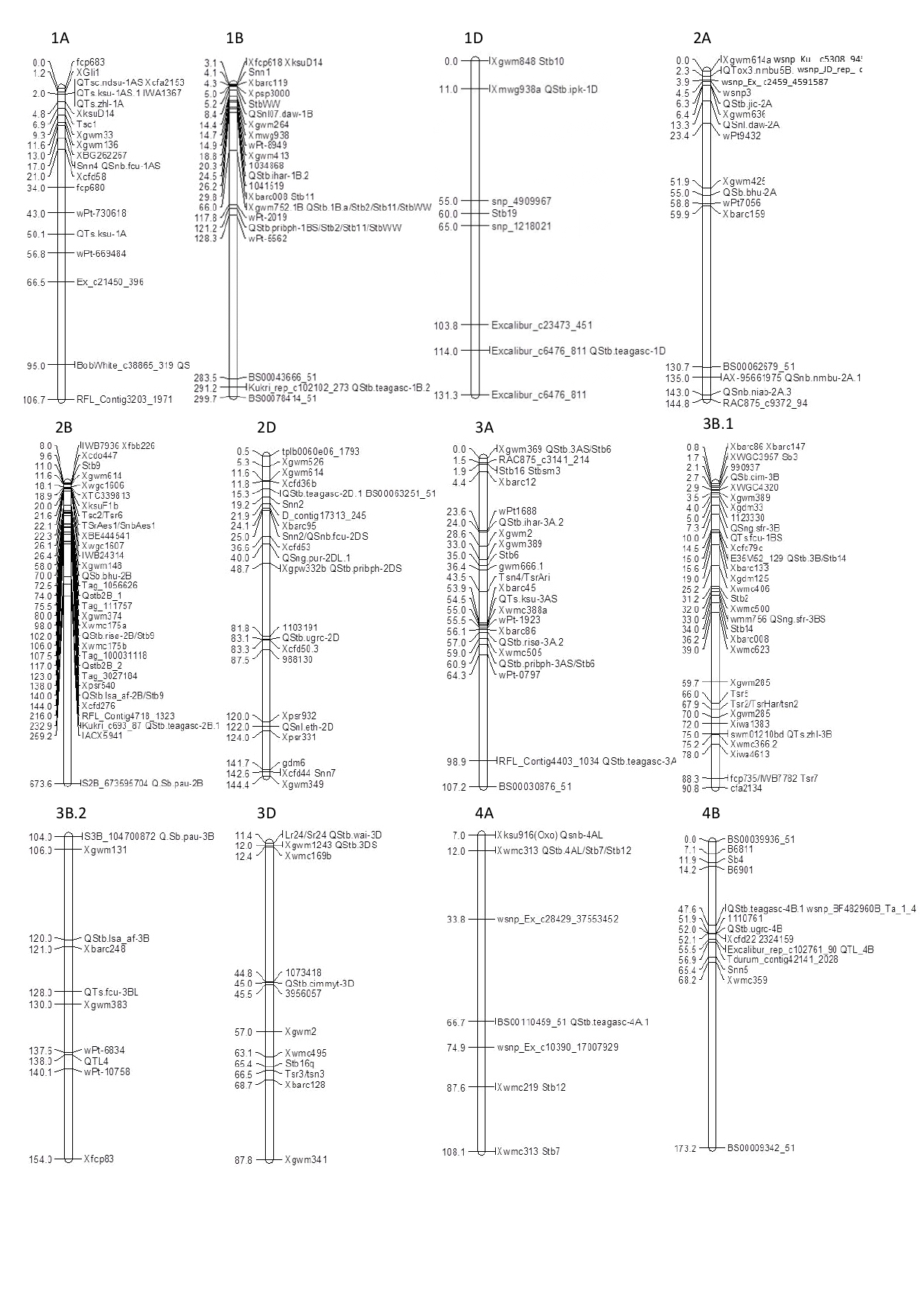 |
| --- |
| 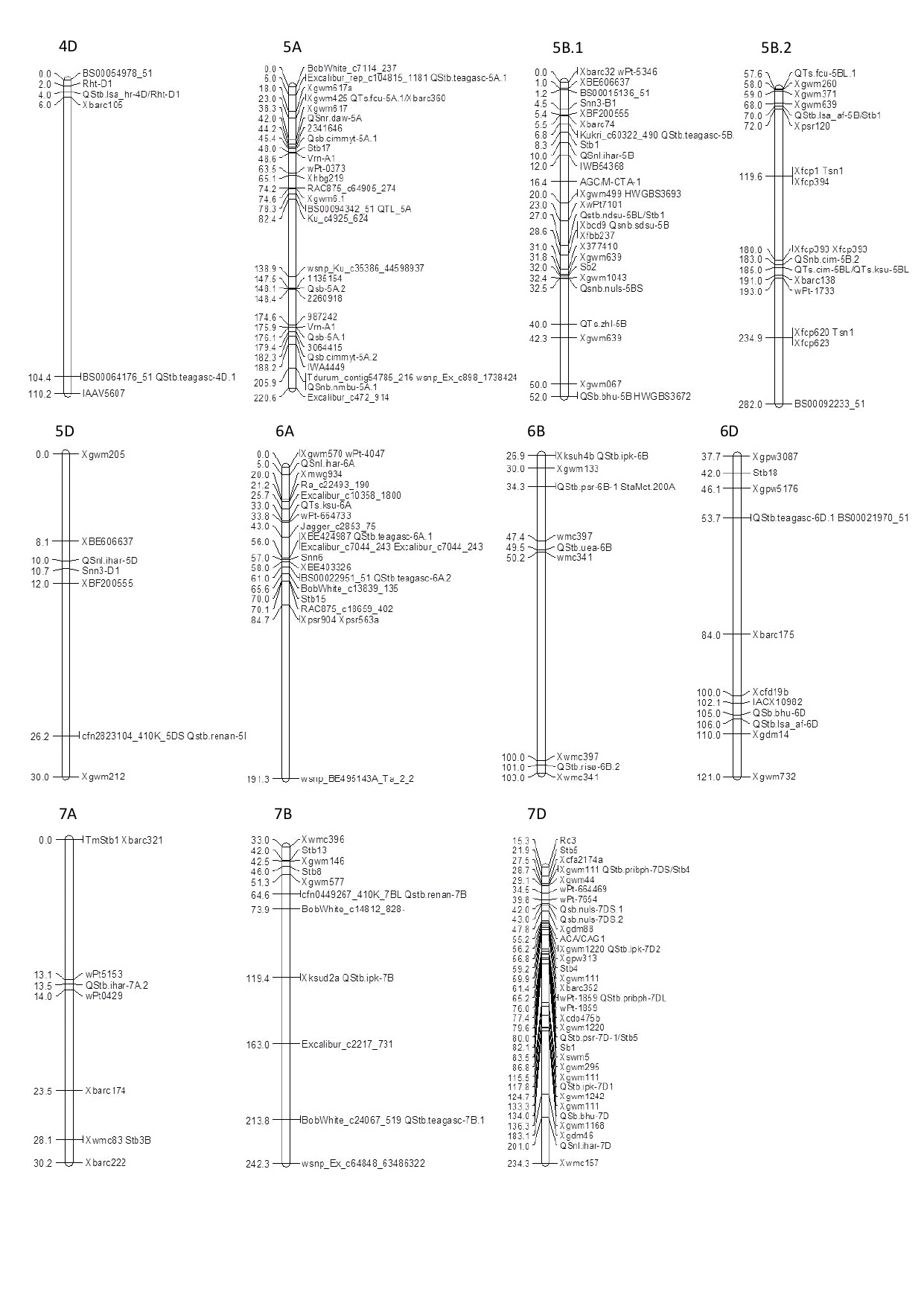 |
| **SUPPLEMENTARY FIGURE S1**| A graphical representation of chromosomal locations of sensitivity/susceptibility related genes/QTLs and resistance related genes/QTLs of SNB, TS, SB and STB with their associated molecular markers identified using either Mendelian approach or interval mapping (IM). |

|  |
| --- |
| 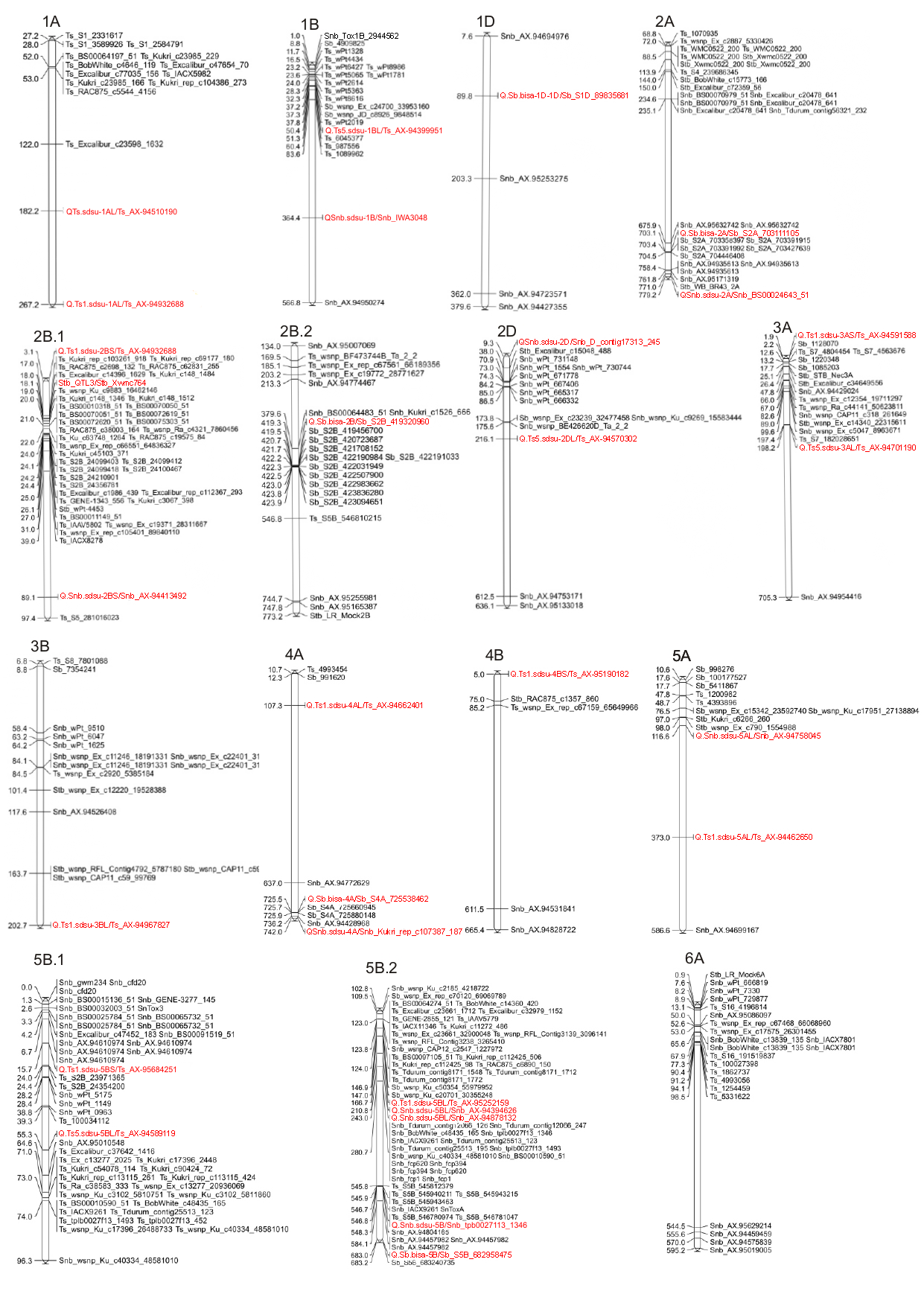 |
| 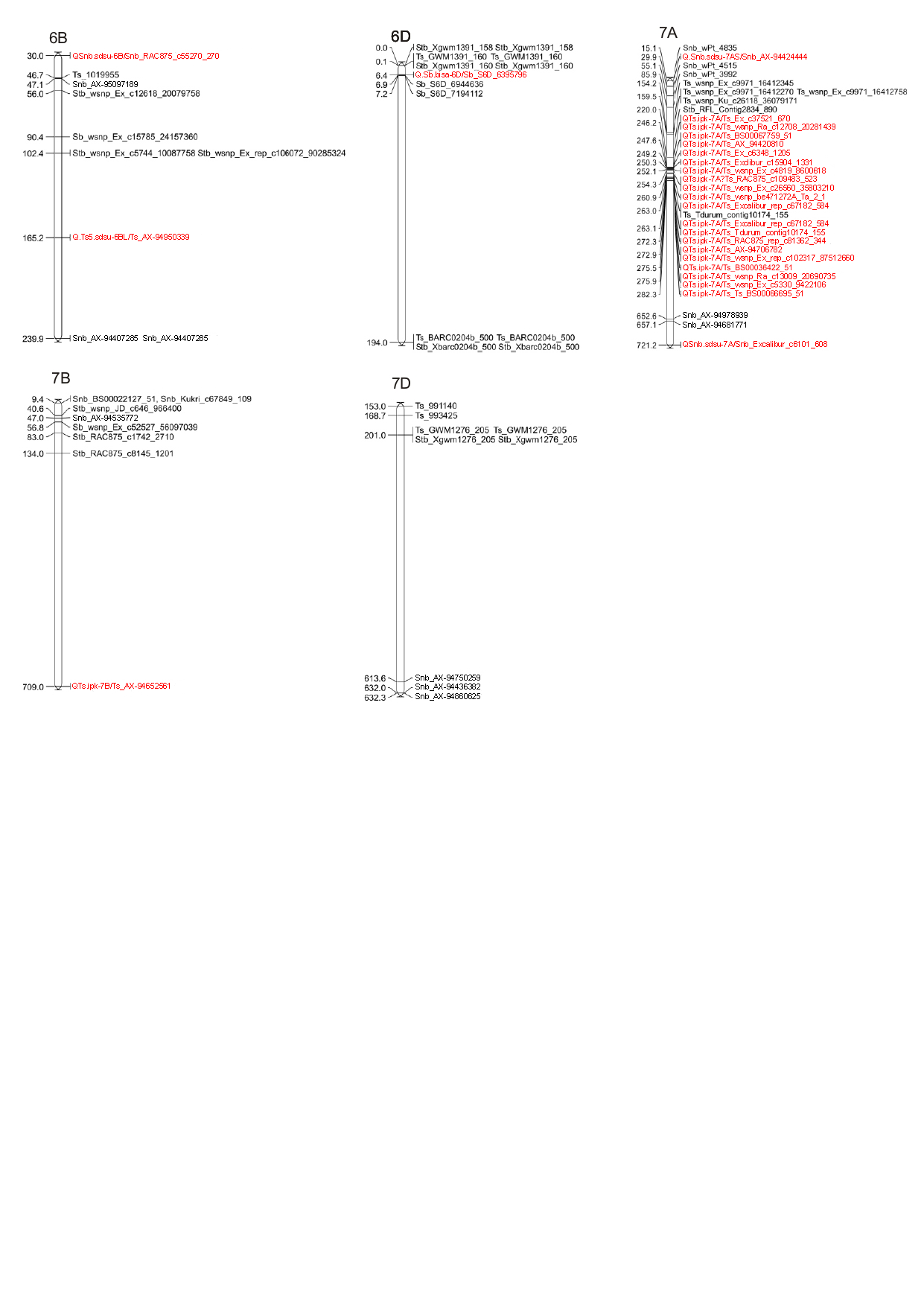 |
| **SUPPLEMENTARY FIGURE S2|** A graphical representation of chromosomal locations of MTAs/QTLs (in red) for SNB, TS, SB and STB with their associated molecular markers identified using GWAS approach. |

| 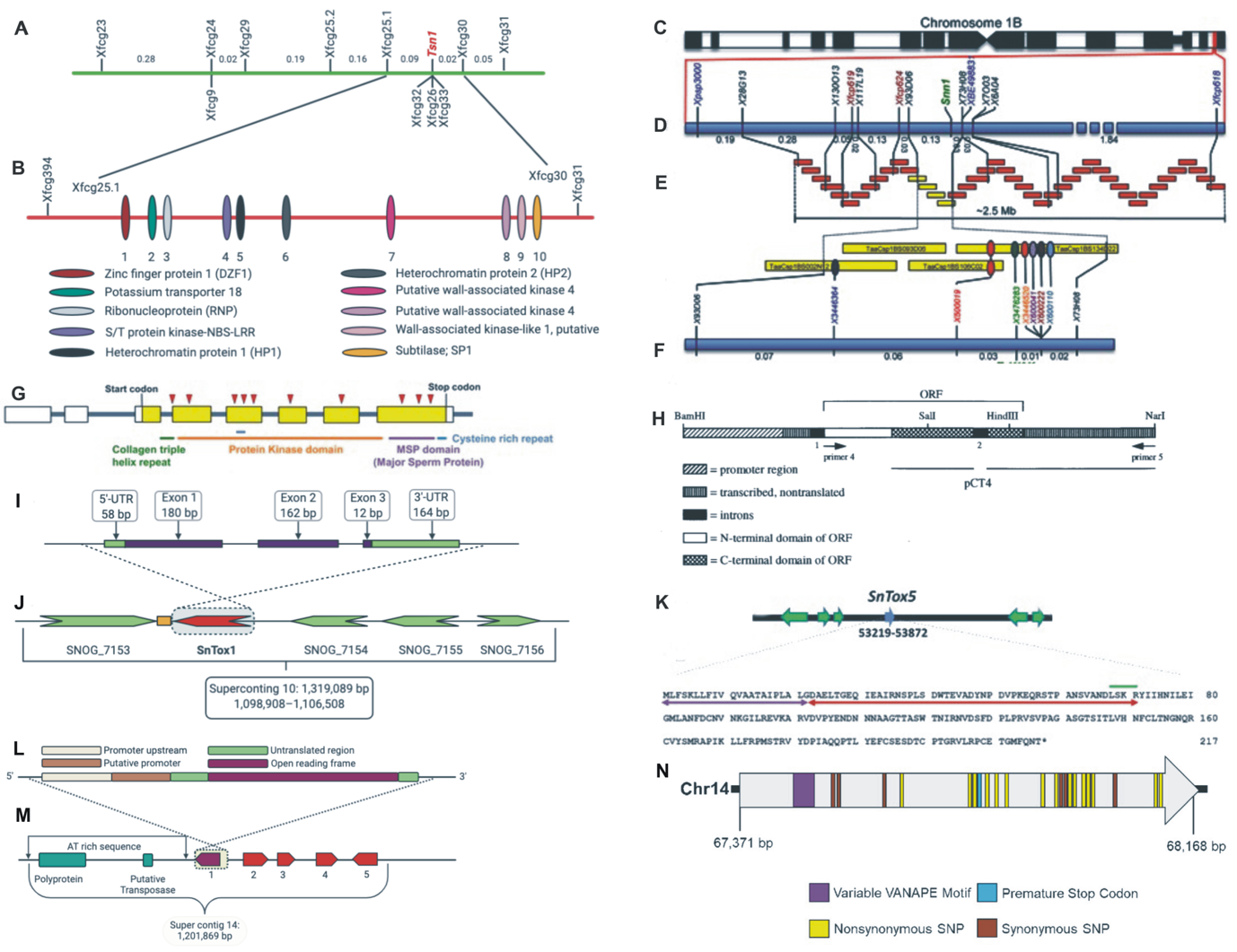 |
| --- |
| **SUPPLEMENTARY FIGURE S3|** Map-based cloning of three S genes *Tsn1*, *Snn1*, *Snn3-D1,* (wheat) and five NE genes *ToxA*, *SnTox1*, *SnTox5*, *SnTox3*, *SnTox267*. (*P. nodorum*)  A-B (*Tsn1*): **(A)** Genetic linkage map showing position of *Tsn1* gene. **(B)** Details of *Tsn1* genomic region with flanking markers, showing 10 genes (oval) (Based on Faris et al., 2010).  C-F (*Snn1*): **(C)** Chromosome 1B with *Snn1* gene (in red) located on the extreme right on 1BS. **(D)** The genetic linkage map of *Snn1* region, showing markers: green (Shi et al., 2016b); blue (Reddy et al., 2008) and red (unpublished work). **(E)** A physical map based on the BAC clones with *Snn1* region represented by four overlapping BAC clones (shown in yellow). **(F)** A linkage map with seven candidate genes with two flanking markers (Based on Shi et al., 2016b).  G (*Snn3-D1*): Structure of *Snn3-D1* gene with its predicted functional domains, exons (yellow), UTRs (white) and EMS induced muatations (red arrowheads) (Reproduced from Zhang et al., 2021).  H, I (*ToxA*): **(H)** Structure of *ToxA* locus with promoter region, ORF (534bp) and N/C terminal domains. **(I)** Structure of SnTox1 gene with 5′ and 3′ UTRs (green bars) and 3 exons (purple bars).  **J (*SnTox1*): Structure of** SnTox1 gene and associated genomic region with 4 other genes- *SNOG7153* to *SNOG7156* (boxed arrows)) and a retrotransposon (yellow rectangle). (Based on Liu et al., 2012).  K (*SnTox5*): Genomic location of *SnTox5* (isolate Sn2000) and the sequence of amino acid of SnTox5 protein; bars, purple = signal peptide, red = pro-sequence, green = putative Kex2 protease site, (*) = stop codon (Reproduced from Kariyawasam et al., 2021).  L, M **(***SnTox3***)**: **(L)** Structure of *SnTox3*, showing promoter, ORF and two UTRs; (B) position of *SnTox3* in super-contig 14 (based on Liu et al., 2009).  N (*SnTox267*): Model for genomic region carrying NE gene *SnTox267* (based on Richards et al., 2021). |

| 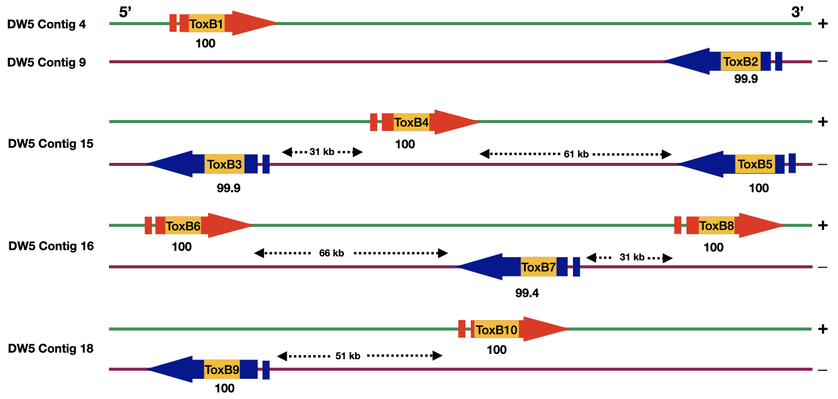 |
| --- |
| **SUPPLEMENTARY FIGURE S4|** Five different contigs of *P. tritici-repentis* genome showing positions of 10 different *ToxB* loci on forward and reverse strands. Blue arrows represent *Tox B* loci in the forward strand; red arrows show *ToxB* loci in the reverse strand. In each case, the coding sequence is shown in yellow. (Modified from Moolhuijzen et al., 2020) |

| 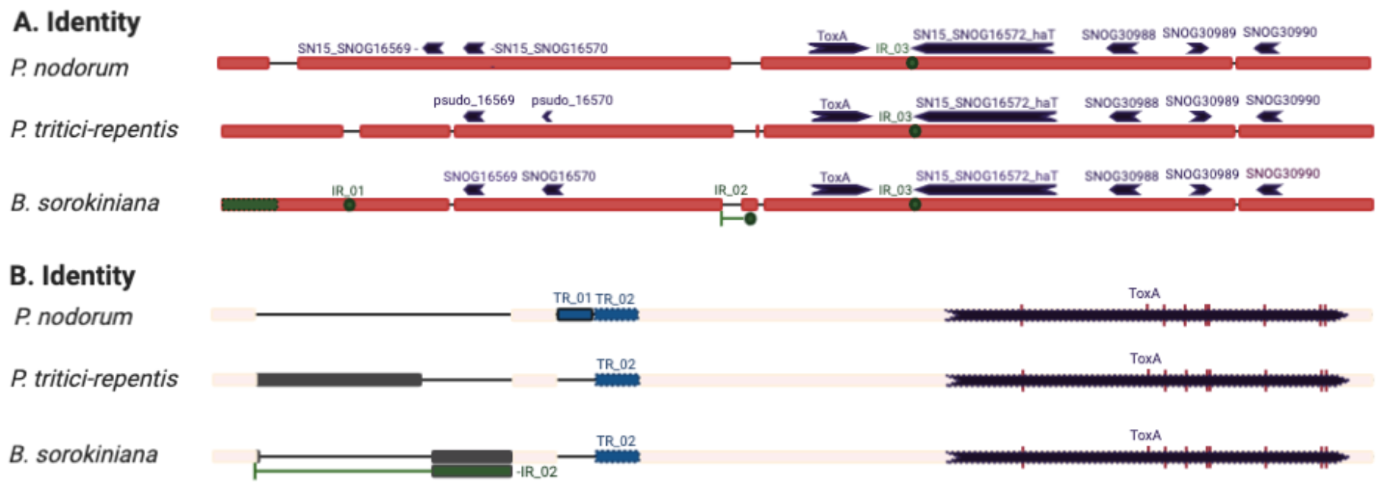 |
| --- |
| **SUPPLEMANTARY FIGURE S5|** Alignment of ToxA containing genomic region from *P. nodorum, P. tritici-repentis*, and *B. sorokiniana*. (A) Total alignment of the 12kb region among three species. This view shows the differences in richness (green line), large increases are denoted by red asterisks. The decay in identity near the edges of the region can be attributed to RIP. All annotated genes are shown using gene accession number from *P. nodorum* (green bars). (B) Differences in the promoter of ToxA due to the presence of small indels. In *P. nodorum*, a single 43bp indel is present, which is a simple repeat of the same 43bp preceding the indel (blue bars). In *P. tritici-repentis* there is a 238bp insertion, which has no homology with any of the two other pathogens. In *B. sorokiniana* there is a 148bp indel, which forms a near-perfect DNA hairpin. (Modified from Friesen et al., 2018). |
