# Supplementary material for: Genetics and breeding for resistance against four leaf spot diseases in wheat (Triticum aestivum L.)
Source: Front Plant Sci. 2023 Mar 29;14:1023824. doi: 10.3389/fpls.2023.1023824 (PMC10096043; doi:10.3389/fpls.2023.1023824)
Supplement: Supplementary file 3 [file DataSheet_3.docx]

**Additional References**

(Cited in Supplementary Tables, but not cited in the text)

Abeysekara, N. S., Friesen, T. L., Keller, B., and Faris, J. D. (2009). Identification and characterization of a novel host-toxin interaction in the wheat-Stagonospora nodorum pathosystem. *Theor. Appl. Genet.* 120, 117–126. doi: 10.1007/s00122-009-1163-6

Abeysekara, N. S., Friesen, T. L., Liu, Z., McClean, P. E., and Faris, J. D. (2010). Marker development and saturation mapping of the tan spot ptr ToxB sensitivity locus Tsc2 in hexaploid wheat. *Plant Genome* 3. doi: 10.3835/plantgenome2010.07.0017

Adhikari, T. B., Anderson, J. M., and Goodwin, S. B. (2003). Identification and molecular mapping of a gene in wheat conferring resistance to *Mycosphaerella graminicola*. *Phytopathology* 93, 1158–1164. doi: 10.1094/PHYTO.2003.93.9.1158

Adhikari, T. B., Cavaletto, J. R., Dubcovsky, J., Gieco, J. O., Schlatter, A. R., and Goodwin, S. B. (2004b). Molecular mapping of the *Stb4* gene for resistance to septoria tritici blotch in wheat. *Phytopathology* 94, 1198–1206. doi: 10.1094/ PHYTO.2004.94.11.1198

Adhikari, T. B., Gurung, S., Hansen, J. M., Jackson, E. W., and Bonman, J. M. (2012). Association mapping of quantitative trait loci in spring wheat landraces conferring resistance to bacterial leaf streak and spot blotch. *Plant Genome* 5, 1–16. doi: 10.3835/ plantgenome2011.12.0032

Adhikari, T. B., Yang, X., Cavaletto, J. R., Hu, X., Buechley, G., Ohm, H. W., et al. (2004a). Molecular mapping of Stb1, a potentially durable gene for resistance to septoria tritici blotch in wheat. *Theor. Appl. Genet*. 109, 944–953. doi: 10.1007/ s00122-004-1709-6

Ahirwar, R. N., Mishra, V. K., Chand, R., Budhlakoti, N., Mishra, D. C., Kumar, S., et al. (2018). Genome-wide association mapping of spot blotch resistance in wheat association mapping initiative (WAMI) panel of spring wheat (*Triticum aestivum* l.). *PloS One* 13, e0208196. doi: 10.1371/journal.pone.0208196

Alemu, A., Brazauskas, G., Gaikpa, D. S., Henriksson, T., Islamov, B., Jorgensen, L. N., et al. (2021). Genome-wide association analysis and genomic prediction for adult plant resistance to septoria tritici blotch and powdery mildew in winter wheat. *Front. Genet*. 12. doi: 10.3389/fgene.2021.661742

AlTameemi, R., Gill, H. S., Ali, S., Ayana, G., Halder, J., Sidhu, J. S., et al. (2021). Genome-wide association analysis permits characterization of stagonospora nodorum blotch (SNB) resistance in hard winter wheat. *Sci. Rep*. 11, 12570. doi: 10.1038/s41598- 021-91515-6

Arraiano, L. S., Chartrain, L., Bossolini, E., Slatter, H. N., Keller, B., and Brown, J. K. M. (2007). A gene in European wheat cultivars for resistance to an African isolate of *Mycosphaerella graminicola*. *Plant Pathol.* 56, 73–78. doi: 10.1111/j.1365- 3059.2006.01499.x

Arraiano, L. S., Worland, A. J., Ellerbrook, C., and Brown, J. K. M. (2001). Chromosomal location of a gene for resistance to septoria tritici blotch (*Mycosphaerella graminicola*) in the hexaploid wheat ‘synthetic 6×’. *Theor. Appl. Genet*. 103, 758–764. doi: 10.1007/s001220100668

Bainsla, N. K., Phuke, R. M., He, X., Gupta, V., Bishnoi, S. K., Sharma, R. K., et al. (2020). Genome-wide association study for spot blotch resistance in afghan wheat germplasm. *Plant Pathol*. 69, 1161–1171. doi: 10.1111/ppa.13191

Ballini, E., Tavaud, M., Ducasse, A., Sanchez, D., Paux, E., Kitt, J., et al. (2020). Genome wide association mapping for resistance to multiple fungal pathogens in a panel issued from a broad composite cross-population of tetraploid wheat *Triticum turgidum*. *Euphytica* 216, 1–17. doi: 10.1007/s10681-020-02631-9

Chartrain, L., Berry, S. T., and Brown, J. K. M. (2005a). Resistance of wheat line kavkaz-K4500 L.6.a.4 to septoria tritici blotch controlled by isolate-specific resistance genes. *Phytopathology* 95, 664–671. doi: 10.1094/PHYTO-95-0664

Chartrain, L., Joaquim, P., Berry, S. T., Arraiano, L. S., Azanza, F., and Brown, J. K. M. (2005b). Genetics of resistance to septoria tritici blotch in the Portuguese wheat breeding line TE9111. *Theor. Appl. Genet*. 110, 1138–1144. doi: 10.1007/s00122-005- 1945-4

Chartrain, L., Sourdille, P., Bernard, M., and Brown, J. K. M. (2009). Identification and location of Stb9, a gene for resistance to septoria tritici blotch in wheat cultivars courtot and tonic. *Plant Pathol.* 58, 547–555. doi: 10.1111/j.1365-3059.2008.02013.x

Chu, C.-G., Friesen, T. L., Xu, S. S., and Faris, J. D. (2008). Identification of novel tan spot resistance loci beyond the known host-selective toxin insensitivity genes in wheat. *Theor. Appl. Genet*. 117, 873–881. doi: 10.1007/s00122-008-0826-z

Cowling, S. G. (2006). Identification and mapping of host resistance genes to septoria tritici blotch of wheat. [MSc thesis] (Manitoba: University of Manitoba).

Cuthbert, R. (2011). Molecular mapping of septoria tritici blotch resistance in hexaploid wheat (*Triticum aestivum* l.) (Manitoba: University of Manitoba).

Dinglasan, E. G., Peressini, T., Marathamuthu, K. A., See, P. T., Snyman, L., Platz, G., et al. (2021). Genetic characterization of adult-plant resistance to tan spot (syn, yellow spot) in wheat. *Theor. Appl. Genet*. 134, 2823–2839. doi: 10.1007/s00122-021-03861-8

Dutta, A., Croll, D., McDonald, B. A., and Krattinger, S. G. (2021). Genome-wide association study for septoria tritici blotch resistance reveals the occurrence and distribution of Stb6 in a historic Swiss landrace collection. *Euphytica* 217, 1–6. doi: 10.1007/s10681-021-02843-7

Faris, J. D., and Friesen, T. L. (2005). Identification of quantitative trait loci for race-nonspecific resistance to tan spot in wheat. *Theor. Appl. Genet*. 111, 386–392. doi: 10.1007/s00122-005-2033-5

Faris, J. D., and Friesen, T. L. (2009). Reevaluation of a tetraploid wheat population indicates that the Tsn1-ToxA interaction is the only factor governing stagonospora nodorum blotch susceptibility. *Phytopathology* 99, 906–912. doi: 10.1094/PHYTO-99- 8-0906

Faris, J. D., Anderson, J. A., Francl, L. J., and Jordahl, J. G. (1996). Chromosomal location of a gene conditioning insensitivity in wheat to a necrosis-inducing culture filtrate from *Pyrenophora tritici-repentis*. *Phytopathology* 86, 459–463. doi: 10.1094/ Phyto-86-459

Faris, J. D., Anderson, J. A., Francl, L. J., and Jordahl, J. G. (1997). RFLP mapping of resistance to chlorosis induction by *Pyrenophora tritici-repentis* in wheat. *Theor. Appl. Genet*. 94, 98–103. doi: 10.1007/s001220050387

Faris, J. D., Li, W. L., Liu, D. J., Chen, P. D., and Gill, B. S. (1999). Candidate gene analysis of quantitative disease resistance in wheat. *Theor. Appl. Genet*. 98, 219–225. doi: 10.1007/s001220051061

Feng, J., Ma, H., and Hughes, G. R. (2004). Genetics of resistance to stagonospora nodorum blotch of hexaploid wheat. *Crop Sci.* 44, 2043–2048. doi: 10.2135/ cropsci2004.2043

Ferjaoui, S., Aouini, L., Slimane, R. B., Ammar, K., Dreisigacker, S., Schouten, H.J., et al. (2022). Deciphering resistance to *Zymoseptoria tritici* in the Tunisian durum wheat landrace accession ‘Agili39’. *BMC Genomics* 23, 372. doi: 10.1186/s12864-022-08560-2

Friesen, T. L., and Faris, J. D. (2004). Molecular mapping of resistance to *Pyrenophora tritici-repentis* race 5 and sensitivity to ptr ToxB in wheat. *Theor. Appl. Genet*. 109, 464–471. doi: 10.1007/s00122-004-1678-9

Friesen, T. L., Chu, C., Xu, S. S., and Faris, J. D. (2012). SnTox5-Snn5: a novel *Stagonospora nodorum* effector-wheat gene interaction and its relationship with the SnToxA-Tsn1 and SnTox3-Snn3-B1 interactions. *Mol. Plant Pathol.* 13, 1101–1109. doi: 10.1111/j.1364-3703.2012.00819.x

Gao, Y., Faris, J. D., Liu, Z., Kim, Y. M., Syme, R. A., Oliver, R. P., et al. (2015). Identification and characterization of the SnTox6-Snn6 interaction in the *Parastagonospora nodorum*-wheat pathosystem. *Mol. Plant Microbe Interact*. 28, 615–625. doi: 10.1094/MPMI-12-14-0396-R

Gerard, G. S., Borner, A., Lohwasser, U., and Simon, M. R. (2017). Genome-wide association mapping of genetic factors controlling septoria tritici blotch resistance and their associations with plant height and heading date in wheat. *Euphytica* 213, 27. doi: 10.1007/s10681-016-1820-1

Goodwin, S. B., and Thompson, I. (2011). Development of isogenic lines for resistance to septoria tritici blotch in wheat. *Czech J. Genet. Plant Breed*. 47, S98– S101. doi: 10.17221/3262-CJGPB

Goudemand, E., Laurent, V., Duchalais, L., Tabib Ghaffary, S. M., Kema, G. H., Lonnet, P., et al. (2013). Association mapping and meta-analysis: two complementary approaches for the detection of reliable septoria tritici blotch quantitative resistance in bread wheat (*Triticum aestivum* l.). *Mol. Breed*. 32, 563–584. doi: 10.1007/s11032-013- 9890-4

Gurung, S., Mamidi, S., Bonman, J. M., Jackson, E. W., del Rio, L. E., Acevedo, M., et al. (2011). Identification of novel genomic regions associated with resistance to *Pyrenophora tritici-repentis* races 1 and 5 in spring wheat landraces using association analysis. *Theor. Appl. Genet*. 123, 1029–1041. doi: 10.1007/s00122-011-1645-1

He, X., Azzimonti, G., Sanchez-Vidaña, M. D. R., Pereyra, S. A., Sansaloni, C., Hernandez-Anguiano, A. M., et al. (2021). Mapping for adult-plant resistance against septoria tritici blotch in a common wheat line murga. *Phytopathology* 111, 1001–1007. doi: 10.1094/PHYTO-05-20-0172-R

Hu, W., He, X., Dreisigacker, S., Sansaloni, C. P., Juliana, P., and Singh, P. K. (2019). A wheat chromosome 5AL region confers seedling resistance to both tan spot and septoria nodorum blotch in two mapping populations. *Crop J*. 7, 809–818. doi: 10.1016/ j.cj.2019.05.004

Jamil, M., Ali, A., Gul, A., Ghafoor, A., Ibrahim, A. M. H., and Mujeeb-Kazi, A. (2018). Genome-wide association studies for spot blotch (*Cochliobolus sativus*) resistance in bread wheat using genotyping-by-sequencing. *Phytopathology* 108, 1307–1314. doi: 10.1094/PHYTO-02-18-0047-R

Jing, H. C., Lovell, D., Gutteridge, R., Jenk, D., Kornyukhin, D., Mitrofanova, O. P., et al. (2008). Phenotypic and genetic analysis of the *Triticum monococcum-Mycosphaerella graminicola* interaction. *New Phytol*. 179, 1121–1132. doi: 10.1111/ j.1469-8137.2008.02526.x

John, E., Jacques, S., Phan, H. T. T., Liu, L., Pereira, D., Croll, D., et al. (2022). Variability in an effector gene promoter of a necrotrophic fungal pathogen dictates epistasis and effector-triggered susceptibility in wheat. *PloS Pathog*. 18, e1010149. doi: 10.1371/journal.ppat.1010149

Juliana, P., He, X., Poland, J., Shrestha, S., Joshi, A. K., Huerta-Espino, et al. (2022). Genome-wide association mapping indicates quantitative genetic control of spot blotch resistance in bread wheat and the favourable effects of some spot blotch loci on grain yield. *Front. Plant Sci*. 13. doi: 10.3389/fpls.2022.835095

Kalia, B., Bockus, W. W., Singh, S., Tiwari, V. K., and Gill, B. S. (2018). Mapping of quantitative trait loci for resistance to race 1 of *Pyrenophora tritici-repentis* in synthetic hexaploid wheat. *Plant Breed*. 137, 313–319. doi: 10.1111/pbr.12586

Kariyawasam, G. K. (2018). Molecular genetic characterization of ptr ToxC-Tsc1 interaction and comparative genomics of *pyrenophora tritici-repentis* [Ph.D. thesis] (North Dakota: North dakota University).

Kariyawasam, G. K., Carter, A. H., Rasmussen, J. B., Faris, J., Xu, S. S., Mergoum, M., et al. (2016). Genetic relationships between race-nonspecific and race-specific interactions in the wheat-*Pyrenophora tritici-repentis* pathosystem. *Theor. Appl. Genet*. 129, 897–908. doi: 10.1007/s00122-016-2670-x

Kariyawasam, G. K., Hussain, W., Easterly, A., Guttieri, M., Belamkar, V., Poland, J., et al. (2018). Identification of quantitative trait loci conferring resistance to tan spot in a biparental population derived from two Nebraska hard red winter wheat cultivars. *Mol. Breed*. 38, 140. doi: 10.1007/s11032-018-0901-3

Kaur, J., Kaur, J., Dhillon, G. S., Kaur, H., Singh, J., Bala, R., et al. (2021). Characterization and mapping of spot blotch in *Triticum durum–Aegilops speltoides* introgression lines using SNP markers. *Front. Plant Sci.* 12. doi: 10.3389/ fpls.2021.650400

Kidane, Y. G., Hailemariam, B. N., Mengistu, D. K., Fadda, C., Pe, M. E., and Dell’Acqua, M. (2017). Genome-wide association study of septoria tritici blotch resistance in Ethiopian durum wheat landraces. *Front. Plant Sci*. 8. doi: 10.3389/ fpls.2017.01586

Kollers, S., Rodemann, B., Ling, J., Korzun, V., Ebmeyer, E., Argillier, O., et al. (2013). Genetic architecture of resistance to septoria tritici blotch (*Mycosphaerella graminicola*) in European winter wheat. *Mol. Breed*. 32, 411–423. doi: 10.1007/s11032- 013-9880-6

Kumar, U., Joshi, A. K., Kumar, S., Chand, R., and Roder, M. S. (2009). Mapping of resistance to spot blotch disease caused by *Bipolaris sorokiniana* in spring wheat. *Theor. Appl. Genet*. 118, 783–792. doi: 10.1007/s00122-008-0938-5

Kumar, U., Joshi, A. K., Kumar, S., Chand, R., and Röder, M. S. (2010). Quantitative trait loci for resistance to spot blotch caused by *Bipolaris sorokiniana* in wheat (*T. aestivum* L.) lines ‘Ning 8201’ and ‘Chirya 3’. Mol. Breed. 26, 477–491. doi: 10.1007/ s11032-009-9388-2

Lamari, L., Bernier, C. C., and Smith, R. B. (1991). Wheat genotypes that develop both tan necrosis and extensive chlorosis in response to isolates of *Pyrenophora tritici-repentis*. *Plant Dis*. 75, 121–122. doi: 10.1094/PD-75-0121

Langlands-Perry, C., Cuenin, M., Bergez, C., Krima, S. B., Gelisse, S., Sourdille, P., et al. (2021). Resistance of the wheat cultivar ‘Renan’to septoria leaf blotch explained by a combination of strain specific and strain non-specific QTL mapped on an ultra-dense genetic map. *Genes* 13, 100. doi: 10.3390/genes13010100

Li, H. B., Yan, W., Liu, G. R., Wen, S. M., and Liu, C. J. (2011). Identification and validation of quantitative trait loci conferring tan spot resistance in the bread wheat variety Ernie. *Theor. Appl. Genet*. 122, 395–403. doi: 10.1007/s00122-010-1455-x

Liu, Y., Zhang, L., Thompson, I. A., Goodwin, S. B., and Ohm, H. W. (2013). Molecular mapping re-locates the Stb2 gene for resistance to septoria tritici blotch derived from cultivar veranopolis on wheat chromosome 1BS. *Euphytica* 190, 145–156. doi: 10.1007/s10681-012-0796-8

Liu, Z. H., Faris, J. D., Meinhardt, S. W., Ali, S., Rasmussen, J. B., and Friesen, T. L. (2004b). Genetic and physical mapping of a gene conditioning sensitivity in wheat to a partially purified host-selective toxin produced by *Stagonospora nodorum*. *Phytopathology* 94, 1056–1060. doi: 10.1094/PHYTO.2004.94.10.1056

Liu, Z. H., Friesen, T. L., Ling, H., Meinhardt, S. W., Oliver, R. P., Rasmussen, J. B., et al. (2006). The Tsn1-ToxA interaction in the wheat-*Stagonospora nodorum* pathosystem parallels that of the wheat-tan spot system. *Genome* 49, 1265–1273. doi: 10.1139/g06-088

Liu, Z. H., Zurn, J. D., Kariyawasam, G., Faris, J. D., Shi, G., Hansen, J., et al. (2017). Inverse gene-for-gene interactions contribute additively to tan spot susceptibility in wheat. *Theor. Appl. Genet*. 130, 1267–1276. doi: 10.1007/s00122-017-2886-4

Louriki, S., Rehman, S., El Hanafi, S., Bouhouch, Y., Al-Jaboobi, M., Amri, A., et al. (2021). Identification of resistance sources and genome-wide association mapping of septoria tritici blotch resistance in spring bread wheat germplasm of ICARDA. *Front. Plant Sci.* 12. doi: 10.3389/fpls.2021.6001

Lozano-Ramirez, N., Dreisigacker, S., Sansaloni, C. P., He, X., Islas, S. S., Perez Rodriguez, P., et al. (2022a). Genome-wide association study for resistance to tan spot in synthetic hexaploid wheat. *Plants* 11, 433. doi: 10.3390/plants11030433

Lozano-Ramirez, N., Dreisigacker, S., Sansaloni, C. P., He, X., Sandoval-Islas, J. S., Perez-Rodriguez, P., et al. (2022b). Genome-wide association study for spot blotch resistance in synthetic hexaploid wheat. *Genes* 13, 1387. doi: 10.3390/genes13081387

Ma, H., and Hughes, G. R. (1995). Genetic control and chromosomal location of *Triticum timopheevii*-derived resistance to septoria nodorum blotch in durum wheat. *Genome* 38, 332–338. doi: 10.1139/g95-042

Mahboubi, M., Talebi, R., Mehrabi, R., Mohammad Naji, A., Maccaferri, M., and Kema, G. H. J. (2022). Genetic analysis of novel resistance sources and genome-wide association mapping identified novel QTLs for resistance to *Zymoseptoria tritici*, the causal agent of septoria tritici blotch in wheat. *J. Appl. Genet.* 63(3), 429-445. doi: 10.1007/ s13353-022-00696-x

McCartney, C. A., Brule-Babel, A. L., Lamari, L., and Somers, D. J. (2003). Chromosomal location of a race-specific resistance gene to *Mycosphaerella graminicola* in the spring wheat ST6. *Theor. Appl. Genet*. 107, 1181–1186. doi: 10.1007/s00122-003-1359-0

Mekonnen, T., Sneller, C. H., Haileselassie, T., Ziyomo, C., Abeyo, B. G., Goodwin, S. B., et al. (2021). Genome-wide association study reveals novel genetic loci for quantitative resistance to septoria tritici blotch in wheat (*Triticum aestivum* l.). *Front. Plant Sci*. 12. doi: 10.3389/fpls.2021.671323

Miedaner, T., Zhao, Y., Gowda, M., Longin, C. F. H., Korzun, V., Ebmeyer, E., et al. (2013). Genetic architecture of resistance to septoria tritici blotch in European wheat. *BMC Genomics* 14, 858. doi: 10.1186/1471-2164-14-858

Muqaddasi, Q. H., Zhao, Y., Rodemann, B., Plieske, J., Ganal, M. W., and Roder, M. S. (2019). Genome-wide association mapping and prediction of adult stage septoria tritici blotch infection in European winter wheat via high-density marker arrays. *Plant Genome* 12, 180029. doi: 10.3835/plantgenome2018.05.0029

Odilbekov, F., Armoniene, R., Koc, A., Svensson, J., and Chawade, A. (2019). GWAS-assisted genomic prediction to predict resistance to septoria tritici blotch in nordic winter wheat at seedling stage. *Front. Genet*. 10. doi: 10.3389/fgene.2019.01224

Orolaza, N. P., Lamari, L., and Balance, G. M. (1995). Evidence of a host-specific chlorosis toxin from *Pyrenophora tritici-repentis*, the causal agent of tan spot of wheat. *Phytopathology* 85, 1282–1287. doi: 10.1094/Phyto-85-1282

Pankaj, Y. K., Kumar, R., Gill, K. S., and Nagarajan, R. (2022). Discovering QTLs related to spot blotch disease in spring wheat (*Triticum aestivum* l.) genome. *Aus. Plant Pathol*. 51, 441–452. doi: 10.1007/s13313-022-00873-w

Pavan, S. (2011). Exploring recessive resistance to the powdery mildew disease. [PhD thesis] (Wageningen: Wageningen University).

Perez-Lara, E., Semagn, K., Tran, V. A., Ciechanowska, I., Chen, H., Iqbal, M., et al. (2017). Population structure and genome wide association analysis of resistance to disease and insensitivity to ptr toxins in Canadian spring wheat using 90K SNP array. *Crop Sci*. 57, 1522–1539. doi: 10.2135/cropsci2016.10.0859

Phuke, R. M., He, X., Juliana, P., Bishnoi, S. K., Singh, G. P., Kabir, M. R., et al. (2020). Association mapping of seedling resistance to tan spot (*Pyrenophora tritici-repentis* race 1) in CIMMYT and south Asian wheat germplasm. *Front. Plant Sci*. 11. doi: 10.3389/fpls.2020.01309

Radecka-Janusik, M., and Czembor, P. C. (2014). Genetic mapping of quantitative trait loci (QTL) for resistance to septoria tritici blotch in a winter wheat cultivar liwilla. *Euphytica* 200, 109–125. doi: 10.1007/s10681-014-1157-6

Raman, R., Milgate, A. W., Imtiaz, M., Tan, M.-K., Raman, H., Lisle, C., et al. (2009). Molecular mapping and physical location of major gene conferring seedling resistance to septoria tritici blotch in wheat. *Mol. Breed.* 24, 153–164. doi: 10.1007/s11032-009- 9280-0

Running, K. L. D., Momotaz, A., Kariyawasam, G. K., Zurn, J. D., Acevedo, M., Carter, A. H., et al. (2022). Genomic analysis and delineation of the tan spot susceptibility locus Tsc1 in wheat. *Front. Plant Sci*. 13. doi: 10.3389/fpls.2022

Saini, D. K., Chahal, A., Pal, N., Srivastava, P., and Gupta, P. K. (2022). Meta-analysis reveals consensus genomic regions associated with multiple disease resistance in wheat (*Triticum aestivum* l.). *Mol. Breed.* 42, 11. doi: 10.1007/s11032-022-01282-z

Sharma, P., Mishra, S., Singroha, G., Kumar, R. S., Singh, S. K., and Singh, G. P. (2022). Phylogeographic diversity analysis of *Bipolaris sorokiniana* (Sacc.) shoemaker causing spot blotch disease in wheat and barley. *Genes* 13, 2206. doi: 10.3390/ genes13122206

Shi, G., Friesen, T. L., Saini, J., Xu, S. S., Rasmussen, J. B., and Faris, J. D. (2015). The wheat Snn7 gene confers susceptibility on recognition of the *Parastagonospora nodorum* necrotrophic effector SnTox7. *Plant Genome* 8, 1–10. doi: 10.3835/ plantgenome2015.02.0007

Singh, P. K., Crossa, J., Duveiller, E., Singh, R. P., and Djurle, A. (2016). Association mapping for resistance to tan spot induced by *Pyrenophora tritici-repentis* race 1 in CIMMYTs historical bread wheat set. *Euphytica* 207, 515–525. doi: 10.1007/s10681- 015-1528-7

Singh, P. K., Gonzalez-Hernandez, J. L., Mergoum, M., Ali, S., Adhikari, T. B., Kianian, S. F., et al. (2006). Identification and molecular mapping of a gene conferring resistance to *Pyrenophora tritici-repentis* race 3 in tetraploid wheat. *Phytopathology* 96, 885–889. doi: 10.1094/PHYTO-96-0885

Singh, P. K., He, X., Sansaloni, C. P., Juliana, P., Dreisigacker, S., Duveiller, E., et al. (2018). Resistance to spot blotch in two mapping populations of common wheat is controlled by multiple QTL of minor effects. *Int. J. Mol. Sci.* 19, 4054. doi: 10.3390/ ijms19124054

Singh, P. K., Mergoum, M., Ali, S., Adhikari, T. B., and Hughes, G. R. (2008a). Genetic analysis of resistance to *Pyrenophora tritici-repentis* races 1 and 5 in tetraploid and hexaploid wheat. *Phytopathology* 98, 702–708. doi: 10.1094/PHYTO-98-6-0702

Singh, P. K., Mergoum, M., Gonzalez-Hernandez, J. L., Ali, S., Adhikari, T. B., Kianian, S. F., et al. (2008b). Genetics and molecular mapping of resistance to necrosis inducing race 5 of *Pyrenophora tritici-repentis* in tetraploid wheat. *Mol. Breed*. 21, 293– 304. doi: 10.1007/s11032-007-9129-3

Singh, S., Gaurav, S. S., Vasistha, N. K., Kumar, U., Joshi, A. K., Mishra, V. K., et al. (2022). Genetics of spot blotch resistance in bread wheat (*Triticum aestivum* l.) using five models for GWAS. *Front.Plant Sci*. 13. doi: 10.3389/fpls.2022.1036064

Singh, S., Mishra, V. K., Kharwar, R. N., Budhlakoti, N., Ahirwar, R. N., Mishra, D. C., et al. (2020). Genetic characterization for lesion mimic and other traits in relation to spot blotch resistance in spring wheat. *PloS One* 15, e0240029. doi: 10.1371/ journal.pone.0240029

Stock, W. S., Brule-Bable, A. L., and Penner, G. A. (1996). A gene for resistance to a necrosis–inducing isolate of *Pyrenophora tritici-repentis* located on 5BL of Triticum aestivum cv. Chinese spring. Genome 39, 598–604. doi: 10.1139/g96-075

Sun, X. C., Bockus, W., and Bai, G.-H. (2010). Quantitative trait loci for resistance to *Pyrenophora tritici-repentis* race 1 in a Chinese wheat. *Phytopathology* 100, 468–473. doi: 10.1094/PHYTO-100-5-0468

Tabib Ghaffary, S. M., Faris, J. D., Friesen, T. L., Visser, R. G. F., van der Lee, T. A. J., Robert, O., et al. (2012). New broad-spectrum resistance to septoria tritici blotch derived from synthetic hexaploid wheat. *Theor. Appl. Genet*. 124, 125–142. doi: 10.1007/s00122-011-1692-7

Tabib Ghaffary, S. M., Robert, O., Laurent, V., Lonnet, P., Margale, E., van der Lee, T. A. J., et al. (2011). Genetic analysis of resistance to septoria tritici blotch in the French winter wheat cultivars balance and Apache. *Theor. Appl. Genet.* 123, 741–754. doi: 10.1007/s00122-011-1623-7

Tadesse, W., Hsam, S. L. K., and Zeller, F. J. (2006a). Evaluation of common wheat cultivars for tan spot resistance and chromosomal location of a resistance gene in the cultivar ‘Salamouni’. *Plant Breed*. 125, 318–322. doi: 10.1111/j.1439-0523.2006.01243.x

Tadesse, W., Hsam, S. L. K., Wenzel, G., and Zeller, F. J. (2006b). Identification and monosomic analysis of tan spot resistance genes in synthetic wheat lines (*Triticum turgidum* l. × *Aegilops tauschii* coss.). *Crop Sci*. 46, 1212–1217. doi: 10.2135/ cropsci2005.10-0396

Tadesse, W., Schmolke, M., Hsam, S. L., Mohler, V., Wenzel, G., and Zeller, F. J. (2007). Molecular mapping of resistance genes to tan spot (*Pyrenophora tritici-repentisrace* 1) in synthetic wheat lines. *Theor. Appl. Genet*. 114, 855–862. doi: 10.1007/s00122- 006-0484-y

Tadesse, W., Schmolke, M., Hsam, S. L., Mohler, V., Wenzel, G., and Zeller, F. J. (2010). Chromosomal location and molecular mapping of a tan spot resistance gene in the winter wheat cultivar red Chief. *J. Appl. Genet*. 51, 235–242. doi: 10.1007/ BF03208853

Tomar, V., Singh, D., Dhillon, G. S., Singh, R. P., Poland, J., Joshi, A. K., et al. (2020). New QTLs for spot blotch disease resistance in wheat (*Triticum aestivum* l.) using genome-wide association mapping. *Front. Genet*. 11. doi: 10.3389/fgene.2020.613217

Vagndorf, N., Nielsen, N. H., Edriss, V., Andersen, J. R., Orabi, J., Jorgensen, L. N., et al. (2017). Genome wide association study reveals novel quantitative trait loci associated with resistance towards septoria tritici blotch in north European winter wheat. *Plant Breed*. 136, 474–482. doi: 10.1111/pbr.12490

Yang, N., McDonald, M. C., Solomon, P. S., and Milgate, A. W. (2018). Genetic mapping of Stb19, a new resistance gene to *Zymoseptoria tritici* in wheat. *Theor. Appl. Genet*. 131, 2765–2773. doi: 10.1007/s00122-018-3189-0

Yang, N., Ovenden, B., Baxter, B., McDonald, M. C., Solomon, P. S., and Milgate, A. (2022). Multi-stage resistance to *Zymoseptoria tritici* revealed by GWAS in an Australian bread wheat (*Triticum aestivum* l.) diversity panel. *Front. Plant Sci.* 13. doi: 10.3389/fpls.2022.9901510

Yates, S., Mikaberidze, A., Krattinger, S. G., Abrouk, M., Hund, A., Yu, K., et al. (2019). Precision phenotyping reveals novel loci for quantitative resistance to septoria tritici blotch. *Plant Phenomics* 2019, 3285904. doi: 10.34133/2019/3285904

Zhang, W., Zhu, X., Zhang, M., Shi, G., Liu, Z., and Cai, X. (2019). Chromosome engineering-mediated introgression and molecular mapping of novel *Aegilops speltoides*-derived resistance genes for tan spot and septoria nodorum blotch diseases in wheat. *Theor. Appl. Genet*. 132, 2605–2614. doi: 10.1007/s00122-019-03374-5

Zhang, Z., Friesen, T. L., Xu, S. S., Shi, G., Liu, Z., Rasmussen, J. B., et al. (2011). Two putatively homoeologous wheat genes mediate the recognition of SnTox3 to confer effector-triggered susceptibility to *Stagonospora nodorum*. *Plant J.* 65, 27–38. doi: 10.1111/j.1365-313X.2010.04407.x

Zhu, Z., Bonnett, D., Ellis, M., Singh, P., Heslot, N., Dreisigacker, S., et al. (2014). Mapping resistance to spot blotch in a CIMMYT synthetic-derived bread wheat. *Mol. Breed.* 34, 1215–1228. doi: 10.1007/s11032-014-0111-6
